# Selective photocatalytic oxidative cleavage of terminal alkynes to carboxylic acids within a water-soluble Pd_6_ nanocage

**DOI:** 10.1039/d5sc08202a

**Published:** 2026-01-05

**Authors:** Pranay Kumar Maitra, Valiyakath Abdul Rinshad, Neal Hickey, Partha Sarathi Mukherjee

**Affiliations:** a Department of Inorganic and Physical Chemistry, Indian Institute of Science Bangalore-560012 India psm@iisc.ac.in; b Department of Chemical and Pharmaceutical Sciences, University of Trieste Trieste 34127 Italy

## Abstract

The selective oxidative cleavage of terminal alkynes to carboxylic acids under mild, environmentally benign conditions remains a major challenge in catalysis due to the diverse reaction profile of terminal alkynes. Herein, we report a cavity-mediated UV light-driven oxidation of terminal alkynes to the corresponding carboxylic acids in aqueous medium using a water-soluble Pd_6_ nanocage. This transformation proceeds without the need for ozonolysis or precious metal oxide catalysts. Mechanistic investigations indicate that generation of hydroxyl radicals mediates the oxidative cleavage of terminal alkynes. Notably, we achieved a chemo-selective transformation of arylalkynes bearing methyl substituents, which are typically susceptible to oxidation under confinement. Furthermore, the recyclability of the cage in the catalysis was demonstrated over multiple cycles with the retention of catalytic activity. This work highlights the potential of selective photo-induced oxidative transformations of substrates using coordination cages in aqueous medium.

## Abstract 2

A water-soluble Pd_6_L_4_ coordination cage, presenting a distorted octahedral geometry, was utilized for selective oxidative cleavage of terminal alkynes to the corresponding carboxylic acids in aqueous medium.

## Graphical abstract

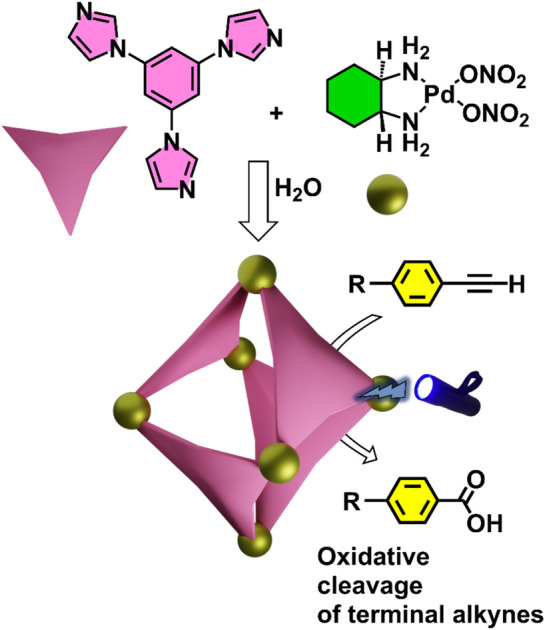

## Introduction

Nature employs confined nanospaces to mediate organic transformations by precisely controlling the reaction environment. In such catalytic processes, non-covalent interactions such as hydrogen bonding, π–π interactions, and van der Waals forces play a vital role in governing both the rate and selectivity of the reactions.^1^ Inspired by these natural processes, researchers have designed synthetic systems that attempt to replicate such confinement effects.^2^ Examples include extended porous materials like metal–organic frameworks (MOFs) and covalent organic frameworks (COFs),^3^ as well as discrete molecular architectures such as organic cages, capsules, macrocycles^4^ and coordination cages.^5^ Among these systems, coordination cages formed *via* coordination-driven self-assembly are particularly attractive due to their structural precision, solubility, and customizable internal cavities.^6^ These hydrophobic cavities are capable of selectively hosting guest molecules and have been applied in various domains including molecular recognition,^7^ chemical sensing,^8^ stabilization of reactive intermediates,^9^ selective separations,^10^ and light energy capture.^11^ Additionally, metal–organic cages have been well explored as nanovessels for carrying out a wide variety of organic transformations, including Diels–Alder cycloaddition,^12^ Knoevenagel condensation,^13^ and oxidative reactions.^14^

Motivated by the advances in confinement mediated catalysis, there is a growing interest in developing green and recyclable supramolecular catalytic systems for oxidative transformations in aqueous medium.^15^ In particular, the oxidative cleavage of terminal alkynes to yield carboxylic acids is a synthetically valuable reaction, which provides key intermediates in pharmaceuticals, agrochemicals, and polymer synthesis.^16^ Generally, the synthesis of carboxylic acids from alkynes involves either ozonolysis or two-step dihydroxylation followed by oxidative cleavage of diols with sodium periodate. Similarly, the direct conversion of alkynes to carboxylic acids can be achieved using osmium, manganese, rhodium, iridium, and other metals or metal oxides under harsh\inert conditions which limit their practical applications and sustainability.^17^ Recent research efforts have shifted towards exploring milder, oxygen-based oxidative strategies; however, the development of efficient and reusable catalytic systems for direct terminal alkyne oxidative cleavage under mild and aqueous conditions remains a significant challenge. Coordination cages, with their modularity, water solubility, and ability to stabilize reactive intermediates through non-covalent interactions, represent a promising platform to address this gap in oxidative catalysis.

**Scheme 1:**
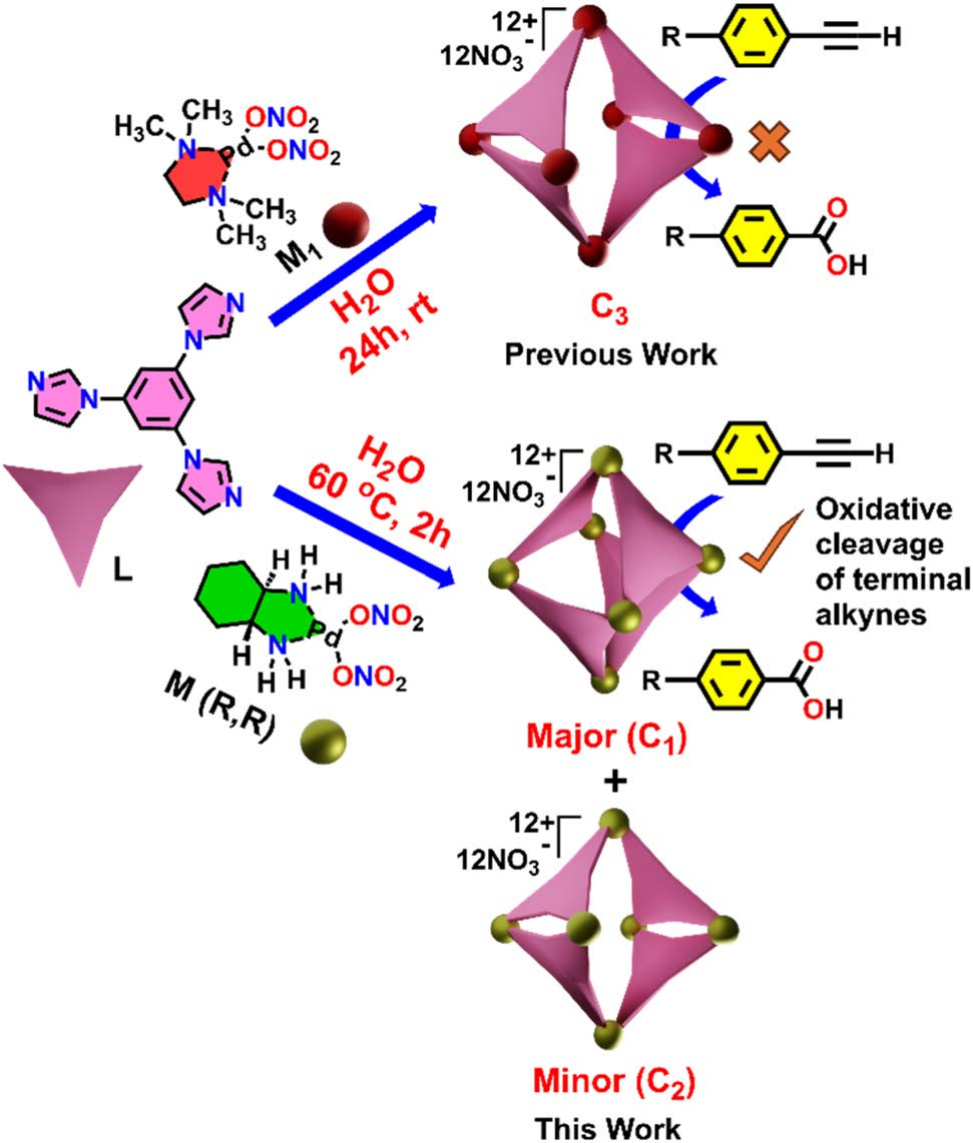
Schematic representation of the synthesis of cage C_1_ and its use in catalysing terminal alkyne oxidation.

Herein, we describe a highly efficient and selective oxidative cleavage of terminal alkynes to the corresponding carboxylic acids using a water-soluble Pd(ii) coordination cage (C_1_) under mild reaction conditions. The Pd_6_ nanocage C_1_ was constructed *via* coordination-driven self-assembly of a C_3_-symmetric benzene-triimidazole ligand (L) with a 90° *cis*-(1*R*,2*R*-dch)Pd(NO_3_)_2_ acceptor unit (M) (1*R*,2*R*-dch) = *trans*-1*R*,2*R*-cyclohexane-1,2-diamine, resulting predominantly in a well-defined unexpected distorted octahedral structure (C_1_) as the major product (Scheme 1) instead of the expected double-square architecture (C_3_) that was formed using the *N*,*N*,*N*′,*N*′-tetramethylethylene-1,2-diamine *cis*-blocked Pd(ii)-acceptor.^13*a*^ In addition to the octahedral cage C_1_, the double-square cage (C_2_) was also observed to form in minor amounts (∼8%) in the self-assembly reaction. The unusual octahedral structure of the cage C_1_ was unambiguously confirmed by single-crystal X-ray diffraction (SCXRD), which revealed the presence of a hydrophobic internal cavity. Host–guest binding studies demonstrated that C_1_ can effectively encapsulate a range of terminal alkynes, driven by hydrophobic and π–π interactions within the confined interior. Under UV irradiation this cage mediates the oxidative cleavage of ethynylbenzene to benzoic acid in high yield (Scheme 1). Moreover, we achieved a chemo-selective transformation of alkyne to the corresponding carboxylic acid in the presence of an alkyl group. Such a selective oxidation of only the alkyne group without affecting the alkyl group is noteworthy as alkyl substituents in aromatic rings are known to undergo oxidation in the presence of air under confinement.^15*f*^ This transformation proceeds without the need for harsh oxidants, which showcases the potential of coordination cages as recyclable nanoreactors for environmentally benign oxidative transformations.

## Results and discussion

**Fig. 1:**
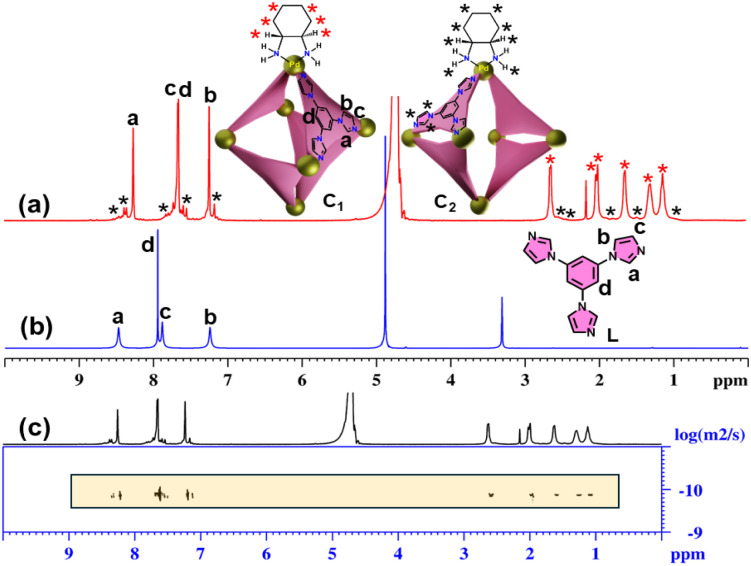
^1^H NMR spectra of the (a) self-assembled product (NO_3_^−^ analogue in D_2_O, 298 K); (b) L (CD_3_OD, 298 K) and (c) ^1^H-DOSY NMR spectrum of the self-assembled product (D_2_O, 298 K).

The tridentate ligand L was prepared according to a previously described method.^13*a*^ Self-assembly of L with a 90° *cis*-blocked Pd(ii) acceptor (M) in a 2 : 3 molar ratio in water at 70 °C for 12 hours resulted in a clear, colourless solution. The ^1^H NMR spectrum of the product displayed three sharp signals in the aromatic region, closely resembling those of the free ligand L, which indicates the formation of a highly symmetric coordination cage (C_1_) (Fig. 1a and b).

In addition to the major signals, a set of minor peaks with two-fold splitting of the original signals was also observed, suggesting the formation of a minor self-assembled product (C_2_) with lower symmetry. A DOSY NMR spectrum of the self-assembled product(s) showed a single, well-defined diffusion coefficient for both the major and minor species, indicating that they are similar in size (Fig. 1c).

**Fig. 2:**
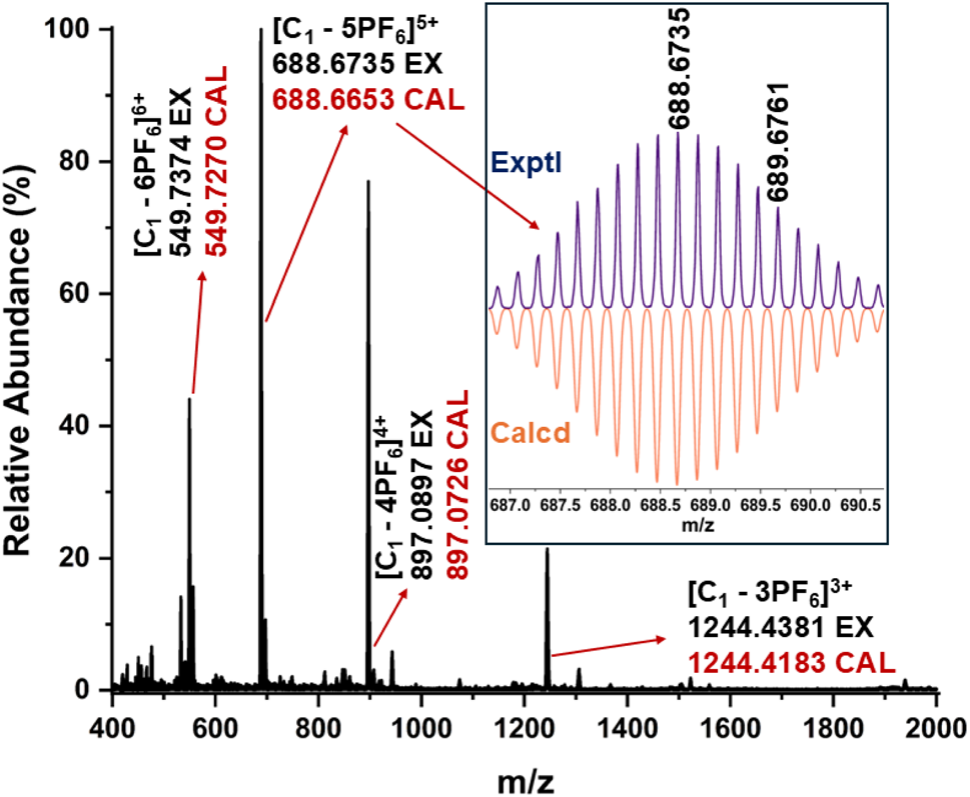
Electrospray ionization mass spectrum of the PF_6_^−^ analogue of the self-assembled product in acetonitrile. (Inset) Experimental and calculated isotopic distribution pattern of the [C_1_-5PF_6_]^5+^ fragment.

The stoichiometry and composition of the self-assembled product were conclusively established through electrospray ionization mass spectrometry (ESI-MS) analysis of the hexafluorophosphate (PF_6_^−^) analogue of the self-assembled product. The resulting spectrum exhibited several prominent peaks corresponding to multiply charged species, notably at *m*/*z* values of 549.7374, 688.6735, 897.0897, and 1244.4381. These peaks were assigned to the ionized forms [C_1_-6PF_6_]^6+^, [C_1_-5PF_6_]^5+^, [C_1_-4PF_6_]^4+^, and [C_1_-3PF_6_]^3+^, respectively (Fig. 2). The observed isotopic patterns were in agreement with the simulated patterns, thereby validating the proposed molecular structure (Fig. S5). These results confirmed the M_6_L_4_ stoichiometry of the cage, *i.e.*, combination of six metal acceptors (M) with four ligands (L). The M_6_L_4_ stoichiometry can adopt either an octahedral or a double-square structure. The nature of the NMR spectrum of the major self-assembled product confirms that the resultant major product is an octahedron (C_1_). In an octahedron, the ligand peaks of the cage are expected to show one set of peaks, similar to what is observed for the ligand, due to the higher symmetry. The two-fold splitting of the minor peaks observed in the ^1^H NMR spectrum of the self-assembled product mixture showed a relative integration of 2 : 1, which is a characteristic feature of double-square coordination cages (C_2_) (Fig. S6).

**Fig. 3:**
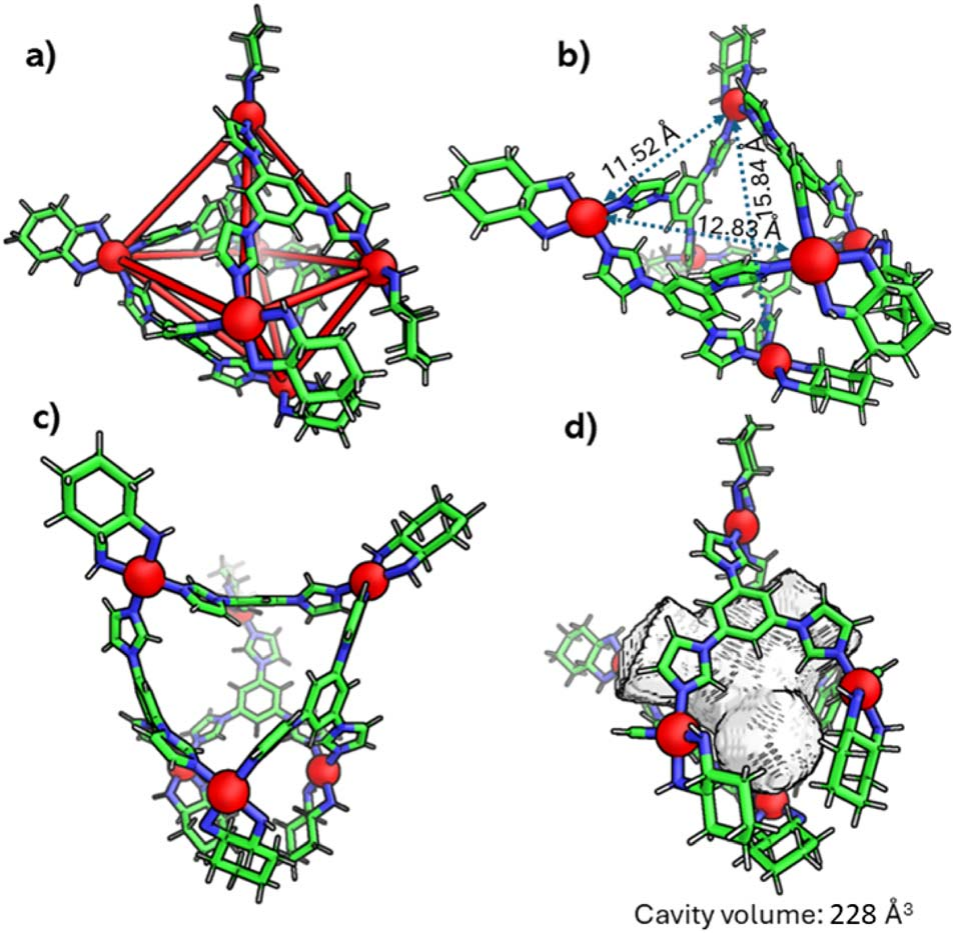
X-ray crystal structure of C_1_ (CCDC no. 2455229). (a) Distorted octahedral shape of the cage; (b and c) side views of the cage and (d) cavity space of the cage (colour codes: C, green; N, blue; and Pd, red).

Finally, the geometry of C_1_ was unambiguously confirmed through single-crystal X-ray diffraction (Fig. 3 and S8–S11). Crystals appropriate for X-ray diffraction were obtained by the slow vapor diffusion of acetone into a saturated aqueous solution of the self-assembled product, in which nitrate (NO_3_^−^) was present as the counterion. Data collection was carried out using synchrotron radiation. The compound C_1_ crystallized in the triclinic space group *P*_1_ and exhibited a distorted octahedral geometry. The asymmetric unit contains two crystallographically independent molecules which exhibit approximate inversion symmetry. However, analysis of the electron density indicated that *P*_1_ is the correct space group, as converting from *P*_1_ to *P-*_1_ gave rise to spurious electron density. With regard to the absolute configuration of the M acceptors (1*R*,2*R*-dch), the calculated Flack parameter was inconclusive.^19*a*^ However, Bayesian statistical analysis of Bijvoet pairs performed with PLATON confirmed the correctness of the assigned configuration (Table S2).^19*b*^

The crystal structure revealed that four acceptor (M) units of each metallacage were arranged in a distorted square planar configuration. For one of the cages the distances of the four palladium ions from the mean plane of these same four palladium ions are in the range of 0.55–0.80 Å (see also Fig. S9). Two additional acceptor units are situated at the axial positions, at vertical distances of 6.82 Å and 8.70 Å from the mean plane of the four equatorial palladium ions. The Pd⋯Pd distance between these two positions is 15.84 Å, thus the two ions are horizontally displaced with respect to the equatorial mean plane. The overall result is a rather distorted octahedral architecture as shown in Fig. 3a–c and S9. Four ligands (L) occupy alternating faces of the octahedron. Additionally, the structure features a substantial internal cavity with triangular openings (as shown in Fig. 3a–c and quantified in the SI). The volume of the hydrophobic cavity of C_1_ was calculated to be 228 Å^3^ (Fig. 3d) using the MoloVol software.^20^ The second metallacage exhibits very similar geometric characteristics. Further geometric details of the cages are outlined in the SI. Geometry optimizations and single-point energy evaluations (B3LYP/def2-SVP, PCM) reveal that the octahedral M6L4 cage is 33.41 kcal mol^−1^ more stable than the corresponding double-square assembly when constructed from the rigid *cis*-(1*R*,2*R*-dch) Pd(ii) corners. This substantial energy difference accounts for the formation of the octahedral topology (Fig. S51a). Furthermore, the square-planar Pd(ii) coordination ideally requires *cis* N–Pd–N angles of 90°. In the octahedral cage C_1_, constructed from the rigid 1*R*,2*R*-cyclohexanediamine-based Pd(ii) acceptor, the observed N–Pd–N angles are 84.19° and 91.30°, indicating only modest deviations from the ideal square planar geometry. These small distortions are readily accommodated within the octahedral M6L4 topology. In contrast, the DFT-optimized double-square cage C_2_ constructed from the same rigid acceptor exhibits N–Pd–N angles of 79.96° and 103.65°, corresponding to large deviations from 90° and leading to significant geometric strain (Fig. S51b). Additional insight is obtained by comparing the dihedral angles of the cyclohexanediamine-based Pd(ii) acceptor in C_1_ and C_2_. The dihedral angles are 54.39° in octahedral C_1_ and 56.45° in double-square C2, showing only a very small difference. This minimal variation confirms the high rigidity of the 1*R*,2*R*-cyclohexanediamine chelate, which prevents the substantial conformational adjustment required for the double-square topology and therefore favours formation of the rigid octahedral architecture as the major product.

By contrast, in our previously reported double-square architecture assembled using the TMEDA–Pd(ii) acceptor (C_3_), the dihedral angle is only 4.03°, while a recently reported octahedral architecture (C_4_) using the same TMEDA–Pd(ii) acceptor shows a dihedral angle of 54.09°. These large variations clearly demonstrate that the TMEDA–Pd(ii) acceptor is highly flexible, compared to the rigid 1*R*,2*R*-cyclohexanediamine acceptor (Fig. S51b).^13*a*,*b*^

### Guest encapsulation studies

**Fig. 4:**
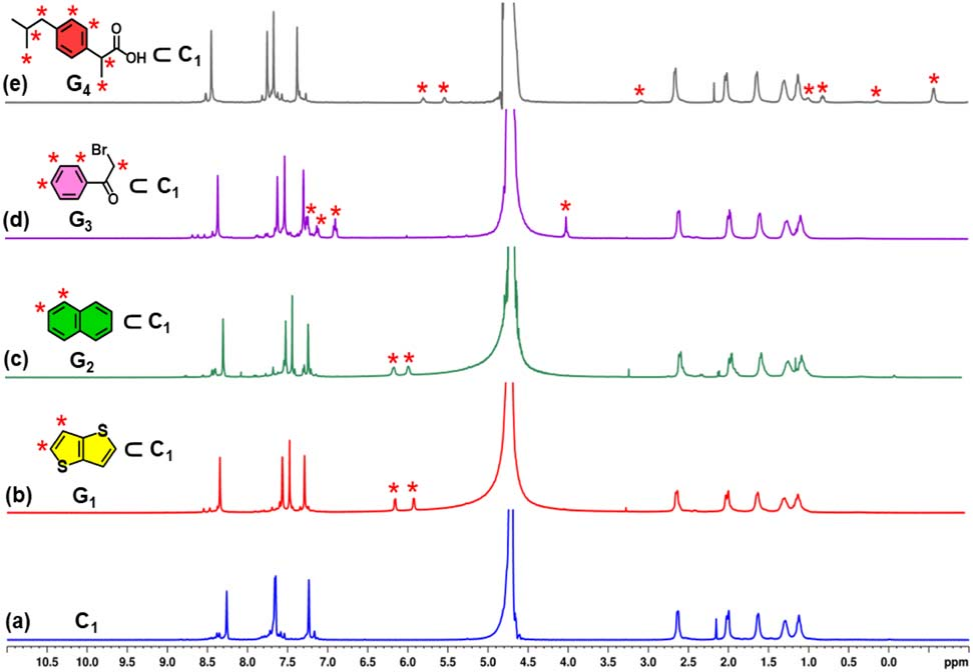
^1^H NMR spectra of (a) C_1_; (b) G_1_⊂C_1_; (c) G_2_⊂C_1_; (d) G_3_⊂C_1_ and (e) G_4_⊂C_1_ in D_2_O showing the change in NMR signals upon guest encapsulation by C_1_. Star-marked peaks correspond to the encapsulated guests.

The encapsulation ability of host C_1_ was examined using aromatic guests G_1_, G_2_, G_3_ and G_4_ as model guest molecules (Fig. 4). An excess amount of solid thieno[3,2-*b*]thiophene (G_1_) was introduced into an aqueous solution of C_1_, followed by stirring at room temperature for 8 hours. This resulted in the formation of a cloudy mixture, which was subsequently centrifuged, and the clear supernatant was collected for further analysis. The resulting solution (G_1_⊂C_1_) displayed new proton signals in the ^1^H NMR spectrum, appearing at 6.14 and 5.90 ppm (Fig. 4b), which correspond to guest signals. Additionally, the protons on the imidazole rings were shifted downfield, while the benzene protons of the ligand shifted upfield, due to the host–guest interaction with the guest molecule. These chemical shift changes indicated successful encapsulation of the guest molecule within the cage cavity. Further confirmation of internal binding was obtained from the ^1^H DOSY NMR spectrum of the G_1_⊂C_1_ complex in D_2_O, which exhibited a single diffusion coefficient (log *D* = −9.844) (Fig. S13), consistent with the formation of a host–guest assembly. Evidence for spatial proximity between the host and guest was observed in the ^1^H–^1^H NOESY spectrum (Fig. S14), where cross-peaks were observed between protons of the C_1_ ligand and the aromatic protons of G_1_. Host–guest stoichiometry was established through signal integration of the NMR spectra, indicating a 1 : 2 ratio between C_1_ and G_1_ (Fig. S12). Additional analysis showed that C_1_ encapsulated one molecule of G_2_, two molecules of G_3_ and one molecule of G_4_ in separate experiments. (Fig. S15–S18).

Having a water-soluble flexible octahedral host in hand, we sought to explore its potential for mediating chemical transformations in aqueous medium. Recent research by Dasgupta *et al.* demonstrated that photoactivation of terminal alkynes within a water-soluble, rigid Pd_6_ octahedral nanocage (TPT Cage) occurs *via* a host–guest charge transfer (CT) mechanism, ultimately yielding C–C coupling products.^21^ In that system, photoinduced CT is followed by proton loss to generate a neutral radical intermediate, which is stabilized by the electron-deficient triazine-based ligand framework. Motivated by this strategy, we examined whether our structurally flexible, imidazole-based octahedral cage (C_1_) could mediate photo-oxidation of the encapsulated substrates in a catalytic fashion. In contrast to the triazine system, C_1_ incorporates a relatively electron-rich benzene-derived core, which we envisioned might alter the reactivity pathway of encapsulated terminal alkynes. Remarkably, instead of facilitating radical-mediated C–C coupling, C_1_ directed the selective oxidation of terminal alkynes to carboxylic acids.

### Oxidation of terminal alkynes to corresponding carboxylic acids within C_1_

**Fig. 5:**
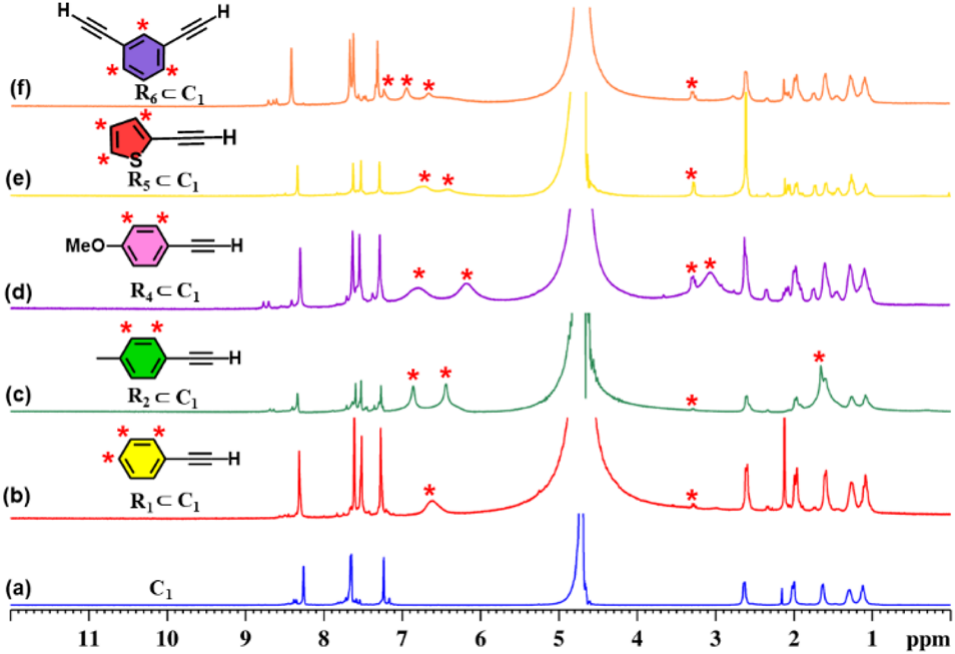
^1^H NMR stack plots of (a) C_1_; (b) R_1_⊂C_1_; (c) R_2_⊂C_1_; (d) R_4_⊂C_1_; (e) R_5_⊂C_1_, and (f) R_6_⊂C_1_, in D_2_O showing the change in NMR signals upon guest encapsulation by C_1_. Star-marked peaks correspond to the encapsulated guests.

Initially, we investigated whether ethynylbenzene (R_1_) could be encapsulated in the cavity of C_1_. An excess amount of R_1_ was added to an aqueous solution of C_1_ and stirred for 12 hours. The resulting solution was centrifuged and subjected to NMR analysis. The ^1^H NMR spectrum showed a downfield shift of the host peaks with the appearance of guest peaks in the aromatic region (Fig. 5b). The ^1^H DOSY NMR spectrum showed a single diffusion coefficient for the host and guest protons, which confirmed the formation of an inclusion complex (Fig. S20). Moreover, the ^1^H–^1^H NOESY spectrum (Fig. S21) displayed distinct cross-peaks between the host and guest protons, providing strong evidence for guest encapsulation within the cage cavity. Furthermore, the host–guest stoichiometry of the R_1_⊂C_1_ complex was determined using ^1^H NMR titration experiments. Cage C_1_ was dissolved in D_2_O, while a stock solution of the guest R_1_ was prepared in MeOD-d_4_. Incremental additions of 10 µL aliquots of the R_1_ stock solution were made to the aqueous solution of C_1_, and NMR spectra were recorded immediately after each addition (Fig. S26a).

During the titration, continuous shifts in the proton signals of both the host and guest were observed, indicating a fast exchange process on the NMR timescale. The stoichiometric ratio was established using a Job's plot, which confirmed a 1 : 2 host : guest ratio (Fig. S27a). Similarly, we have studied the encapsulation of derivatives of terminal alkynes (R_2_–R_6_) like that of R_1_ (Fig. S22–S25).

The photocatalytic efficiency of cage C_1_ was initially evaluated using ethynylbenzene (R_1_) in water under 390 nm light irradiation. Remarkably, the targeted oxidative cleavage product P_1_ was formed in 99% yield (GC yield) after 2 hours under aerobic conditions (Table 1, entry 2), highlighting the strong photocatalytic performance of C_1_. The individual acceptor and ligand components were used under the same conditions, but no product formation was detected (Table 1, entries 3 and 4). Alternative light sources, including blue LEDs (450 nm) and white LEDs, also failed to drive the oxidation, yielding no detectable products (Table 1, entry 6). Likewise, performing the reaction under thermal conditions or in the absence of light or photocatalyst led to no conversion (Table 1, entries 5, 7 and 8), confirming the necessity of both light and cage C_1_ for the reaction to proceed. In addition, we prepared a water-soluble derivative of the ligand. Here, the three imidazole moieties of ligand L were methylated and converted to the nitrate salt to prepare the water-soluble cationic form (L_1_) (Scheme S1). Notably, L_1_ was unable to catalyse the reaction, highlighting the essential role of the cage C_1_ in catalytic activity (Table 1, entry 13).

**Table 1:** Oxidation of ethynylbenzene to benzoic acid^a^

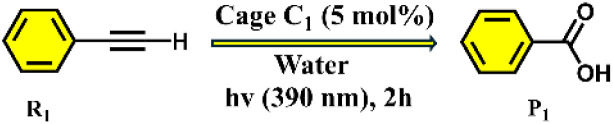							
Entry	Solvent	Atmosphere	Temperature	Light (nm)	Time (h)	Cage/acceptor/ligand	Yield (%)
1	H_2_O	O_2_	r. t.	390	2	Cage C_1_	>99
2	H_2_O	Air	r. t.	390	2	Cage C_1_	>99
3	H_2_O	Air	r. t.	390	2	Acceptor (M)	0
4	H_2_O	Air	r. t.	390	2	Ligand (L)	0
5	H_2_O	Air	r. t.	Dark	2	Cage C_1_	0
6	H_2_O	Air	r. t.	White/blue	2	Cage C_1_	0
7	H_2_O	Air	Heat	—	2	Cage C_1_	0
8	H_2_O	Air	r. t.	390	2	BLANK	0
9	H_2_O	Air	r. t.	390	2	C_1_ + TEMPO	4
10	H_2_O	Air	r. t.	390	2	C_1_ + p-BQ	10
11	H_2_O	Air	r. t.	390	2	C_1_ + *t*-butanol	20
12	H_2_O	Air	r. t.	390	2	C_3_	0
13	H_2_O	Air	r. t.	390	2	L_1_	0
14	CH_3_OH	Air	r. t.	390	2	L	0

a Reactions were carried out in water at room temperature for 2 h under an ambient atmosphere using 5 mol% cage (C_1_) and 390 nm LED irradiation. Yields were determined by GC-MS after extraction with EtOAc.

To gain mechanistic insights, we initially recorded the UV-vis absorption spectra of the host–guest complexes. A broad band emerged in the absorption spectra for the host–guest complexes, which was attributed to charge-transfer (CT) interactions between the guest and host molecules (Fig. S28). Furthermore, we carried out control experiments to study the reactive species generated during this transformation. In the presence of 2,2,6,6-tetramethylpiperidinyloxyl (TEMPO), a known radical scavenger, the reaction yielded only trace amounts of the target product (Table 1, entry 9), implying a radical-mediated mechanism. Similarly, addition of *p*-benzoquinone, a quencher for superoxide radicals, suppressed product formation significantly (Table 1, entry 10). To assess the role of hydroxyl radicals as the reaction intermediate, tertiary butanol was used as an efficient hydroxyl radical trap under standard reaction conditions. This led to a decrease in yield (Table 1, entry 11). The analysis of EPR results further confirmed the presence of the superoxide radical and hydroxyl radical in the reaction mixture (Fig. S47). The above-mentioned results clearly indicate that the reaction proceeds through the superoxide, which further reacts with water to yield the hydroxyl radical.^18^

Based on our experimental observations, we propose a catalytic mechanism in which the supramolecular cage operates like an enzyme, facilitating chemical transformations within its confined space (Fig. S48). Initially, the cage forms a strong charge transfer interaction with ethynylbenzene, as verified by the UV-vis study. Upon irradiation with 390 nm UV light, an electron transfer (ET) occurs from the electron-rich aromatic guest to the cage interior, generating a terminal alkyne radical cation. Concurrently, molecular oxygen (O_2_) is converted to a superoxide anion (O_2_˙^−^), which subsequently reacts with water to yield hydroxyl radicals (˙OH) and hydroxide ions (OH^−^). Subsequently, the terminal alkyne radical cation is attacked by ˙OH to generate a cationic intermediate (ii), which then undergoes nucleophilic attack by OH^−^ to produce intermediate (iii). This intermediate experiences a β-scission process, resulting in the formation of an aldehyde, which was confirmed through GC-MS analysis after 1 hour of irradiation of 1-ethynyl-4-methylbenzene (Fig. S44). Continued oxidation of this aldehyde ultimately leads to the formation of a carboxylic acid.

To investigate whether the trace amount of the double-square cage (C_2_) formed in the self-assembly could contribute to the observed reactivity, control experiments were performed using a known double-square cage (C_3_) that contains an *N*,*N*,*N*′,*N*′-tetramethylethylene-1,2-diamine blocked *cis*-Pd(ii) acceptor. C_3_ was used for this control experiment because C_2_ couldn't be isolated as the major species. The structurally similar (like C_2_) double-square cage, C_3_, previously reported^13*a*^ based on the same ligand framework, was synthesized (Fig. S45) and tested under identical catalytic conditions (Fig. S46). No product formation was observed with the C_3_ cage, indicating that it is not catalytically active (Table 1, entry 12). To elucidate the distinct reactivities of the octahedral cage C_1_ and the double-square cage C_3_, we carried out UV-vis analyses with R_1_. Whereas C_1_ exhibits a clear charge-transfer band upon addition of phenylacetylene, C_3_ shows no such response, despite possessing the same tritopic ligand. This unequivocally highlights the critical influence of cage topology in enabling substrate activation. The flexible double-square geometry of C_3_ fails to provide the spatial and electronic confinement needed to stabilise the charge-transfer state, preventing initiation of the catalytic cycle. Consequently, C_3_ remains catalytically inactive, and no oxidation product was observed (Fig. S28b and c). These results strongly suggest that the catalytic activity arises exclusively from the C_1_ cage, which features an octahedral geometry. Thus, neither the C_2_ nor the C_3_ cage appears to participate in the catalytic cycle.

### Different chemical reactivity of terminal alkynes within the C_1_ and TPT cage

**Fig. 6:**
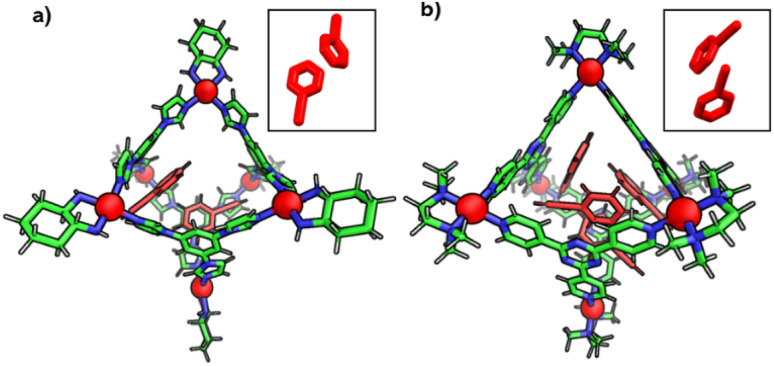
Optimized structures of preorganization of R_1_ inside the cavity of (a) C_1_ (inset) showing the opposite orientation of two R_1_ and the (b) TPT cage (inset) showing the same orientation of two R_1_ (optimized using the xTB programme).

To understand the reason behind the tendency of the octahedral TPT cage (containing the 2,4,6-tris(4-pyridyl)-triazine ligand) to promote the C–C coupling product^21^ and C_1_ to form the oxidized product, the structures of the host–guest complexes (*i.e.*, R_1_⊂TPTand R_1_⊂C1) were optimized by semiempirical methods with the xTB programme (Fig. 6).^22^ In the case of C_1_, the optimized structure revealed that two phenylacetylene molecules are accommodated within the cavity, where they are stabilized primarily through π–π interactions with the electron-rich benzene walls of the cage, with a binding energy of −32.0 kcal mol^−1^ (Fig. S52). Notably, the two guest molecules adopt an opposite orientation on either side of the cavity. This spatial arrangement precludes the close approach of the alkyne moieties and effectively rules out radical–radical recombination, thereby disfavouring C–C bond formation. Instead, the electronic environment of C_1_ channels reactivity toward oxidative transformations of the encapsulated substrates. In sharp contrast, the optimized R_1_⊂TPTcomplex revealed that up to four phenylacetylene molecules can be preorganized within the rigid, electron-deficient triazine-based cavity (Fig. S53). Stabilization in this case arises from π–π stacking interactions between the guest molecules and the triazine walls. Importantly, pairs of phenylacetylenes are oriented in the same direction relative to one another within the cavity, which brings their reactive sites into closer spatial proximity. Upon photoinduced charge transfer and subsequent proton loss, the resulting neutral radical intermediates are stabilized by the electron-deficient triazine framework. The combination of guest preorganization and radical stabilization provides a structural basis for the observed propensity of the TPT cage to promote C–C coupling (Fig. 6b).

Moreover, in our previous work, we demonstrated that a water-soluble cage could promote the oxidation of methyl substituents in aromatic rings to carboxylic acids under mild, photocatalytic conditions.^15*f*^ Motivated by this reactivity, we sought to expand the scope to more terminal alkynes, specifically methyl-substituted phenylacetylenes, to probe whether both the methyl and the alkyne moieties might undergo oxidation inside the cage. We observed that only the alkyne functionality was oxidized, while the methyl group in the aromatic ring remained intact (Table 2, entries 1 and 2). This chemo-selectivity suggests that the confined, electron-rich environment of C_1_ uniquely channels the oxidation pathway towards alkyne activation. Extending this study to di-alkynyl substrates revealed that both alkyne groups could be cleanly transformed into carboxylic acids, further underscoring the distinct reactivity imparted by the cage (Table 2, entry 5). With the optimal conditions established, we explored the substrate scope for the transformation of terminal alkynes to the corresponding carboxylic acids using C_1_. A range of substituted alkynes bearing groups such as methyl-(R_2_ and R_3_), methoxy-(R_4_), thio-(R_5_), and diethynyl (R_6_) functionalities were tested, and the corresponding carboxylic acids were obtained in excellent yields, demonstrating the broad applicability of the system. Importantly, the water-soluble cage photocatalyst C_1_ could be efficiently recovered post-reaction by simple in-flask extraction with ethyl acetate. This allowed for direct reuse of both the catalyst and aqueous solvent for subsequent reactions by adding a fresh batch of alkyne. The aqueous medium containing C_1_ retained its catalytic activity over at least five consecutive cycles without measurable loss of efficiency (Fig. S49). This highlights the sustainability of the system, where both the photocatalyst and reaction medium are recyclable without the need for removal from the reaction setup (Fig. S50).

**Table 2:** Photocatalytic oxidation of various ethynyl aromatics^a^

Entry	Substrate	Conditions	Time	Product	Yield (%)
1	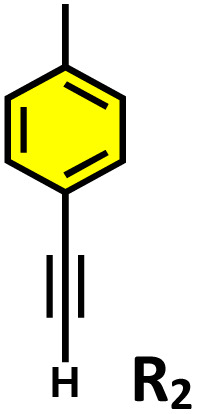	C_1_ (5 mol%) *hv* (390 nm)	2 h	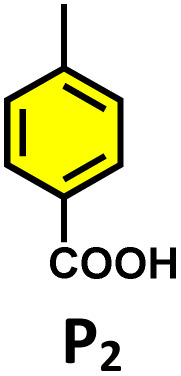	>99
2	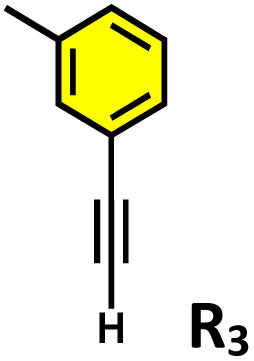	C_1_ (5 mol%) *hv* (390 nm)	2 h	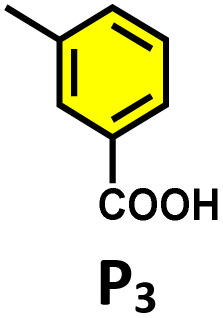	>99
3	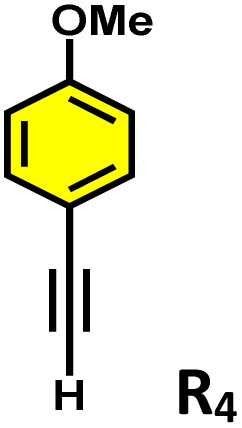	C_1_ (5 mol%) *hv* (390 nm)	2 h	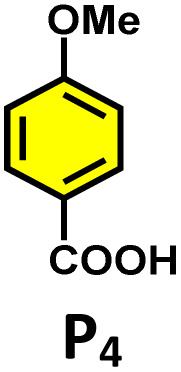	>99
4	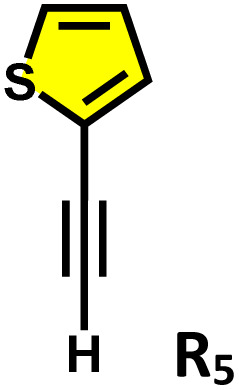	C_1_ (5 mol%) *hv* (390 nm)	2 h	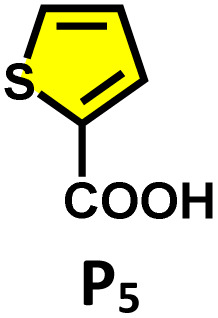	>99
5	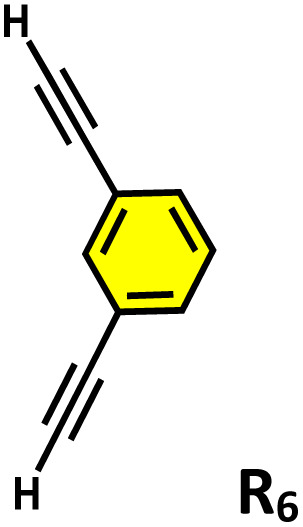	C_1_ (5 mol%) *hv* (390 nm)	2 h	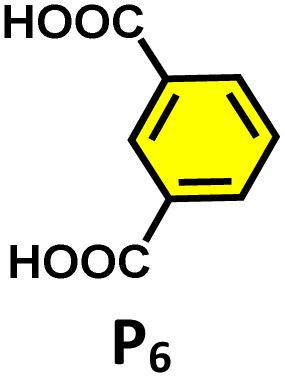	>99

a All reactions were carried out in water at room temperature for 2 h under an ambient atmosphere using 5 mol% cage (C_1_) and 390 nm LED irradiation. Yields were determined by GC-MS after extraction with EtOAc.

To evaluate whether the proposed catalytic mechanism also applies to internal alkynes, we examined prop-1-yn-1-ylbenzene as a representative substrate under identical supramolecular cage reaction conditions. The reaction proceeded slowly (requiring ∼10 h) and benzoic acid was obtained (Fig. S41c). UV-vis measurements indicate that this internal alkyne forms a weak charge-transfer complex with the cage C_1_, which likely accounts for the reduced reactivity (Fig. S28e). These results suggest that, although internal alkynes can undergo oxidation within the cage, the efficiency is diminished due to less favourable charge-transfer interaction.

## Conclusion

We have successfully synthesized a new water-soluble Pd_6_ octahedral nanocage (C_1_) *via* coordination-driven self-assembly of a *cis*-blocked Pd(ii) 90° acceptor (M) [M = *cis*-(1*R*,2*R*-dch)Pd(NO_3_)_2_] with a triimidazole-based donor ligand (L). Formation of such an octahedral structure from a *cis*-blocked 90° acceptor, employing this tri-imidazole ligand L, is very unusual as the similar [4 + 6] self-assembly of L with the commonly-used 90° acceptor, *cis*-(tmeda)Pd(NO_3_)_2_, is known to form double-square architecture.^13*a*^ Single-crystal X-ray diffraction confirms the formation of an unusual octahedral cage architecture as the major self-assembled product. This octahedral cage showed excellent encapsulation of various aromatic molecules and terminal alkynes. Importantly, C_1_ catalyses the selective photooxidative cleavage of terminal alkynes to carboxylic acids under mild aqueous conditions. The role of the cage cavity of C_1_ in this transformation was established by using an isomeric Pd_6_ cage (C_3_), which differs in geometry, and a water-soluble ligand (L_1_), both of which failed to produce the desired product under the same conditions. Guest encapsulation within the nanocage cavity promotes the formation of a charge-transfer complex, which upon photoexcitation generates a radical cation on the confined alkyne substrate as a reactive intermediate. Moreover, the difference in the reactivity of terminal alkynes in the cavities of C_1_ and known TPT cages was examined by computational analysis. The theoretical studies showed that a combination of guest preorganization and radical stabilization is responsible for the difference in the observed chemical reactivity of terminal alkynes within the cavity of C_1_ and TPT cage. Since the Pd(ii) centers in the nanocage do not directly engage in the chemical transformation, our findings establish a blueprint for developing supramolecular hosts for photocatalytic oxidations.

## Author contributions

P. S. M. and P. K. M. devised the project and designed the experiments. P. K. M. carried out the experimental work together with V. A. R., and analyzed the data. N. H. collected and solved the crystallographic data. All authors contributed to the writing of the manuscript.

## Conflicts of interest

There are no conflicts to declare.

## Supplementary Material

SC-017-D5SC08202A-s001

SC-017-D5SC08202A-s002

## Data Availability

All data are provided in the supplementary information (SI) and additional data can be made available upon request. CCDC 2455229 contains the supplementary crystallographic data for this paper.^23^ Supplementary information: NMR spectra, ESI-MS, CIF file, optimized structures, and experimental details (PDF). See DOI: https://doi.org/10.1039/d5sc08202a.
