# Supplementary material for: Selective photocatalytic oxidative cleavage of terminal alkynes to carboxylic acids within a water-soluble Pd6 nanocage
Source: Chem Sci. 2026 Jan 5;17(9):4487–95. doi: 10.1039/d5sc08202a (PMC12809706; doi:10.1039/d5sc08202a)
Supplement: SC-017-D5SC08202A-s001 [file SC-017-D5SC08202A-s001.pdf]

Supplementary Information (SI) for Chemical Science.
This journal is © The Royal Society of Chemistry 2026

Supporting Information

Selective Photocatalytic Oxidative Cleavage of Terminal Alkynes to Carboxylic Acids within a Water-Soluble Pd_6 Nanocage

Pranay Kumar Maitra,[a] *Valiyakath Abdul Rinshad,*[a] *Neal Hickey,*[b] *Partha Sarathi Mukherjee*[a]*

[a]Department of Inorganic and Physical Chemistry, Indian Institute of Science, Bangalore 560012, India.

E-mail: psm@iisc.ac.in

[b]Department of Chemical and Pharmaceutical Sciences, University of Trieste, Trieste 34127, Italy

Contents:

**1.**	**Materials and Methods**	**S2**
**2.**	**Synthesis and Characterization of Cage C_1**	**S3-S6**
**3.**	**X-ray Crystallographic Analysis of C_1**	**S7-S14**
**4.**	**Host-Guest Encapsulation Study of C_1**	**S15-S21**
**5.**	**NMR Titrations of R_1 with C_1**	**S22-S23**
**6.**	**General Procedure for Photocatalysis and characterization of photocatalyzed product**	**S23-S34**
**7.**	**Geometry Optimization**	**S35-S58**

1. Materials and Methods

All chemicals and solvents were purchased from commercial sources and used directly without further purification. The NMR spectra of the newly prepared materials were recorded using Bruker 500 and 400 MHz spectrometers in deuterated solvents. The chemical shifts in the spectra are reported relative to TMS (0.0 ppm) or proton resonance resulting from the incomplete deuteration of D_2O (δ = 4.79 ppm), MeOD (3.31 ppm), $CDCl_3$ (7.26 ppm), and CH_3CN-d_3 (1.94 ppm). Electrospray ionization mass spectrometric (ESI-MS) analyses were carried out on Waters Q-TOF instrument in standard spectroscopic grade solvents. GC-MS data were acquired using GCMS-QP2010 SE SHIMADZU system. Electronic absorption spectra were recorded on Shimadzu UV-2600 UV−visible spectrophotometer. 100W LED lamp (390 nm) was used for the photocatalytic reaction. Ligand **L** was prepared according to a literature procedure. [S1]

2. Synthetic Procedures

2.1 Synthesis of Cage C$_1$

In an 8 mL glass vial, **L** (30.00 mg, 0.108 mmol) was added to the solution of *cis*-[(1*R*, 2*R*)-cyclohexane-1,2-diamine $Pd(NO_3)_2$] (56.13 mg, 0.162 mmol) (**M**) in 4 mL H_2O. The reaction mixture was stirred at 70 °C for 2 hours. The solution was centrifuged, and the clear supernatant was evaporated and triturated with acetone to yield a white precipitate. Yield of isolated pure cage **C$_1$:** 71.2 mg (82%). ^{1}H NMR (D_2O, 400 MHz): δ (ppm) = 8.28 (s, 1H), 7.66 (d, 1H), 7.65 (s, 1H), 7.24 (d, 1H,), 2.64 (d, 2H), 2.02 (d, 2H), 1.62 (s, 2H), 1.29 (s, 2H), 1.12 (s, 2H). ESI-MS (PF_6^- analogue of **C$_1$**) (m/z) = 1244.4381 [**C$_1$**-3PF_6]$^{3+}$, 897.0897 [**C$_1$**-4PF_6]$^{4+}$, 688.6735 [**C$_1$**-5PF_6]$^{5+}$, 549.7374 [C$_1$- 6PF_6]$^{6+}$.

2.2 Synthesis of Cage C$_3$

In a 4 mL glass vial, **L** (5.50 mg, 20.00 μmol) was added to a 1 mL aqueous solution of *cis*-[(tmeda)$Pd(NO_3)_2$] [tmeda = *N,N,N',N'*-tetramethylethylenediamine] (**M$_1$**) (10.40 mg, 30.00 μmol). The mixture was stirred at room temperature for 24 hours which gave a clear yellowish-green solution. The solvent was then removed completely, and the resultant solid was washed with acetone. The resulting solid was dried under vacuum to obtain **C$_3$** in pure form. Yield: 14.8 mg (94%). ^{1}H NMR of the self-assembled product matched well with previously reported literature results.[S2]

2.3 Synthesis of L$_1$

A 100 mL round bottom flask was charged with **L** (0.01 g, 0.03 mmol) and iodomethane (1 ml) in DMF. This mixture was heated to 80 °C and kept for overnight under N_2. A white precipitate was observed upon completion of the reaction. This precipitate was washed with $CHCl_3$ and diethyl ether, and this was dissolved in 4 mL methanol. To this solution $AgNO_3$ (0.018 g, 1.09 mmol) was added and the resulting mixture was stirred in dark for 2 h. The resulting precipitate was separated by filtration, and the filtrate was dried to afford white solid (0.016 g, 87%). ^{1}H NMR (D_2O, 400 MHz): δ (ppm) = 9.36 (s, 1H), 8.17 (s, 1H). 7.96 (s, 1H), 7.65 (s, 1H), 3.99 (s, 3H) ppm.

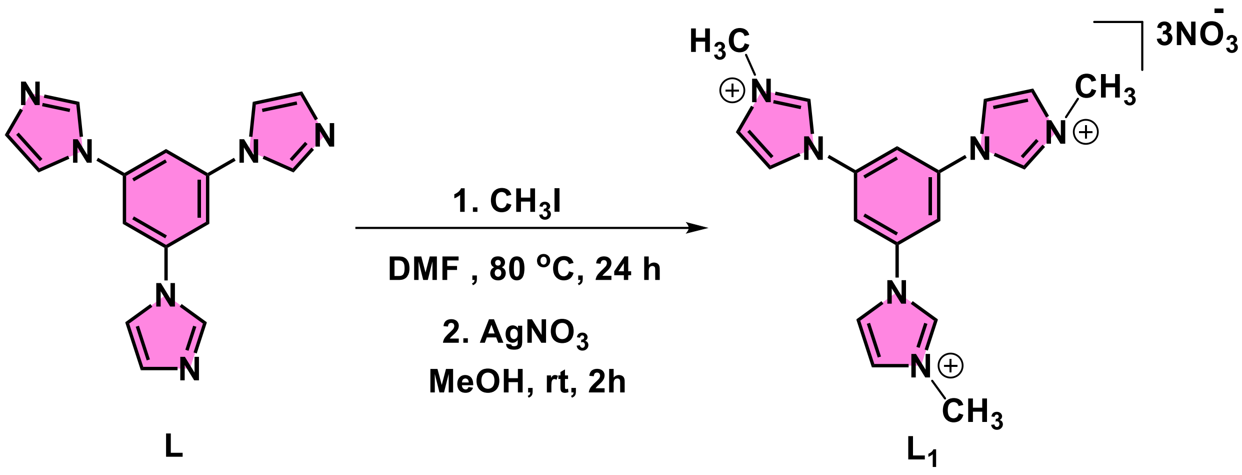

Scheme S1. Schematic representation of the synthesis of **L**$_1$.

3. Spectral Characterization of Ligand and Cages

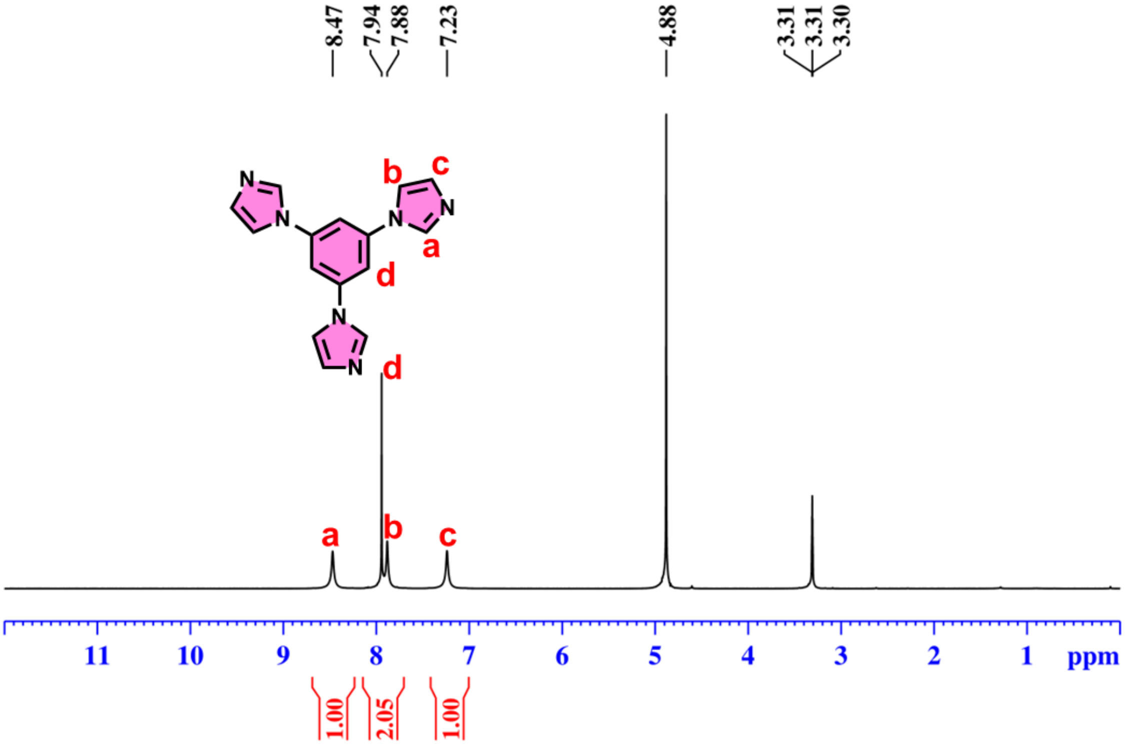

Figure S1. ^{1}H NMR spectra of **L** (CD_3OD, 298 K).

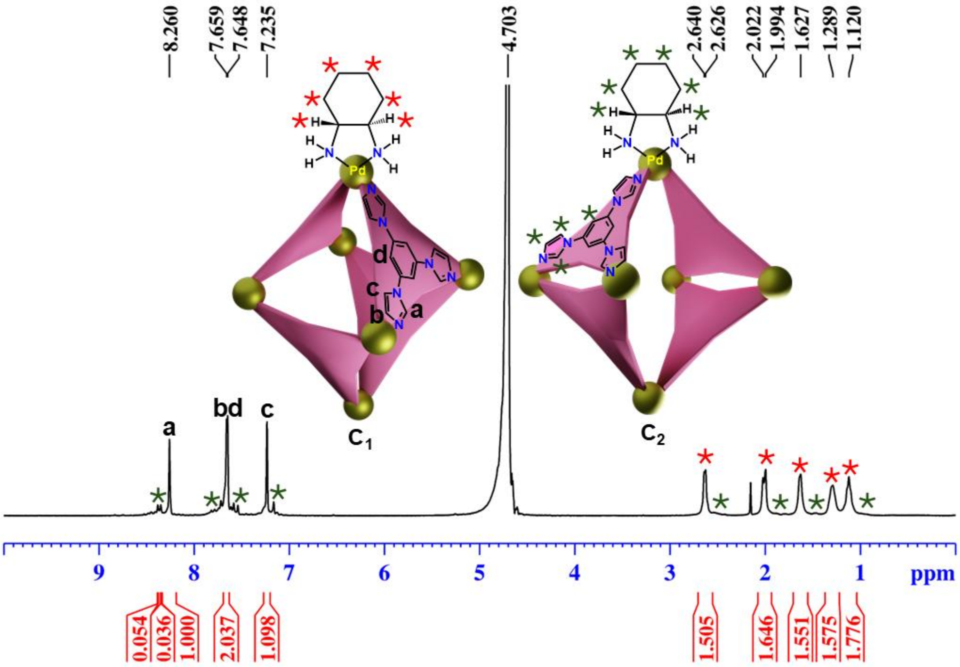

Figure S2a. ^{1}H NMR spectrum of cage $\mathbf{C_1}$ (NO_3^-) (D_2O, 298 K). The isomeric $\mathbf{C_2}$ was denoted by green stars. ($\mathbf{C_1}/\mathbf{C_2}$) = (91.74/8.26)

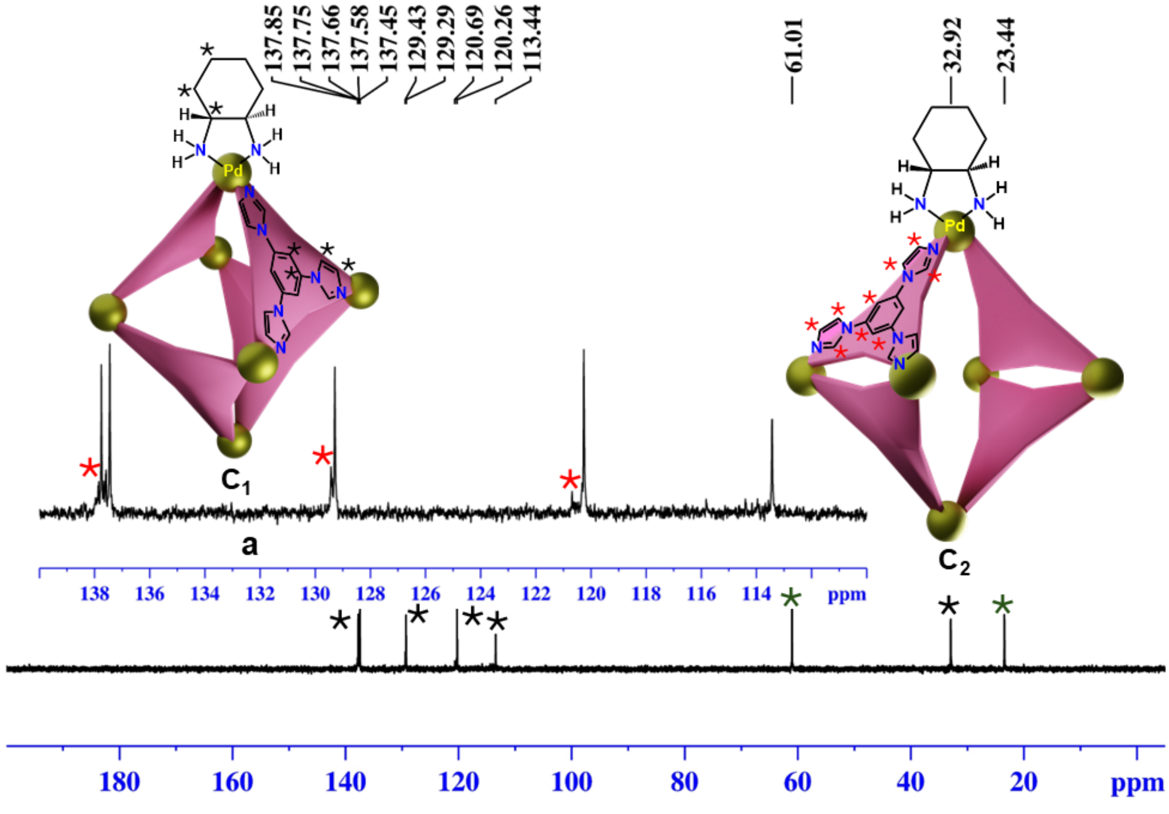

Figure S2b. ^{13}C NMR spectrum of cage $\mathbf{C_1}$ (NO_3^-) (D_2O, 298 K). The isomeric $\mathbf{C_2}$ was denoted by red stars. Top spectrum is the zoomed view in the range of 140-110 ppm.

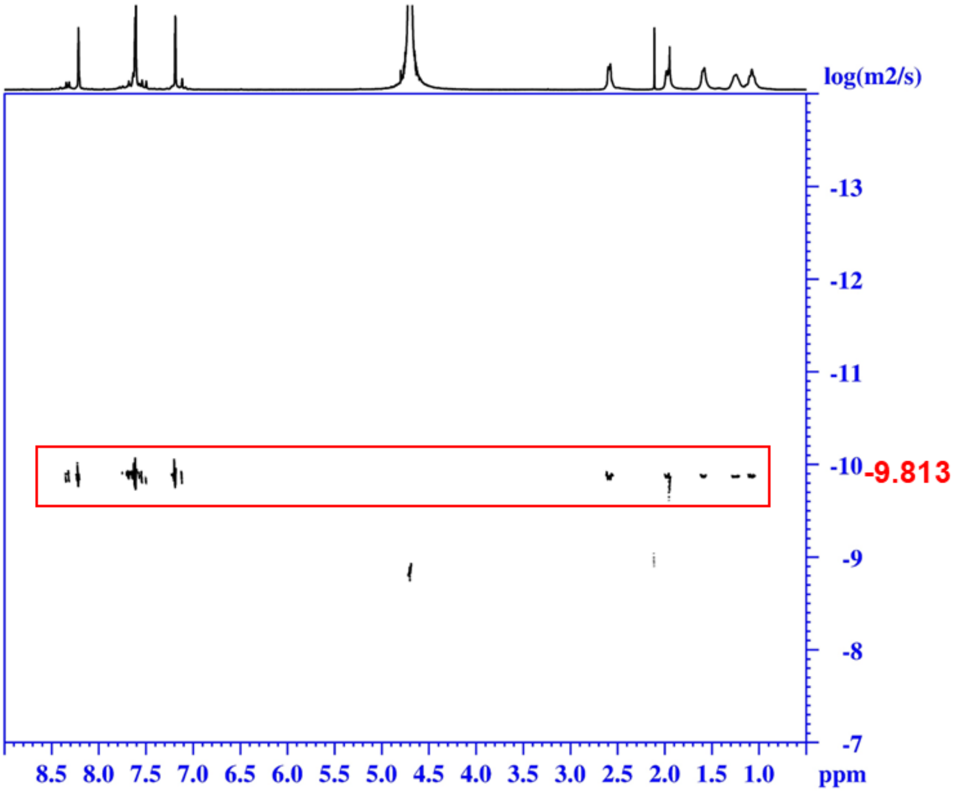

Figure S3. [1]H-DOSY of **C1** in (D_2O, 298 K).

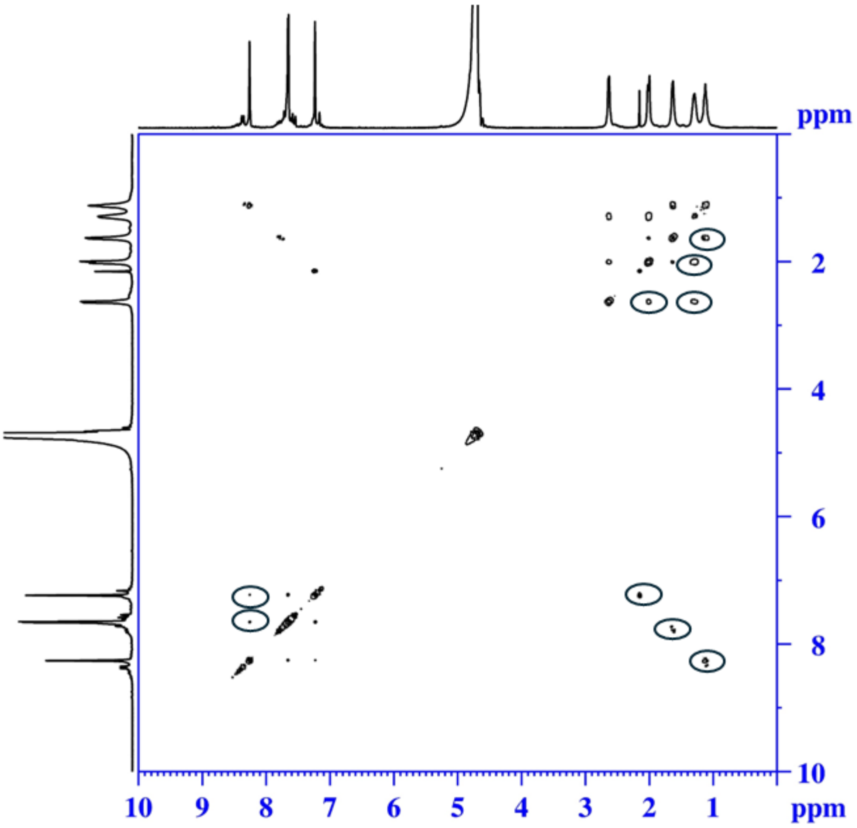

Figure S4. ^{1}H - ^{1}H COSY spectrum of **C1** in (D_2O, 298 K).

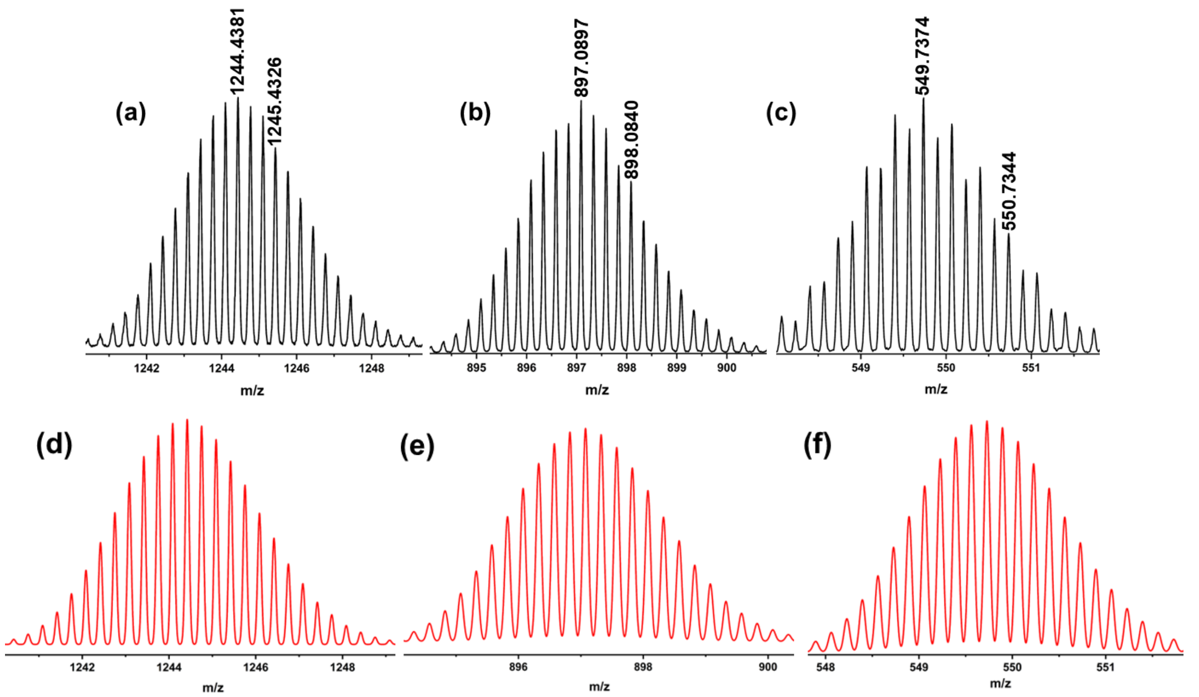

Figure S5. Electrospray ionization mass spectrum of **C_1** (PF_6^-) in acetonitrile. Isotopic distribution patterns:(a) experimental (black) and (d) theoretical (red) of the **[C_1-$3PF_6$]$^{3+}$** fragment; (b) experimental (black) and (e) theoretical (red) of the **[C_1-$4PF_6$]$^{4+}$** fragment; (c) experimental (black) and (f) theoretical (red) isotopic patterns of the **[C_1-$6PF_6$]$^{6+}$** fragment.

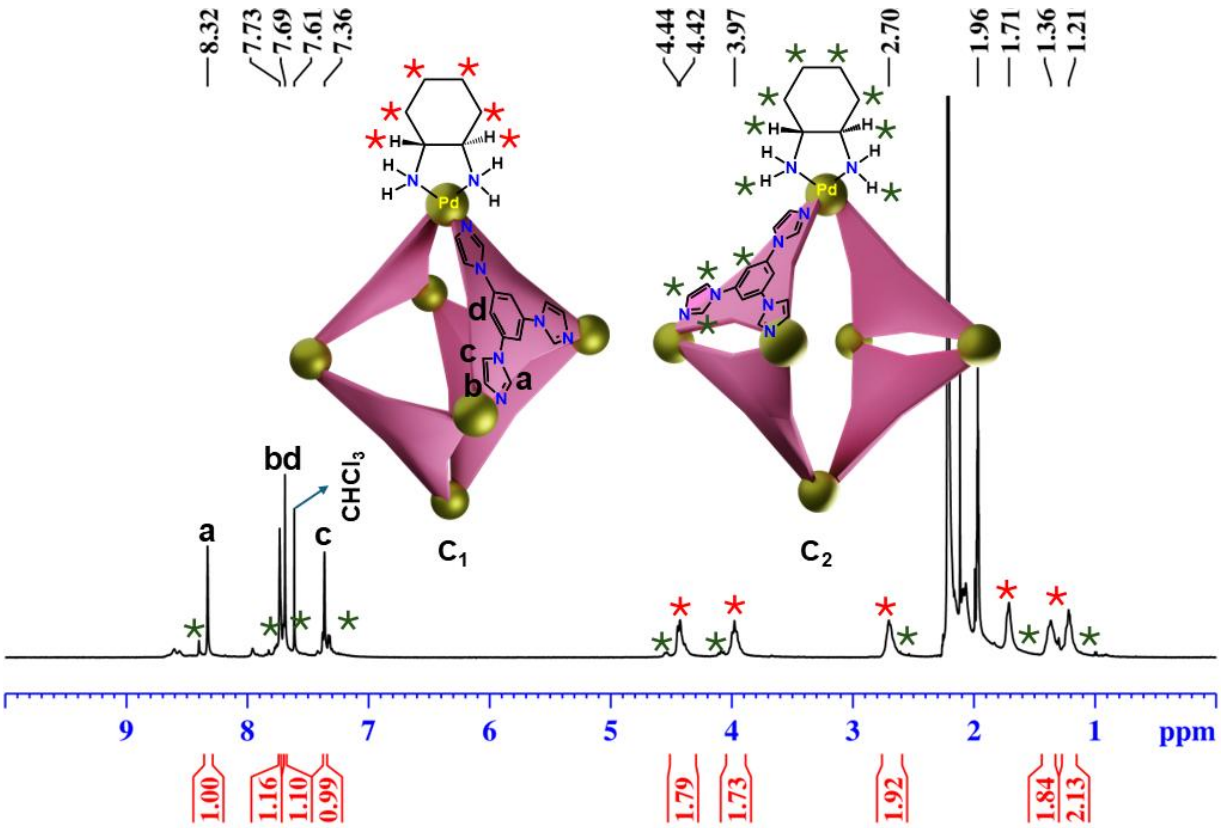

Figure S6. ^{1}H NMR spectra of cages **C_1 (PF_6^-** analogue) (CD_3CN, 298 K).

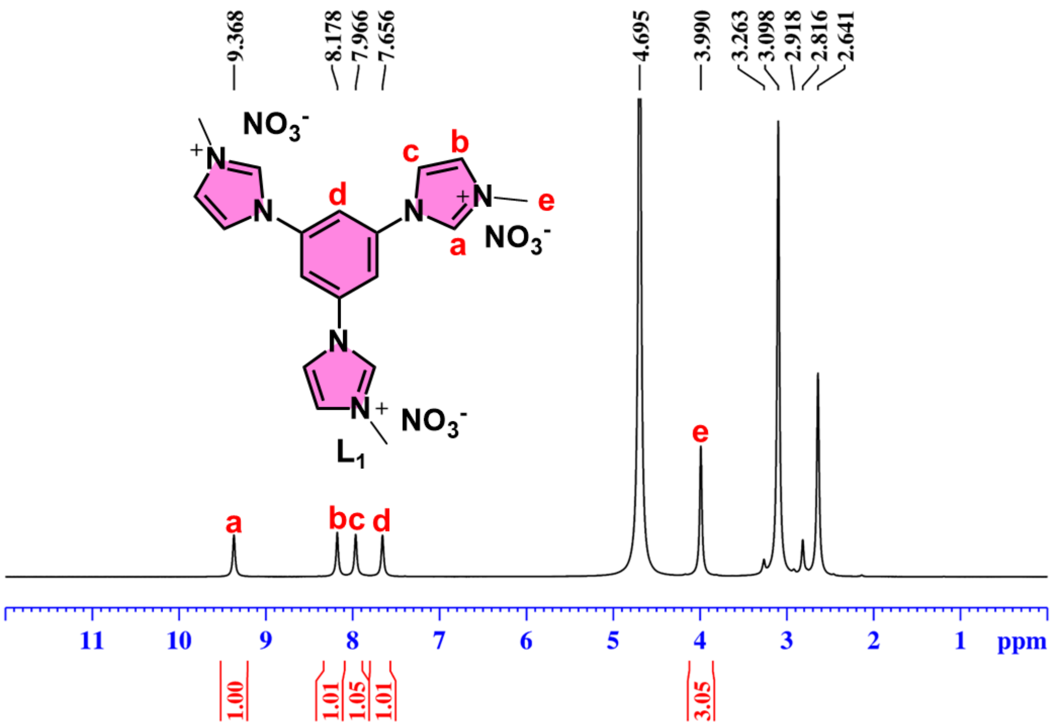

Figure S7. ^{1}H NMR spectra of cages $\mathbf{L_1}(NO_3^-)$ (D_2O, 298 K).

4. X-ray Crystallographic Analysis of $\mathbf{C_1}$

Colourless crystals suitable for single crystal X-Ray diffraction (SCXRD) of $\mathbf{C_1}$ were obtained by slow diffusion of acetone into aqueous solutions of reaction mixtures containing the tri-imidazole donor ligand **L** and the acceptor **M** [M = cis-(1*R*, 2*R*-dch)Pd$(NO_3)_2$] in a 2:3 ratio. SCRXD data were collected with a monochromatic wavelength of 0.7000 Å at the XRD1 beamline of the Elettra synchrotron, Trieste (Italy), employing the rotating-crystal method and a Dectris Pilatus 2M area detector. Measurements were performed at 100(2) K using a nitrogen stream cryo-cooler, with paratone as a cryo-protectant. Diffraction data were indexed, integrated and scaled using the XDS package.[1,2] The structures were solved using the SHELXT package[3] and structure refinement was performed by the full-matrix least-squares (FMLS) method with SHELXL-19/3,[4] operating through ShelXle Qt5 GUI[5]or the WinGX GUI[6]. Hydrogen atoms were included at calculated positions and refined using a riding model. Details of the treatment of non-hydrogen atoms are outlined below. Crystallographic data and refinement details are reported in **Table S1**.

Table S1: Crystallographic data and refinement parameters of $\mathbf{C_1}$

Empirical formula	6 $Pd(C_6H_{14}N_2)^{2+}$, 4 $(C_{15}H_{12}N_6)$, 12 NO_3^-, 0.5 (C_3H_6O), 49.5 H_2O
Formula weight	4093.72
Temperature / K	100(2)
Crystal system	Triclinic
Space group	P1
a, b, c (Å)	21.189(4), 22.633(3), 23.227(4)
α, β, γ (°)	62.872(10), 77.08(3), 69.898(14)
Volume (Å^3)	9280(3)
Z	2
ρ_{calc} (g/cm^3)	1.465
μ (mm^{-1})	0.637
F(000)	4238
Crystal size/mm	0.100 × 0.050 × 0.050
Radiation	synchrotron (λ = 0.700)
2Θ range for data collection (°)	1.946 to 51.924
Index ranges	-26≤ h≤26, -28≤ k ≤28, -329 ≤ l ≤ 29
Reflections collected	181983
Independent reflections	71843 [R_{int} = 0.2196, R_{sigma} = 0.2508]
Data Completeness (%)	98.1
Restraints/parameters	6096/3678
Goodness-of-fit on F^2	0.947
Final R indexes [I>=2σ (I)]	R_1 = 0.0682, wR_2 = 0.1584
Final R indexes [all data]	R_1 = 0.1573, wR_2 = 0.1850
Flack x	0.26(2)
Selected quotients	11064
Parsons z	0.27(3)
Bayesian Statistics	
Bijvoet Pairs	34747
P2(true)	1.000
Hooft y	0.197(18)
CCDC No.	2455229

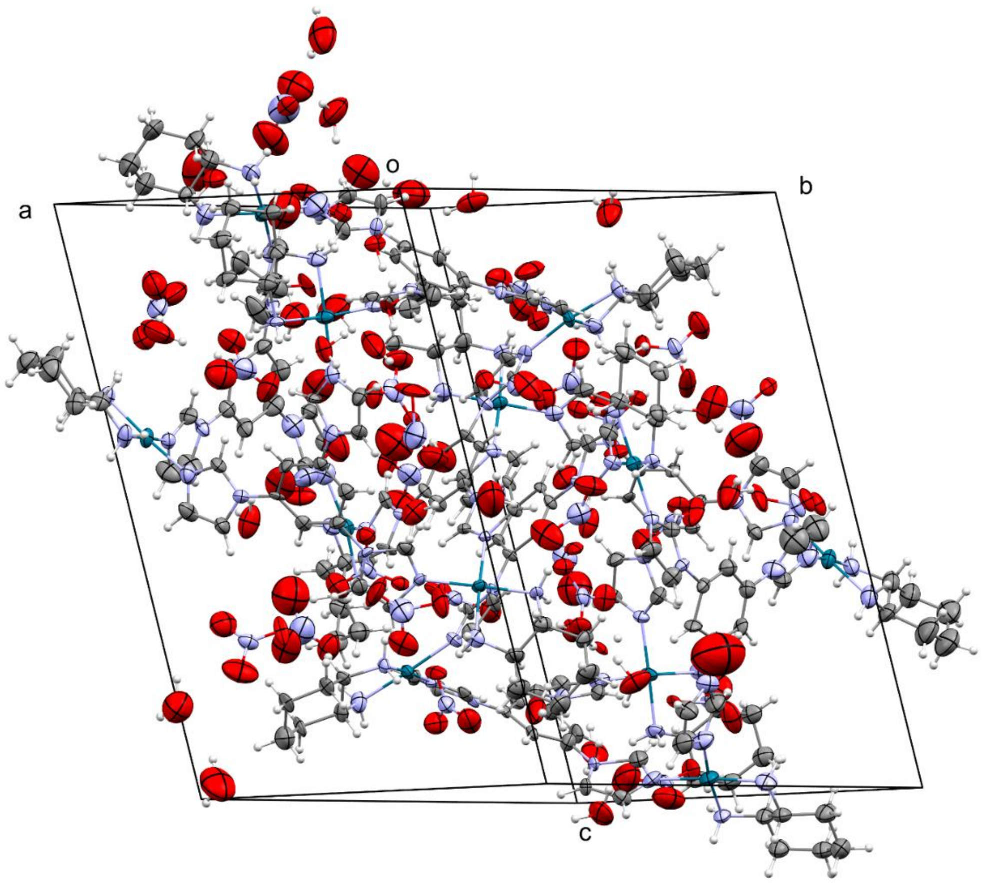

Figure S8. ORTEP diagram of the asymmetric unit / unit cell of **C_1**. Ellipsoids are at 50 % probability. Color codes: carbon (grey), nitrogen (blue), hydrogen (white), oxygen (red) and palladium (green).

Refinement details for C_1

C_1 crystallized in the triclinic P1 space group, and it was modelled with two Pd_6L_4 metallocages, twenty-four nitrate anions, one acetone molecule and a total of thirty water molecules in the asymmetric unit (ASU, **Figure S8**). Each Pd (II) ion in the model has an associated cis-(1*R*, 2*R*-dch) ligand. The Pd(II) ions exhibit a distorted octahedral geometry (vedi infra), with the **L** ligands panelling alternating triangular faces (**Figure S9**).

All non-hydrogen atoms were refined anisotropically. Six sites were refined with nitrate ions at partial occupancy (0.50 occupancy factors in all cases), while three water molecules were refined with two-position disorder (0.60/0.40, 0.60/0.40 and 0.50/0.50 occupancy factors). All other atoms were refined at full occupancy, without disorder, including the cage and the associated pden ligands were refined at full occupancy.

In order to ensure a chemically and geometrically reasonable final model, various restraints and constraints were applied during the final refinement cycles. These include SIMU and ISOR

restraints on all atoms; SAME restraints on all cis-(1R, 2R-dch) ligands; CHIV restraints on the two chiral centres; DFIX restraints on NO bond lengths of one nitrate ion; and 10 EADP constraints on specific pairs of correlated atoms.

The cell also contained residual electron density attributed to water solvent molecules, in addition to the water molecules included in the structure. This residual electron density was not modelled but accounted for using the Platon squeeze tool.[7] The residual electron density of 690 electrons/cell in a total potential solvent accessible void volume of 2078 Å^3 (22.4% of the cell volume) can be attributed to 69 molecules per cell. The adjusted formula was included in the final refinement.

The ASU contains two crystallographically independent molecules which exhibit approximate inversion symmetry. In fact, the structure could also be solved refined in the centrosymmetric space group P-1, yielding similar R-values, albeit with significant correlation issues. However, using this space group would imply that the molecule is present as a racemic mixture. Careful examination of the electron density indicated that P1 is the correct space group, as converting from P-1 to P1 eliminated spurious electron density. At 0.26, the flack parameter is ambiguous in terms of identifying the absolute configuration.[8] However, Bayesian statistical analysis performed with PLATON confirmed the correctness of the assigned absolute structure, with a P2(true) score of 1.000 (**Table S1**).[9]

Geometric details of C$_1$

In each crystallographically independent metallacage of **C$_1$**, the Pd(II) ions occupy the vertices of a distorted octahedron, with **L** ligands covering alternating triangular faces (**Figure S9**). **Table S2** summarises the geometric characteristics of the cages and the arrangement of the **L** ligands with respect to the triangular faces. As implied by the above-mentioned approximate inversion relationship between the two cages the two independent cages are similar, however, each is asymmetric and all edge lengths of the triangular faces are different (**Table S2**).

As indicated in **Figure S9** and **Table S2**, three of the **L** ligands are considerably twisted and ill-fitting with respect to the faces they occupy. The centroids of their central benzene rings lie outside the polyhedron, and the mean planes of the imidazole rings are orientated with large dihedral angles with respect to the cage faces. However, in each cage, one of the four faces covered by **L** is larger than the other three, with a higher area (**Table S2**). In these two cases,

L is less strained. It is slightly concave with respect to face formed by the three Pd^{2+} ions, the corresponding dihedral angles made by the mean planes of the imidazole are smaller and the plane of its central aromatic 6-membered ring are closer to parallel with this face. In the crystal structure, the cages are present as dimers in which these larger faces aligned almost parallel to each other (0.44°), but with a relative rotation of 60 ° (**Figure S10**). This 60 ° rotation allows mutual intersection of the cis-(1R, 2R-dch) ligands with the other cages and formation of π ··· π interactions between the two central aromatic 6-membered rings of the dimer. The mean planes of the central aromatic 6-membered rings form an angle of 1.88°, with a distance between the centroids of the two benzene rings distance of 3.52 Å.
Overall, the dimeric structure of $\mathbf{C_1}$ is similar to that of our previously reported self-assembled structure obtained using the same tritopic donor **L** ligand and an analogous non-chiral *cis*-blocked acceptor: 2,2-dimethylpropane-1,3-diamine (pden).[10] However, in the present case the symmetry is lower

The crystal packing is characterised by significant interstitial void space between dimers when viewed along the *b* and *c* axes (**Figure S11**). These are filled by water molecules and nitrate anions. This figure also highlights that the distorted nature of the octahedral cage, with quite large openings to allow access inside the cages. In fact, three of the unoccupied faces have a larger surgace area tan any of the covered faces. The unoccupied triangular faces have the following characteristics:

Cage 1: (1) sides = 9.27 x 9.18 x 9.02 Å, area = 36.29 Å^2; (2) sides = 11.49 x 11.36 x 12.82 Å, area = 60.62 Å^2; (3) sides = 11.53 x 11.37 x 12.83 Å, area = 60.84 Å^2;(4) sides = 11.61 x 11.47 x 12.81 Å, area = 60.48 Å^2.

Cage2: (1) sides = 9.27 x 9.01 x 8.99 Å, area = 35.76 Å^2; (2) sides = 11.50 x 11.43 x 12.82 Å, area = 60.93 Å^2; (3) sides = 11.57 x 11.48 x 12.80 Å, area = 61.34 Å^2; (4) sides = 11.60 x 11.27 x 12.86 Å, area = 60.78 Å^2.

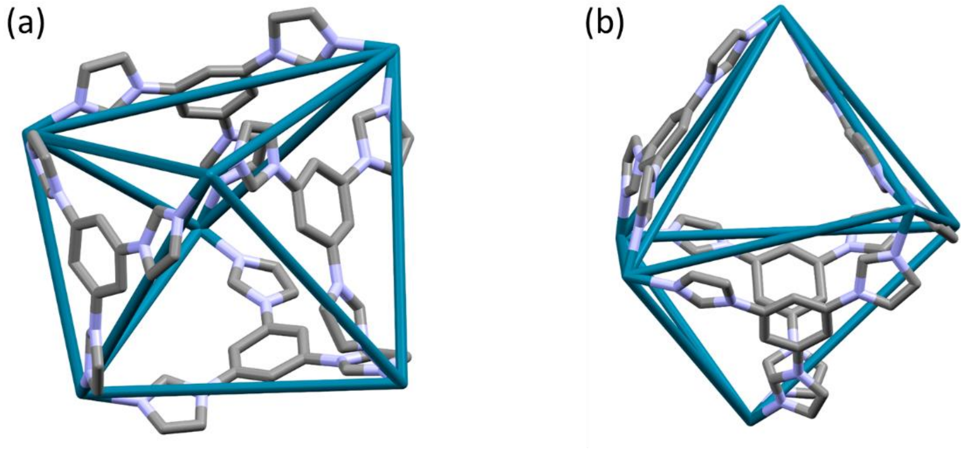

Figure S9: Illustration of the distorted octahedral cage structures formed by the Pd^{2+} ions of $\mathbf{C_1}$ and panelling arrangements of the **L** ligands on the triangular faces: (a) top view and (b) side view of one crystallographically independent cage. The second independent cage exhibits an almost identical structure. Color codes: carbon (grey), nitrogen (blue), and palladium (green). Nitrate anions, water molecules, cis-(1*R*, 2*R*-dch) ligands and hydrogen atoms have been omitted for clarity.

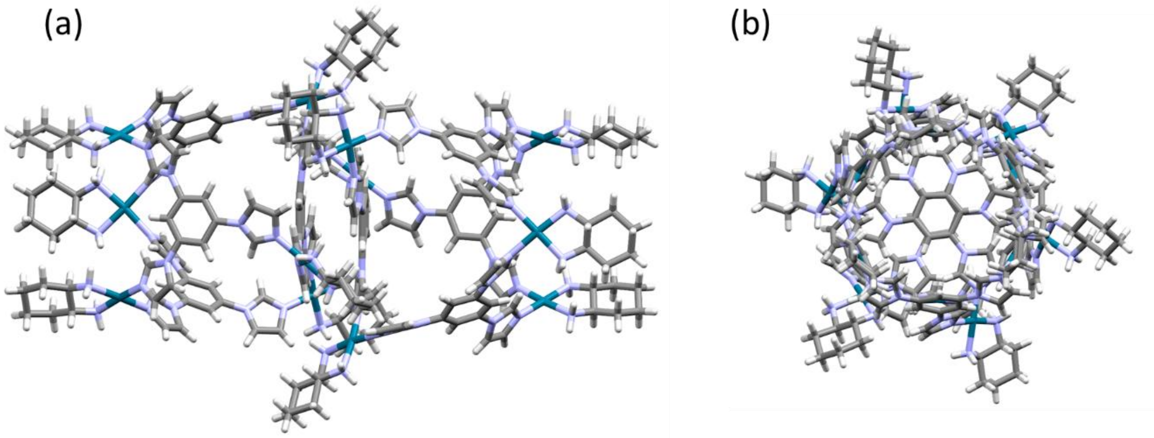

Figure S10: Dimeric species formed by the two crystallographically independent cages of $\mathbf{C_1}$. Color codes: carbon (grey), nitrogen (blue), hydrogen (white) and palladium (green). Nitrate anions and water molecules have been omitted for clarity.

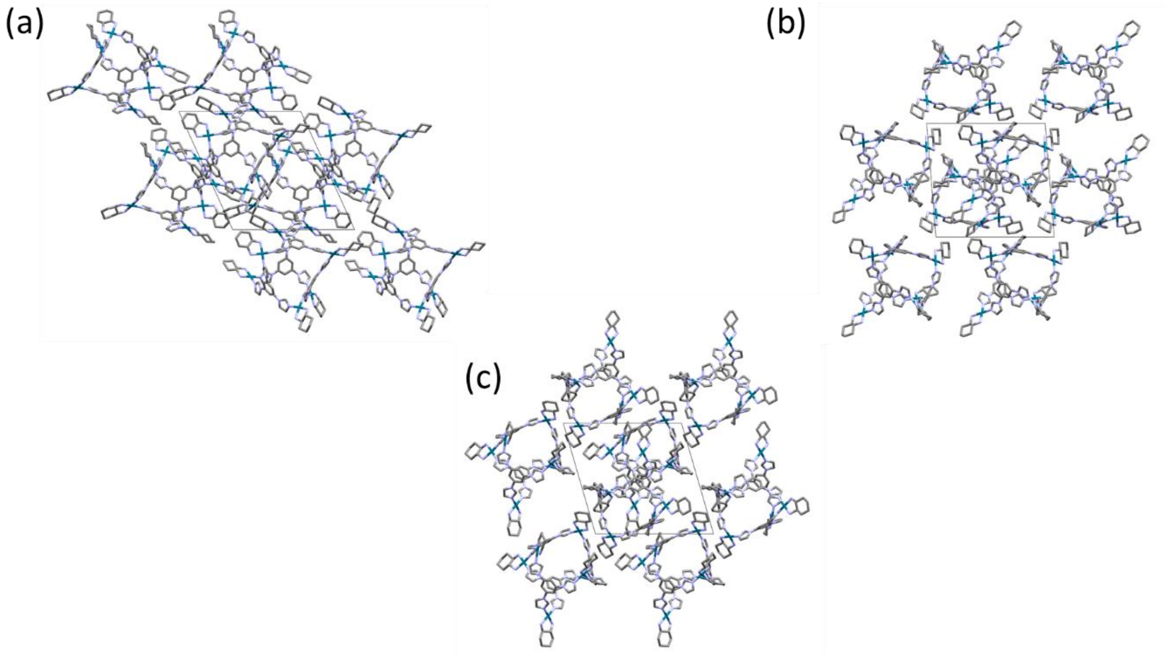

Figure S11: Crystal packing of the cages of $\mathbf{C_1}$, as viewed along the *a*-axis (a), *b*-axis and (b) and *c*-axis (c). Color codes: carbon (grey), nitrogen (blue) and palladium (green). Nitrate anions, water molecules and hydrogen atoms have been omitted for clarity

Table S2: Geometric Parameters Summarising the fit of the **L** Ligands on the Faces of the **C_1** cages

Face Sides[a] (Å)	Area[b] (Å^3)	d face-L[c] (Å)	Dihedral angle: benzene of L-cage face [d] (°)	Dihedral angle: imidazole of L – cage face[e] (°)
		**Cage 1**		
11.49	57.70	- 0.58	2.46	10.78
11.53				10.60
11.61				9.07
9.27	51.13	1.14	2.55	23.03
11.37				22.09
12.81				27.07
9.18	51.05	1.04	4.20	16.31
11.47				29.18
12.82				33.25
9.02	49.94	1.15	7.02	21.63
11.36				26.53
12.83				24.56
		**Cage 2**		
11.50	57.83	- 0.58	0.80	12.47
11.57				12.01
11.60				12.78
9.27	51.31	1.20	4.74	22.69
11.43				31.23
12.80				21.44
9.01	50.33	1.08	2.27	27.28
11.48				16.98
12.86				33.11
8.99	49.48	1.21	3.69	28.35
11.27				21.69
12.82				30.26

[a] lengths of the triangular cage faces; [b] Area of cage face; [c] Distance between the cage face and the centroid of the central 6-membered ring of **L**. Negative values indicate that the centroid lies inside the polyhedron and positive values indicate that it lies outside the polyhedron; [d] Dihedral angle between the plane of the cage face and the mean plane of the central 6-membered ring of **L**; [e] Dihedral angles between the plane of the cage face and the mean planes of the imidazole rings of **L**.

5. Host-Guest Encapsulation Study:

An excess amount of solid Thieno[3,2-*b*]thiophene (**G$_1$**), Naphthalene (**G$_2$**), phenacyl bromide (**G$_3$**) and Ibuprofen (**G$_4$**) was separately added to an aqueous solution of **C$_1$**. The mixtures were stirred at ambient temperature for 2 hours, resulting in the formation of cloudy suspensions. These mixtures were then centrifuged, and the clear supernatants were collected for NMR analysis.

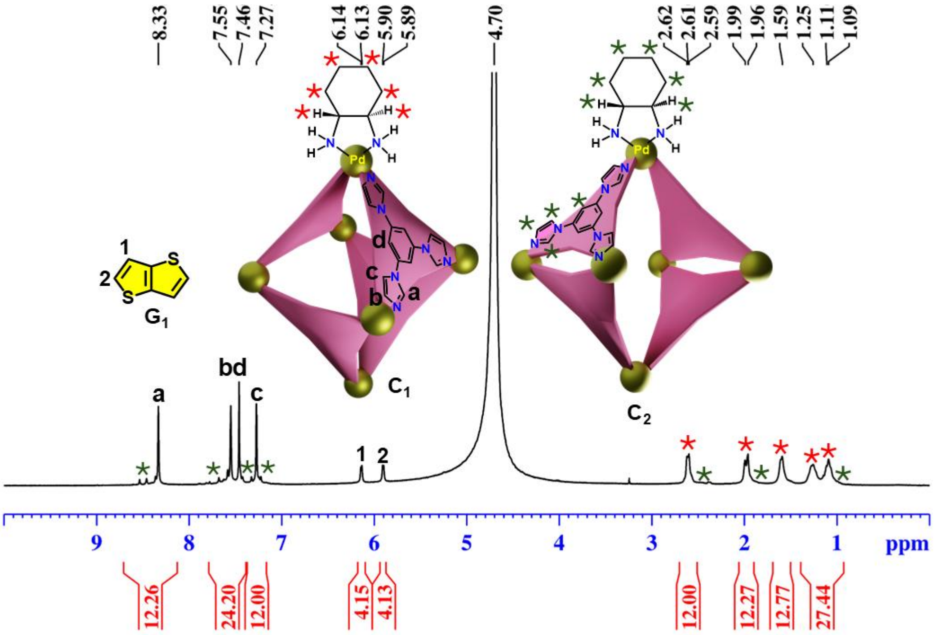

Figure S12. ^{1}H NMR spectra of cages **C$_1$** + **G$_1$** (D_2O, 298 K).

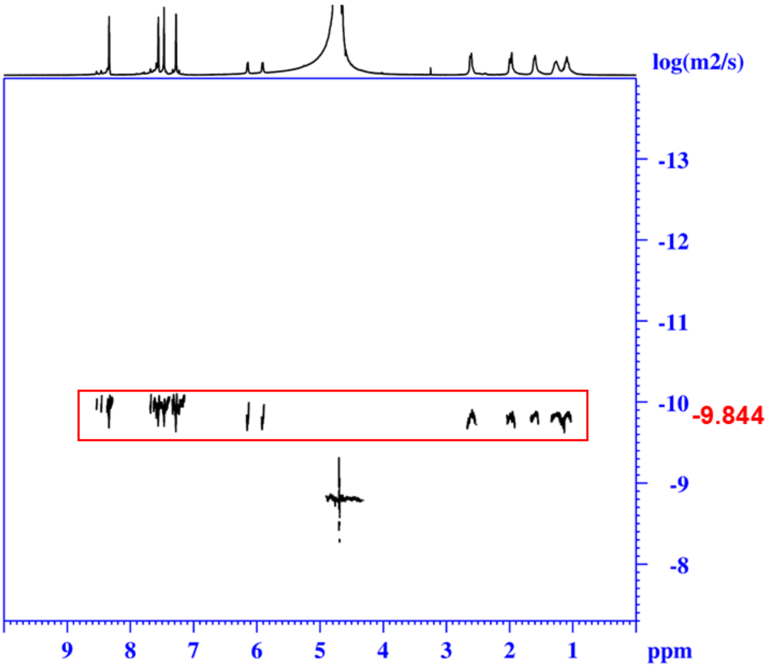

Figure S13. ^{1}H DOSY NMR spectra of cages **C$_1$** + **G$_1$** (D_2O, 298 K).

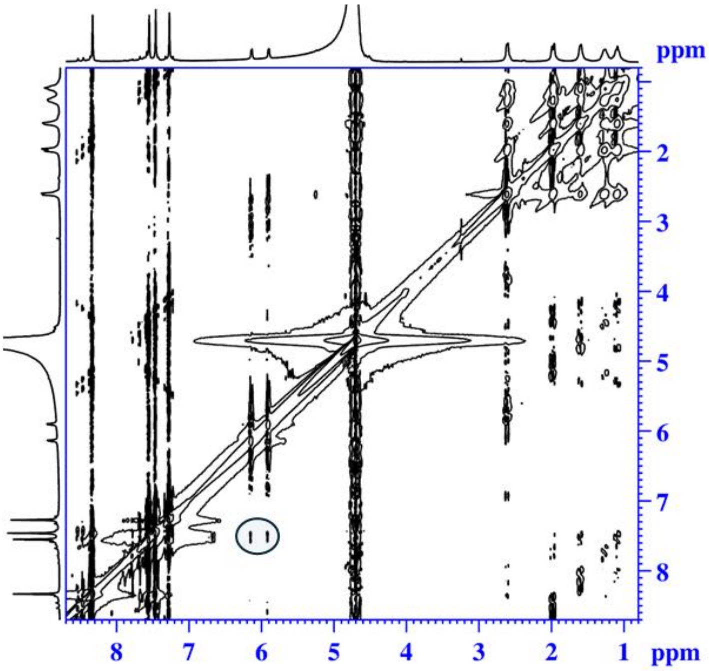

Figure S14. ^{1}H - ^{1}H NOESY spectrum of $\mathbf{C_1} + \mathbf{G}_1$ in (D_2O, 298 K).

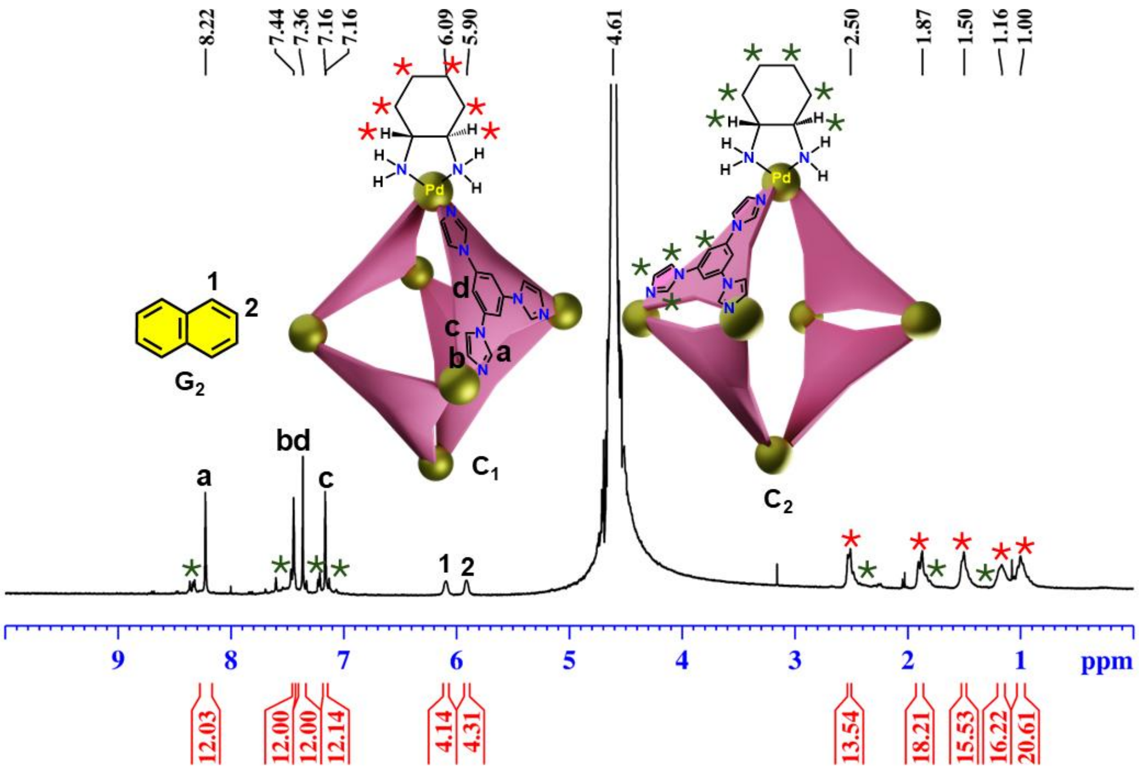

Figure S15. ^{1}H NMR spectra of cages $\mathbf{C_1} + \mathbf{G}_2$ (D_2O, 298 K).

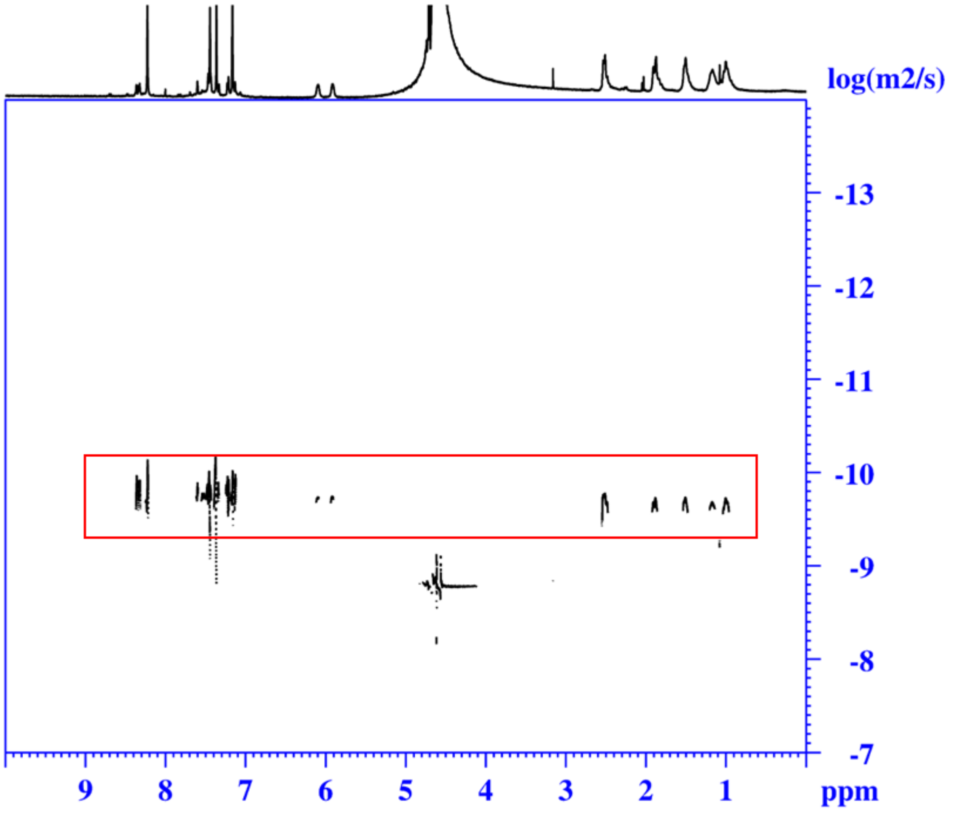

Figure S16. DOSY NMR spectra of cages $\mathbf{C_1}$ + $\mathbf{G_2}$ (D_2O, 298 K).

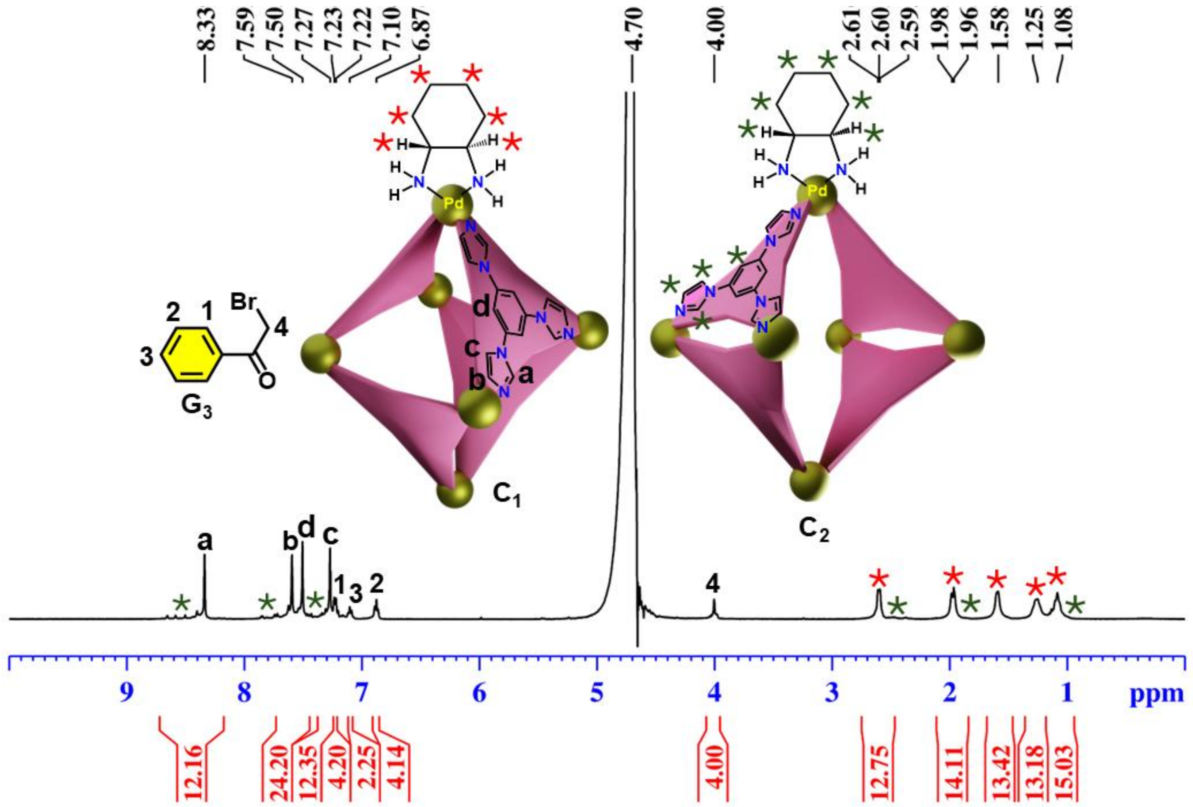

Figure S17. ^{1}H NMR spectra of cages $\mathbf{C_1}$ + $\mathbf{G_3}$ (D_2O, 298 K).

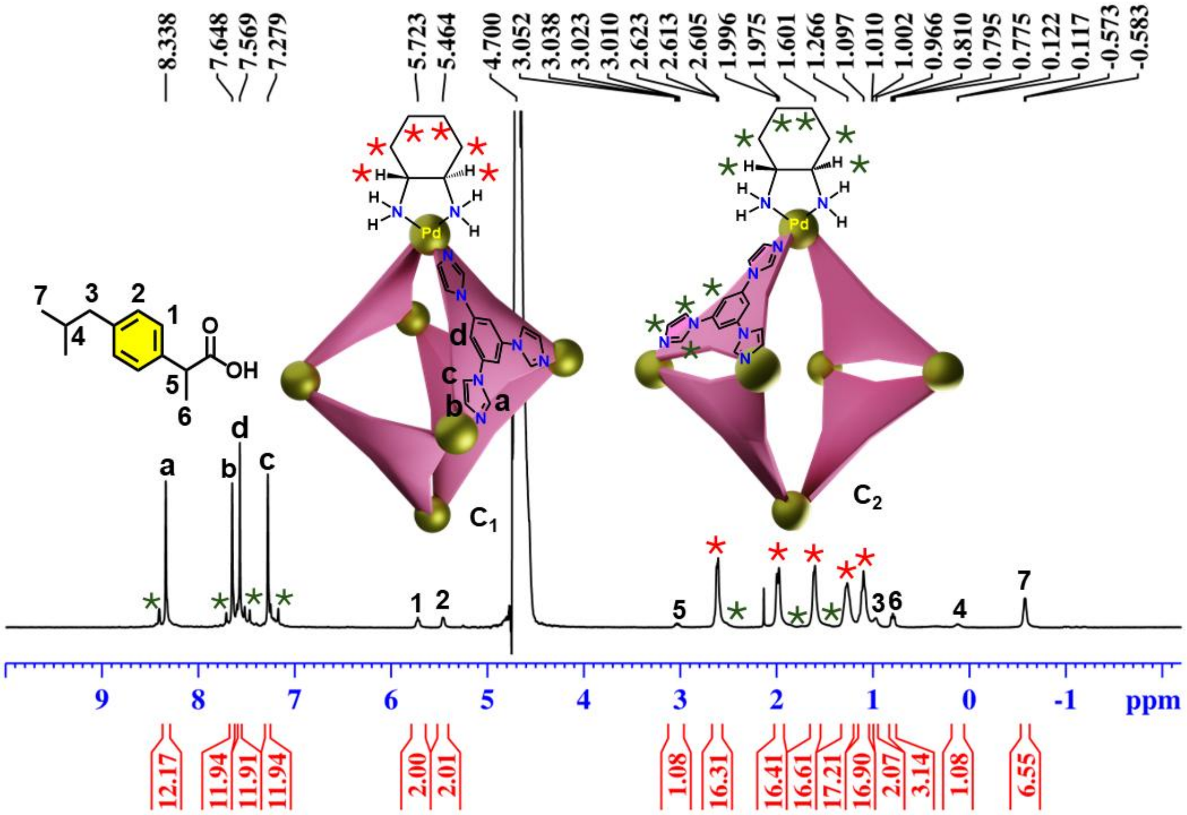

Figure S18. ^{1}H NMR spectra of cages **C₁** + **G₄** (D_2O, 298 K).

5.1. Host–Guest Encapsulation Study of Terminal Alkynes:

An excess amount of liquid ethynyl benzene (**R₁**), 1-ethynyl-4-methylbenzene (**R₂**), 1-ethynyl-3-methylbenzene (**R₃**), 1-ethynyl-4-methoxybenzene (**R₄**), 2-ethynylthiophene (**R₅**) and 1,3-diethynylbenzene (**R₆**) was separately added to an aqueous solution of **C₁**. The mixtures were stirred at ambient temperature for 2 hours. These mixtures were then centrifuged, and the clear supernatants were collected for further analysis.

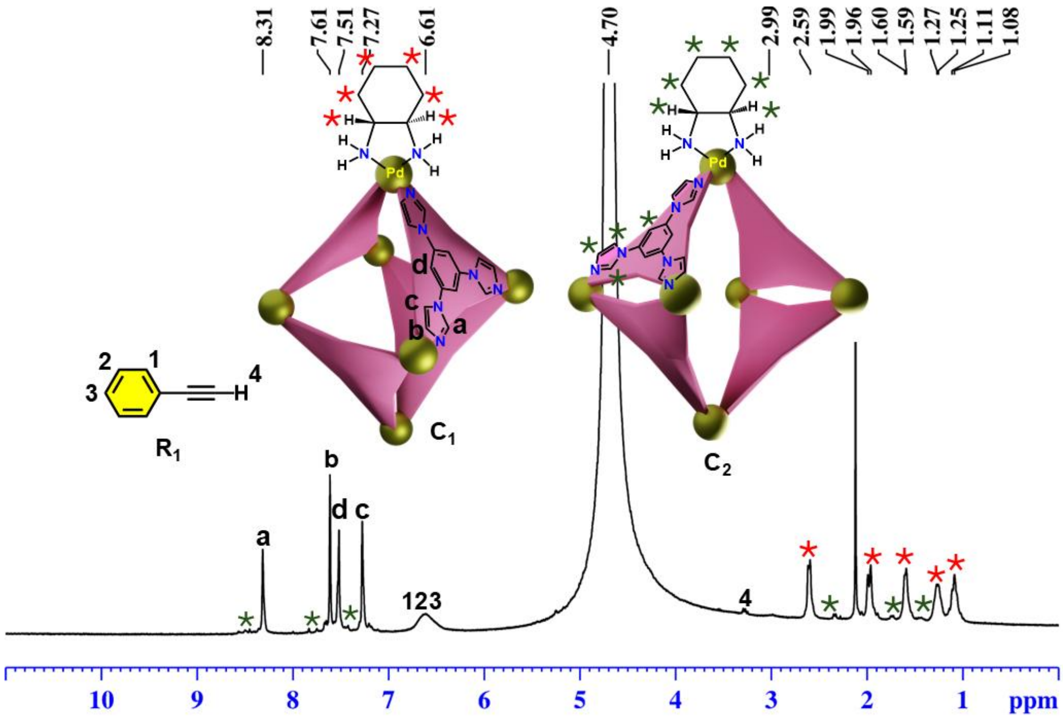

Figure S19. ^{1}H NMR spectrum of cages **C1** + **R1** (D_2O, 298 K).

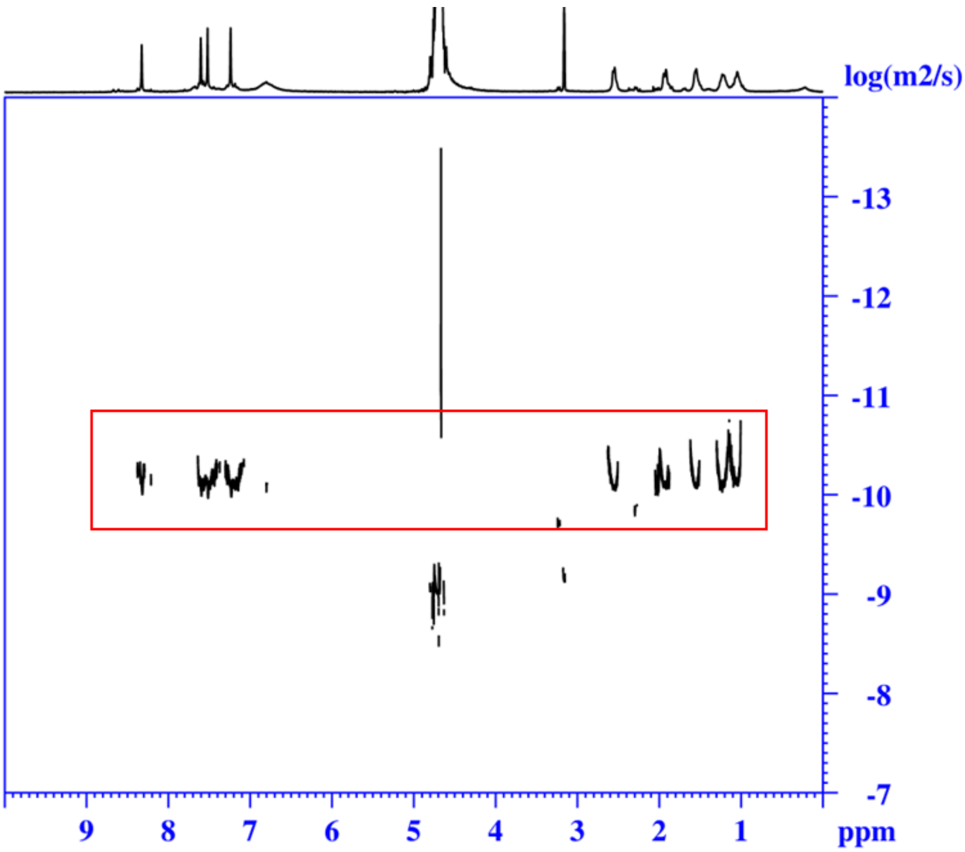

Figure S20. DOSY NMR spectra of cages **C1** + **R1** (D_2O, 298 K).

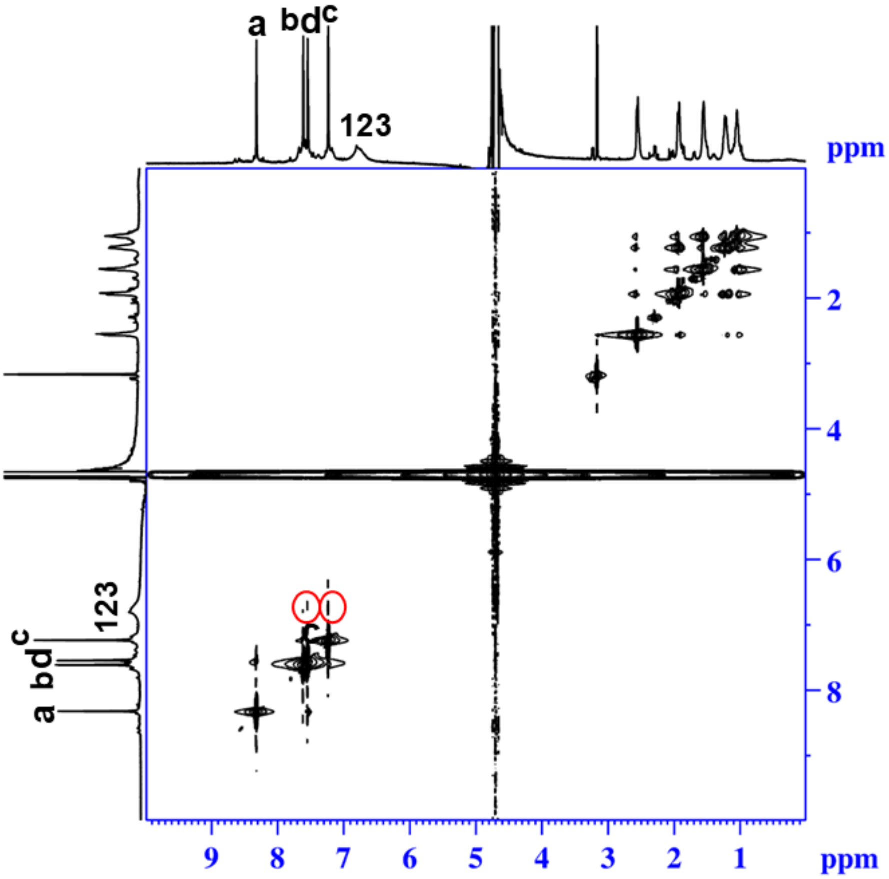

Figure S21: ^{1}H - ^{1}H NOESY spectrum of $\mathbf{C_1} + \mathbf{R_1}$ in (D_2O, 298 K).

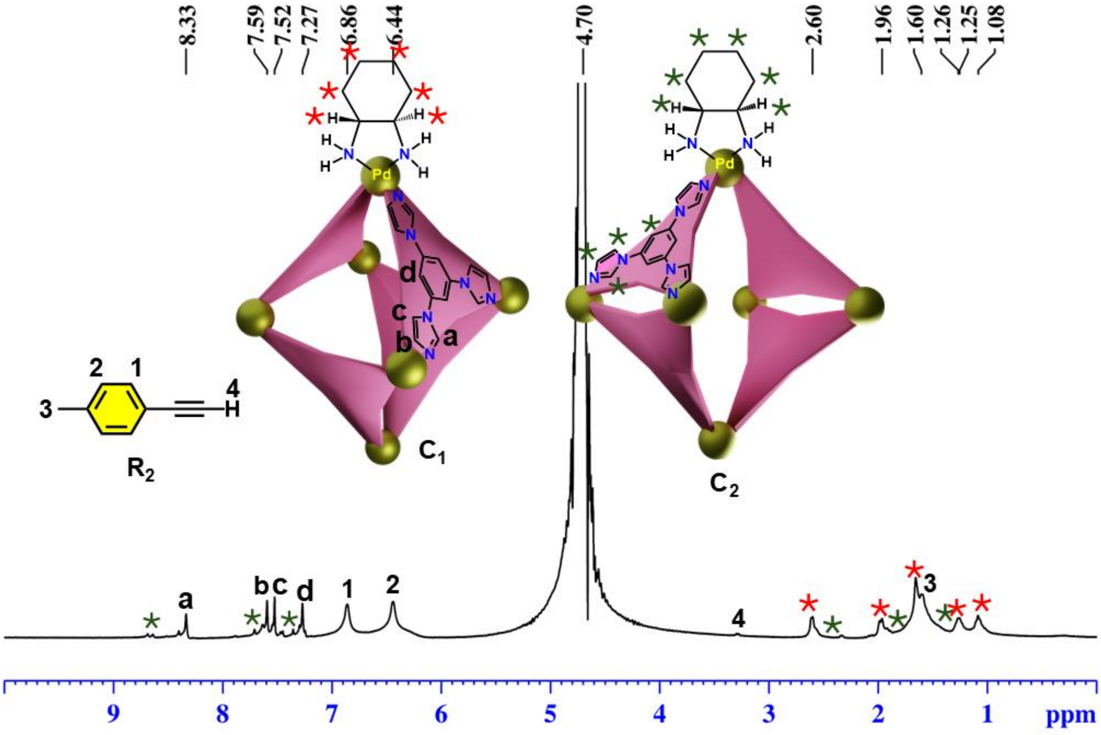

Figure S22. ^{1}H NMR spectra of cages $\mathbf{C_1} + \mathbf{R_2}$ (D_2O, 298 K).

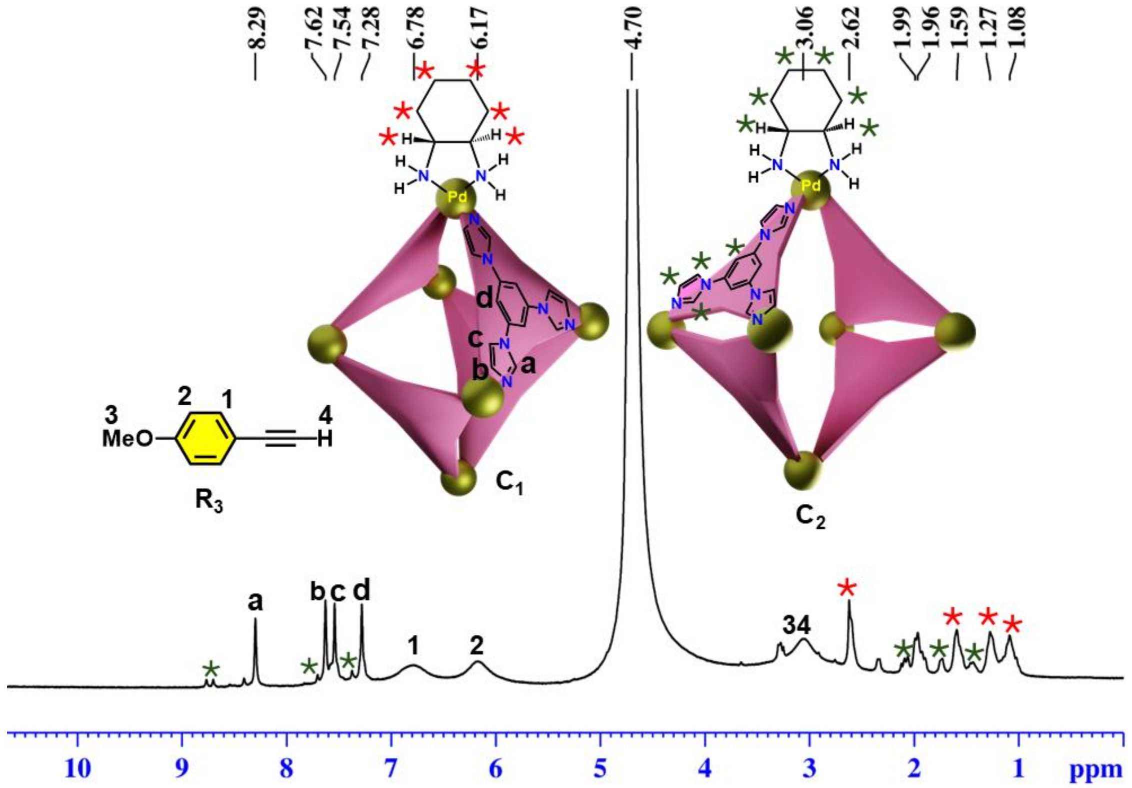

Figure S23. ^{1}H NMR spectra of cages $\mathbf{C_1}$ + $\mathbf{R_3}$ (D_2O, 298 K).

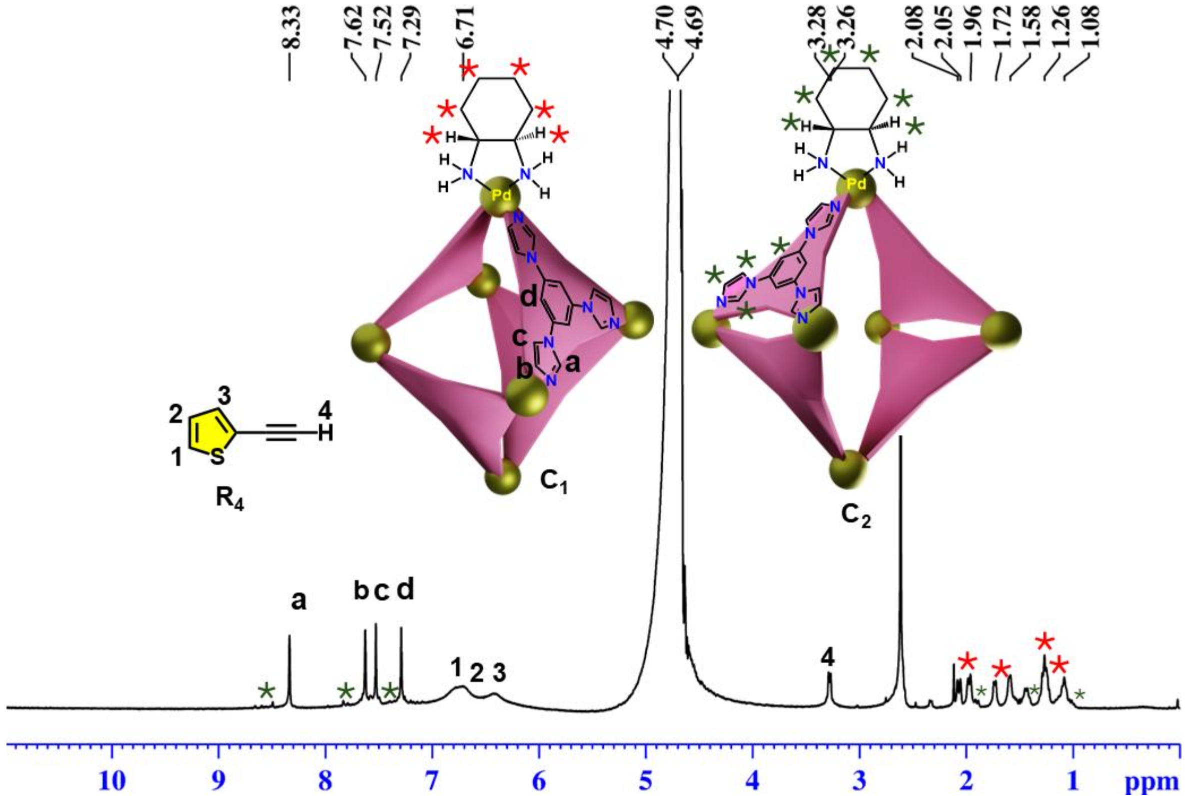

Figure S24. ^{1}H NMR spectra of cages $\mathbf{C_1}$ + $\mathbf{R_4}$ (D_2O, 298 K).

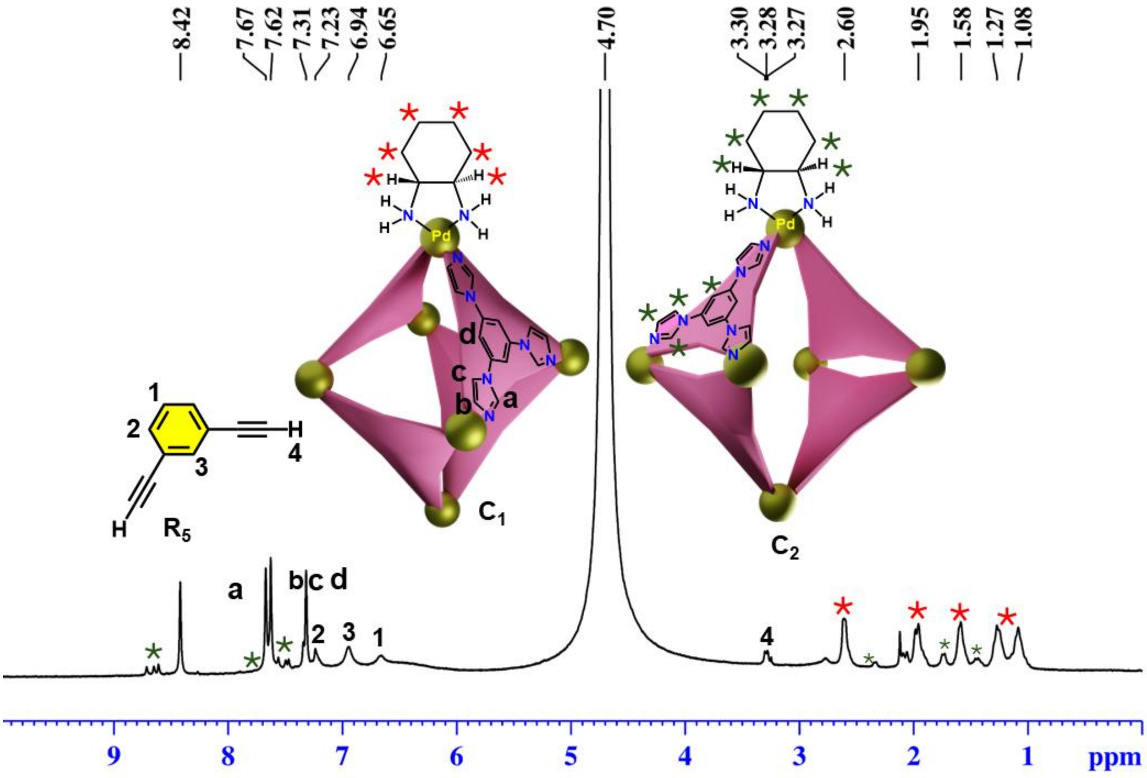

Figure S25. ^{1}H NMR spectra of cages $\mathbf{C_1} + \mathbf{R_5}$ (D_2O, 298 K).

5.2. NMR Titrations of $\mathbf{R_1}$ with $\mathbf{C_1}$:

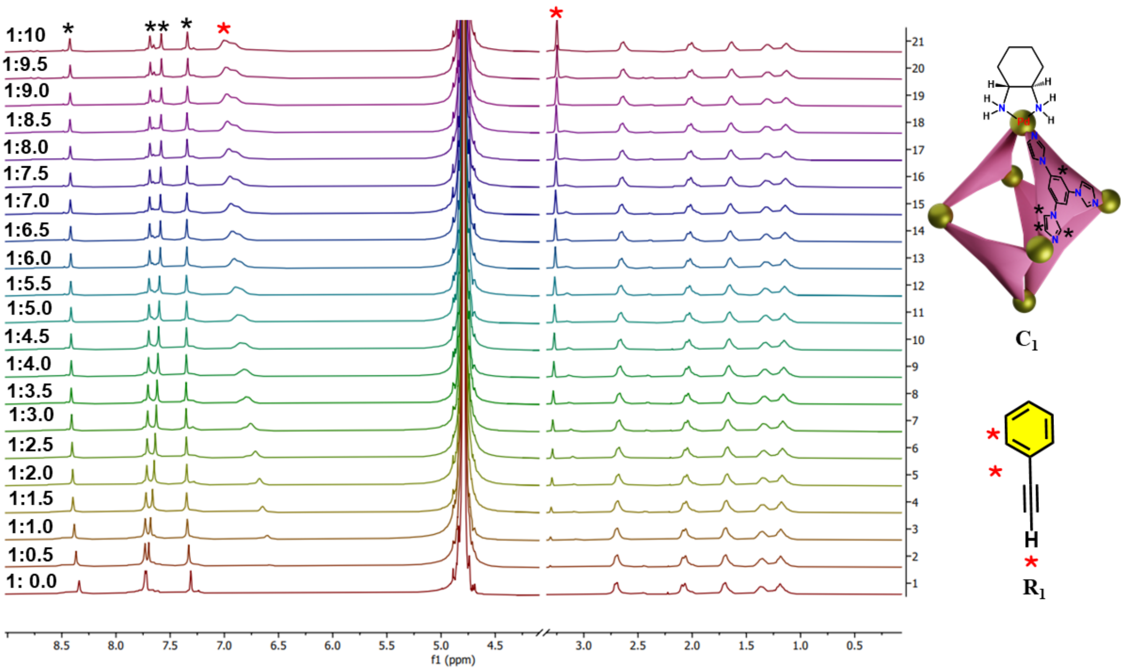

Figure S26a. ^{1}H NMR titration plot for $\mathbf{R_1 \subset C_1}$ cage host-guest complexes at different guest concentrations. showing changes in chemical shifts of protons from benzene triimidazole unit.

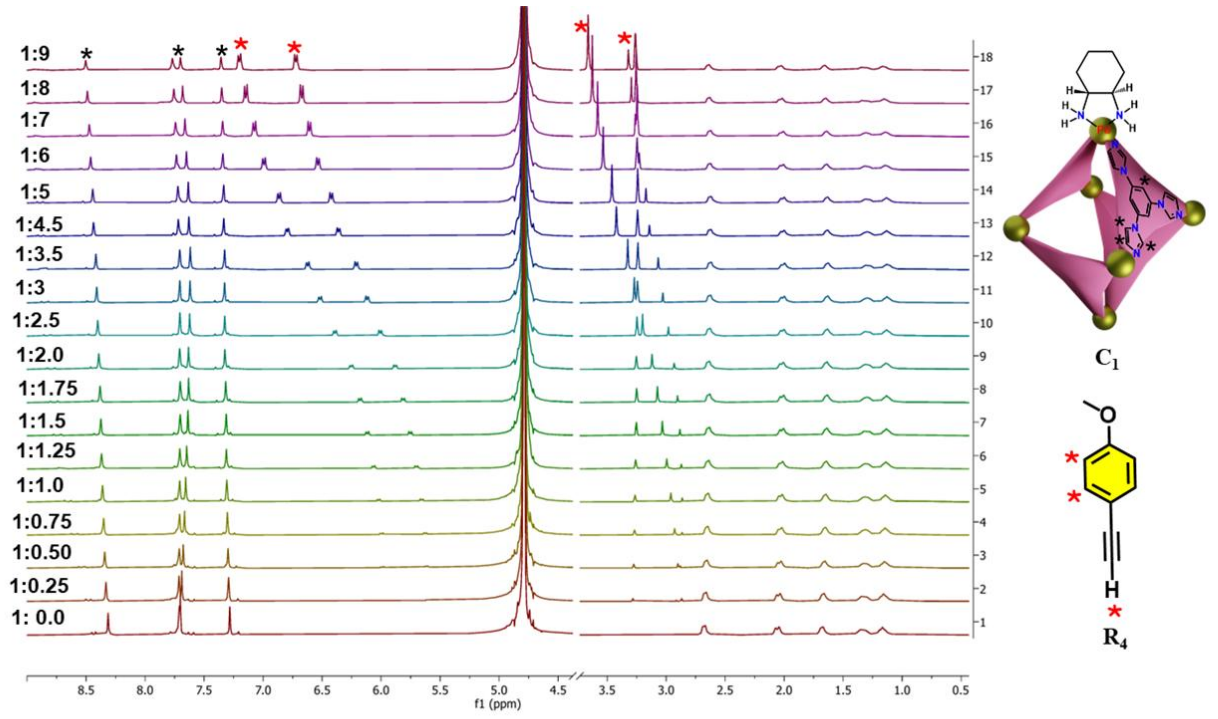

Figure S26b. ^{1}H NMR titration plot for **$R_4 \subset C_1$** cage host-guest complexes at different guest concentrations. showing changes in chemical shifts of protons from benzene triimidazole unit.

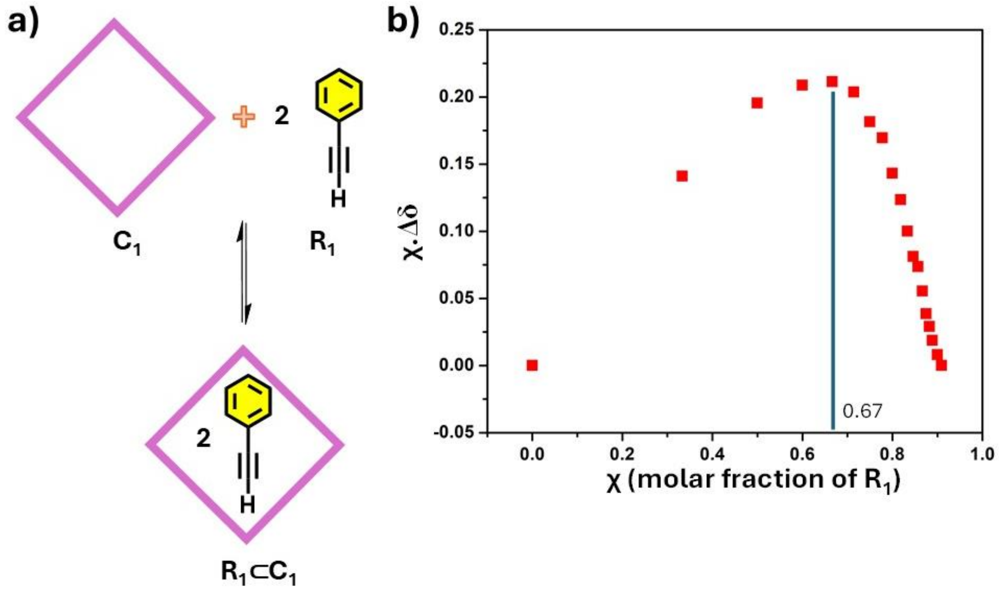

Figure S27a. Job's plots for **$R_1 \subset C_1$** cage host-guest complexes.

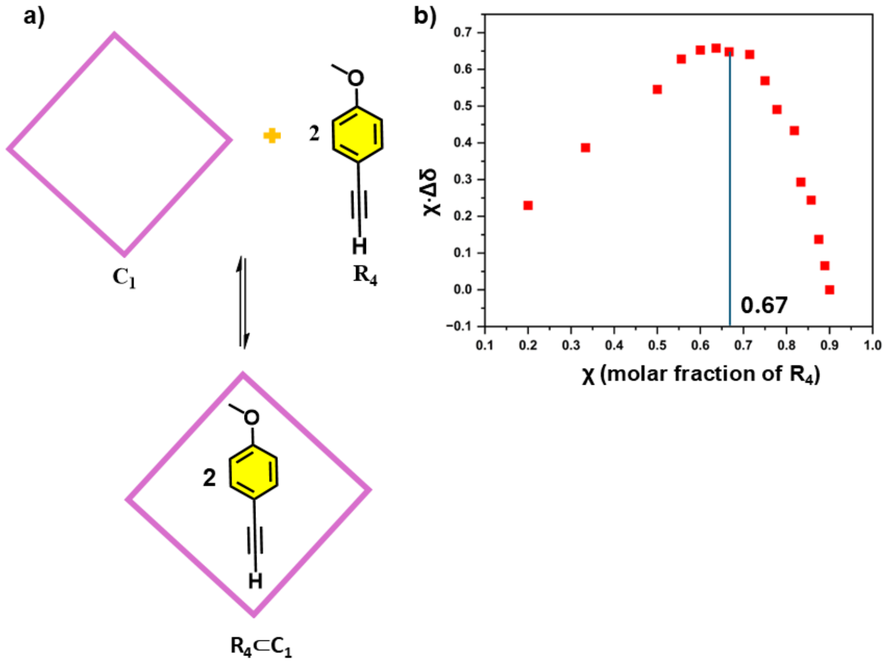

Figure S27b. Job's plots for $\mathbf{R_4 \subset C_1}$ cage host-guest complexes.

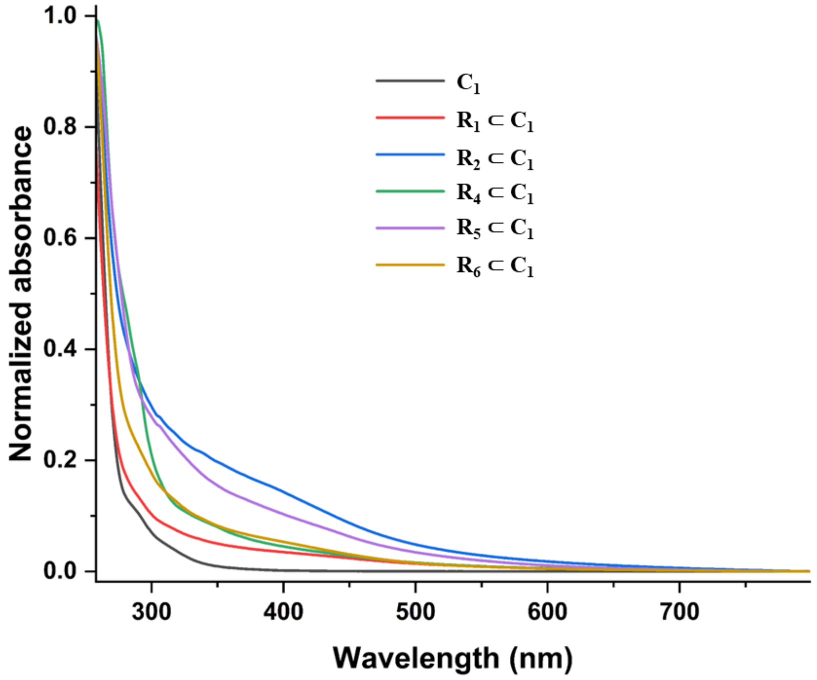

Figure S28a. Normalized absorption spectra of ethynyl derivatives inside $\mathbf{C_1}$ cage in water (10^{-5}M). (A 10^{-3} M solution of $\mathbf{C_1}$ was first prepared in water, after which the terminal alkyne was added. Upon stirring for 5 mins, the mixture was then centrifuged to remove any undissolved material, and the aqueous supernatant was diluted to 1×10^{-5} M for UV–vis analysis).

.

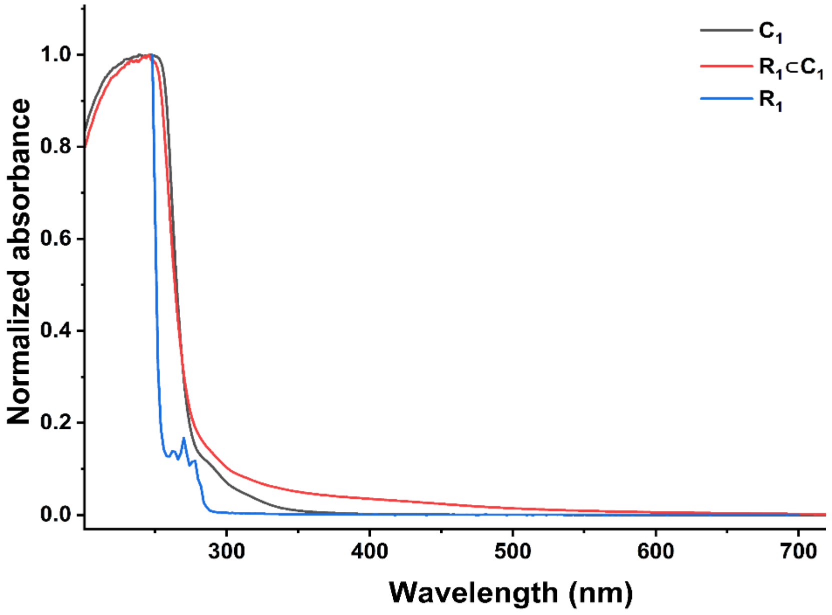

Figure S28b. Normalized absorption spectra of **C**$_1$ cage (water), phenyl acetylene inside **C**$_1$ cage **R**$_1$⊂**C**$_1$ (water), phenyl acetylene **R**$_1$ (Methanol). C= 10^{-5}M.

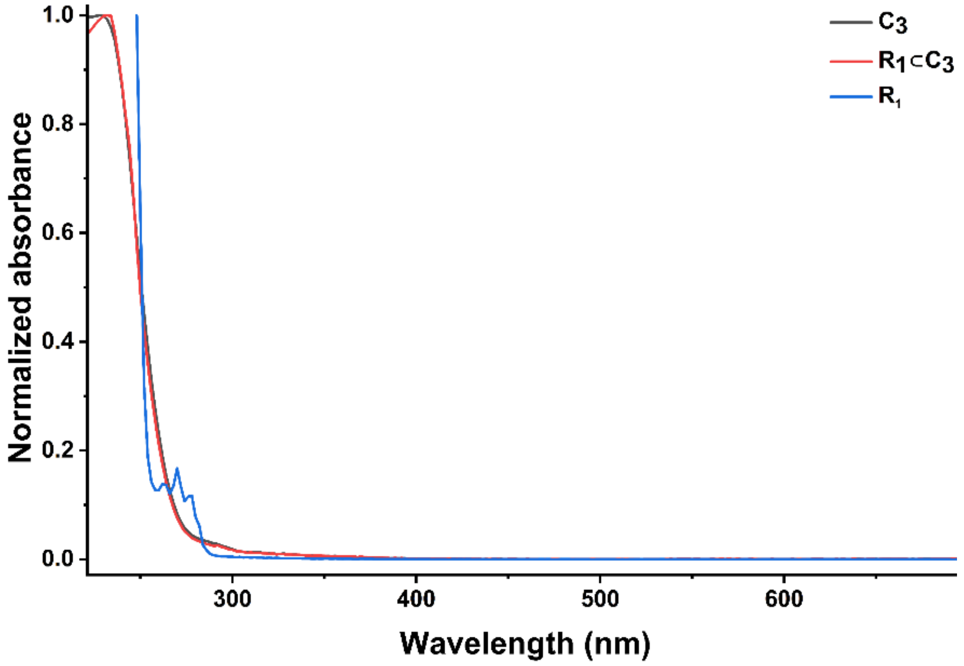

Figure S28c. Normalized absorption spectra of **C**$_3$ cage (water), phenyl acetylene inside **C**$_3$ cage **R**$_1$⊂**C**$_3$ (water), phenyl acetylene **R**$_1$ (Methanol). C = 10^{-5}M).

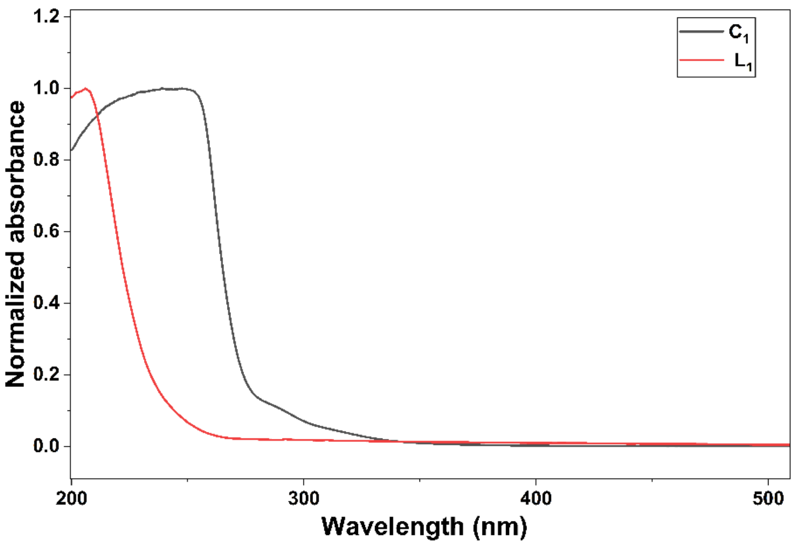

Figure S28d. Normalized absorption spectra of **C1** cage and methylated imidazolium ligand (**L1**) in water (10^{-5}M).

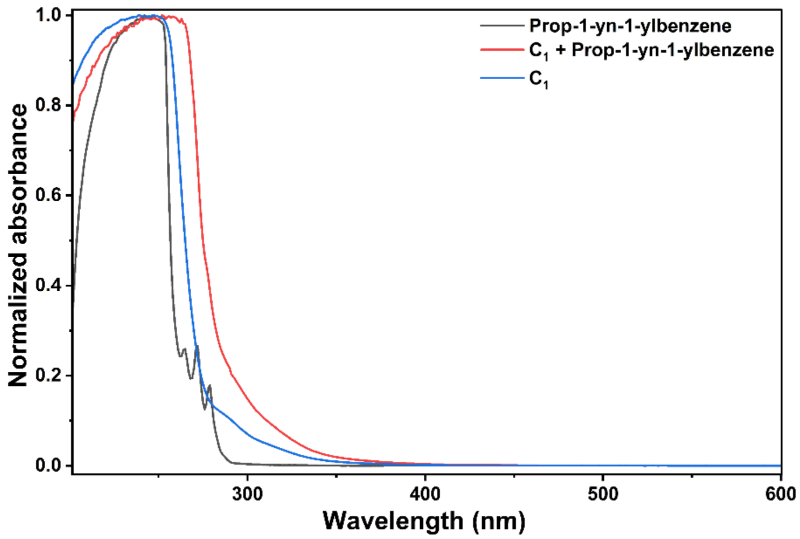

Figure S28e. Normalized absorption spectra of **C1** cage (water), prop-1-yn-1-ylbenzene inside **C1** cage (water), prop-1-yn-1-ylbenzene (Methanol).

6. General Procedure for Photocatalysis

The photoreactions were carried out under ambient conditions. The aqueous solution of **C_1** (5 mol %) was taken a Schlenk tube, and the substrate was added into the tube. Then the solution mixture was irradiated with blue LED (390 nm, 100 W) for 2 h under room temperature. To maintain the temperature, a cooling fan has been near to the reaction flask (Figure S22). After completion of the reaction, the aqueous solution was extracted with EtOAc. The extracted product was characterized by ^{1}H NMR spectroscopy and GC-MS.

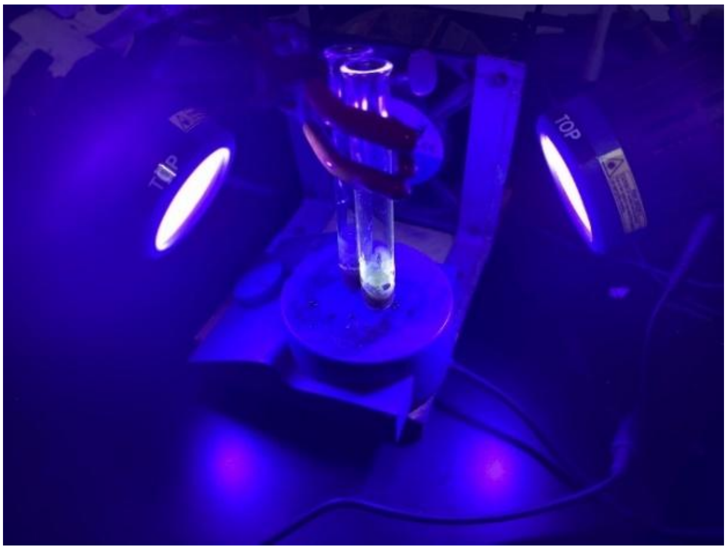

Figure S29. Experimental setup for photocatalysis.

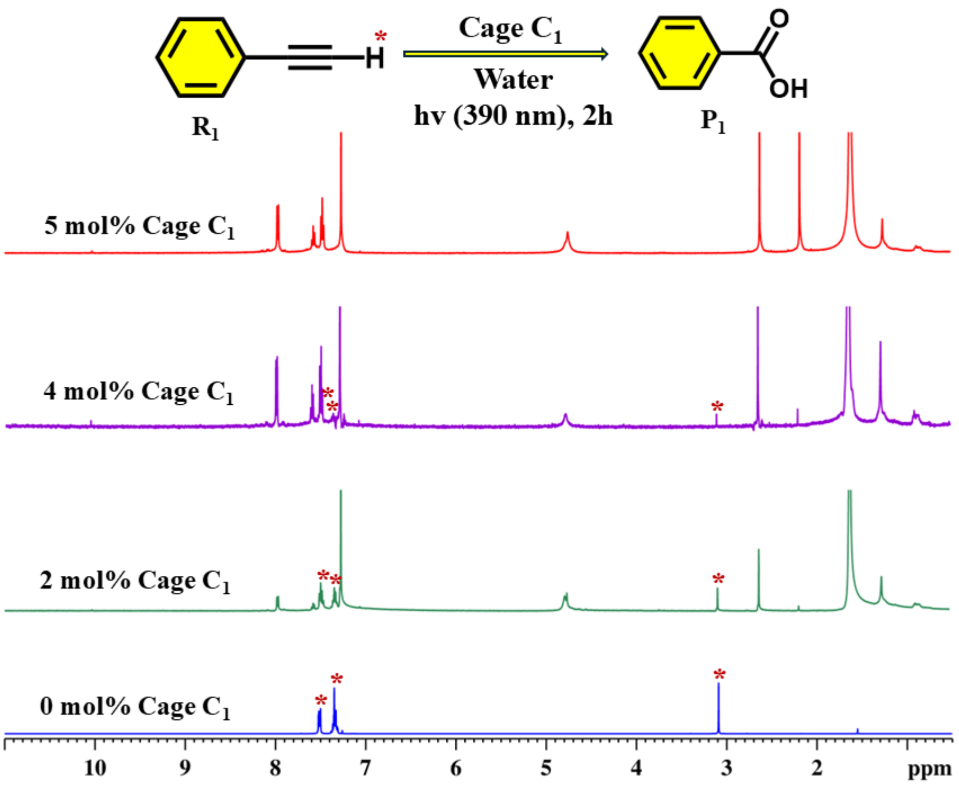

Figure S30. Stacked ^{1}H NMR spectra for **P_1** synthesis at different cage mol% ($CDCl_3$, 298 K). The ^{1}H NMR spectra were recorded from the crude reaction mixtures after direct extraction with $CDCl_3$, as benzoic acid is highly soluble in $CDCl_3$.

6.1 After each reaction, samples were extracted using ethyl acetate, dried over anhydrous Na_2SO_4, and then evaporated before NMR analysis.

P₁ →

P_1 (^1H NMR ($CDCl_3$, 400 MHz): δ (ppm) = 8.13 (d, 2H), 7.64 (m, 1H), 7.50 (m, 2H). The spectral data for this compound match those reported in the literature.[11]

P_2 (^1H NMR ($CDCl_3$, 400 MHz): δ (ppm) = 8.02 (d, 2H), 7.28 (d, 2H), 2.43 (s, 3H). The spectral data for this compound match those reported in the literature.[11]

P_3 (^1H NMR ($CDCl_3$, 400 MHz): δ (ppm) = 7.93 (s, 1H), 7.92 (s, 1H), 7.43 (d, 1H), 7.38 (t, 1H), 2.42 (s, 3H). The spectral data for this compound match those reported in the literature.[11]

P_4 (^1H NMR ($CDCl_3$, 400 MHz): δ (ppm) = 8.08 (d, 2H), 6.96 (d, 2H), 3.88 (s, 3H). The spectral data for this compound match those reported in the literature.[11]

P_5 (^1H NMR ($CDCl_3$, 400 MHz): δ (ppm) = 7.90 (d, 1H), 7.66 (d, 1H), 7.15 (m, 1H). The spectral data for this compound match those reported in the literature.[12]

P_6 (^1H NMR (DMSO-d_6, 400 MHz): δ (ppm) = 13.27 (s, 2H), 8.48 (s, 1H), 8.18 (d, 2H), 7.66 (m, 1H).[13]

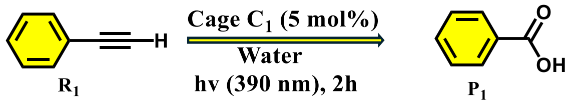

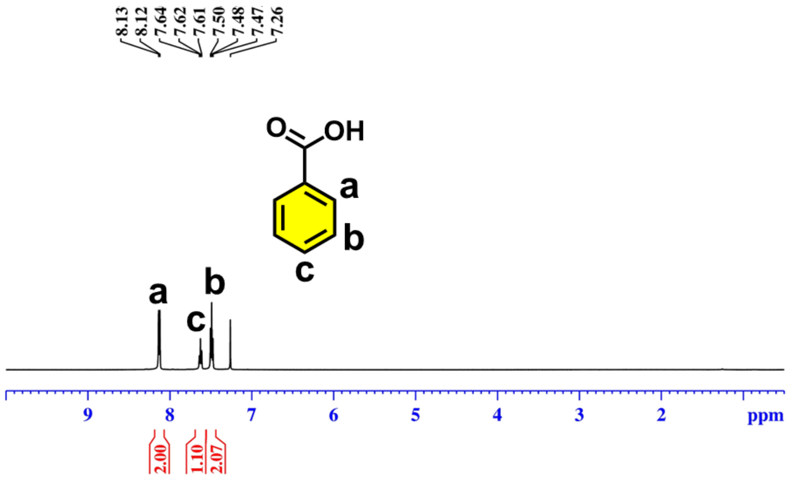

Figure S31. The ^1H NMR spectrum of the irradiation of **R_1** ($CDCl_3$, 298 K).

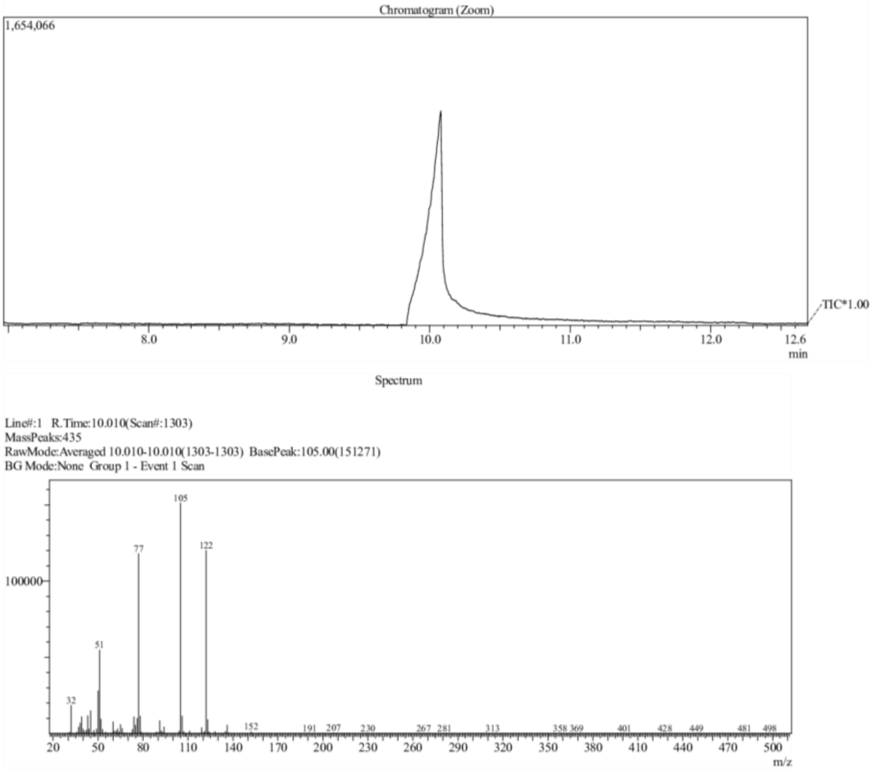

Figure S32. GCMS data in presence of ethynylbenzene (**R**$_1$) to benzoic acid (**P**$_1$).

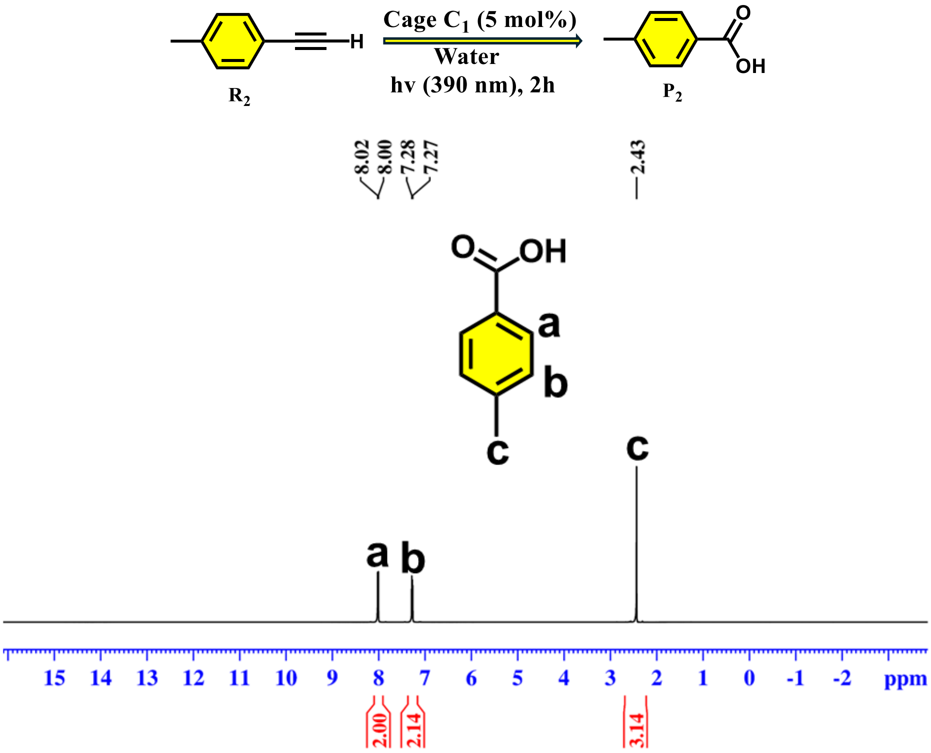

Figure S33. The ^{1}H NMR spectrum of the irradiation of **R**$_2$ (CDCl$_3$, 298 K).

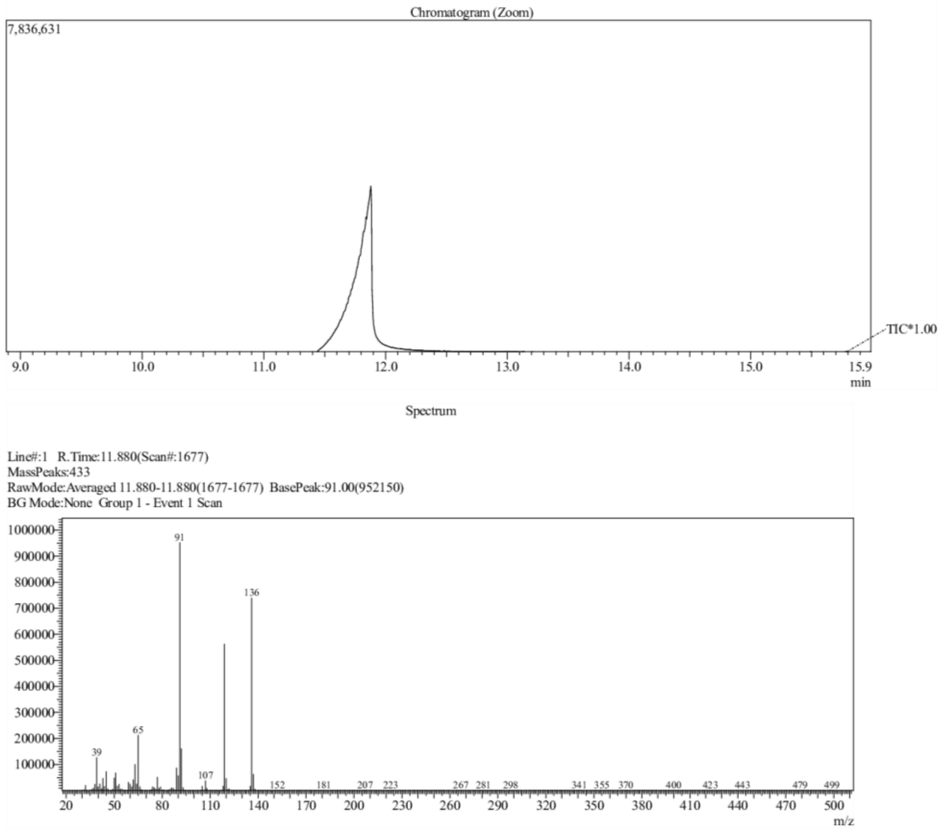

Figure S34. GCMS data in presence of 1-ethynyl-4-methylbenzene (**R$_2$**) to 4-methylbenzoic acid (**P$_2$**).

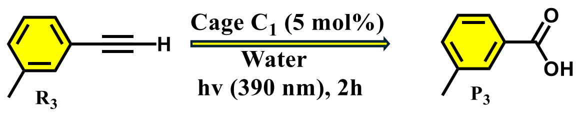

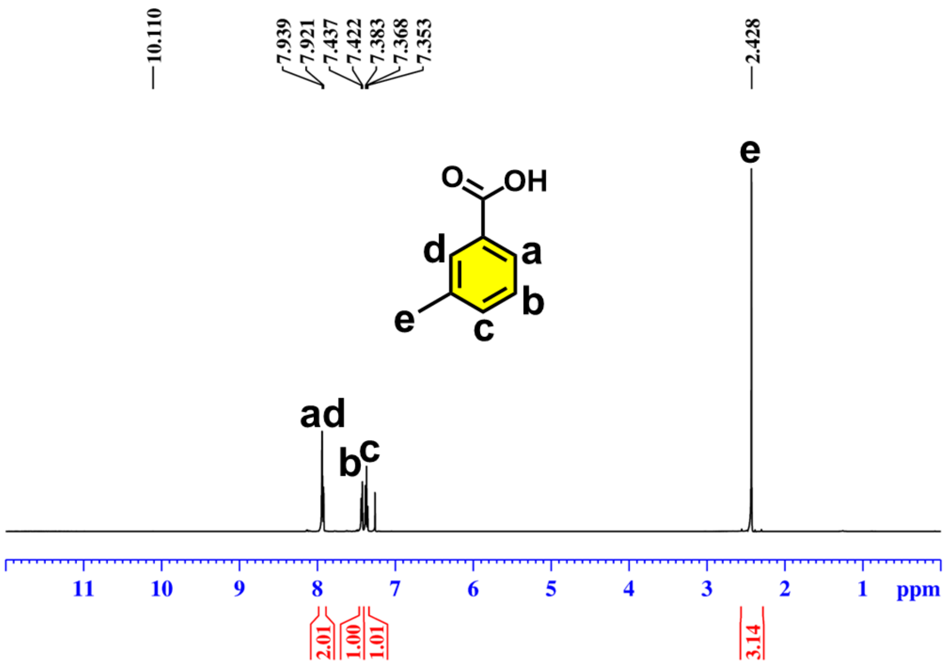

Figure S35. The ^{1}H NMR spectrum of the irradiation of **R$_3$** (CDCl$_3$, 298 K).

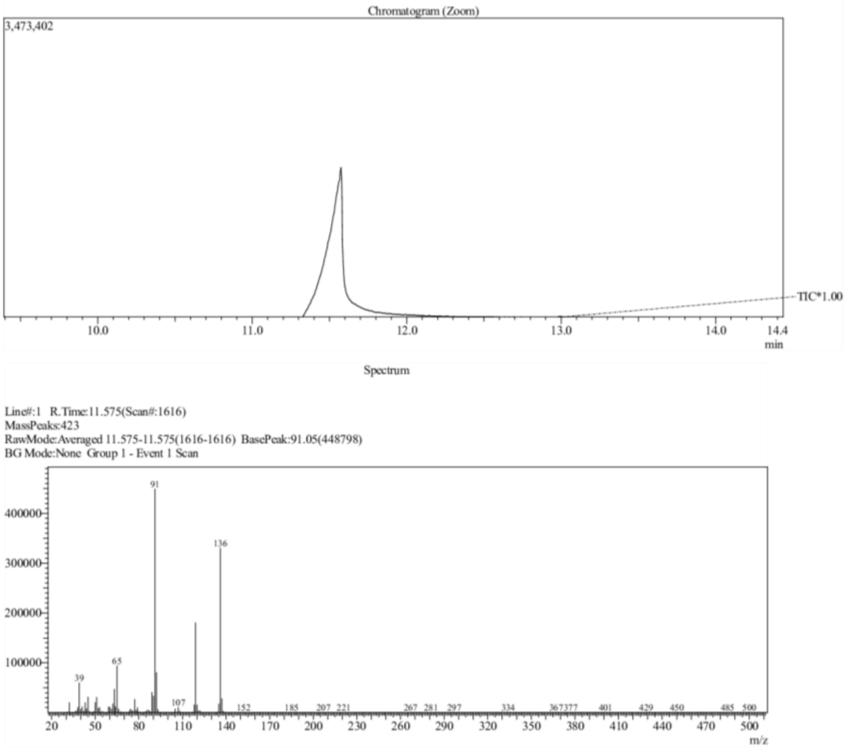

Figure S36. GCMS data in presence of 1-ethynyl-3-methylbenzene (**R**$_3$) to 3-methylbenzoic acid (**P**$_3$).

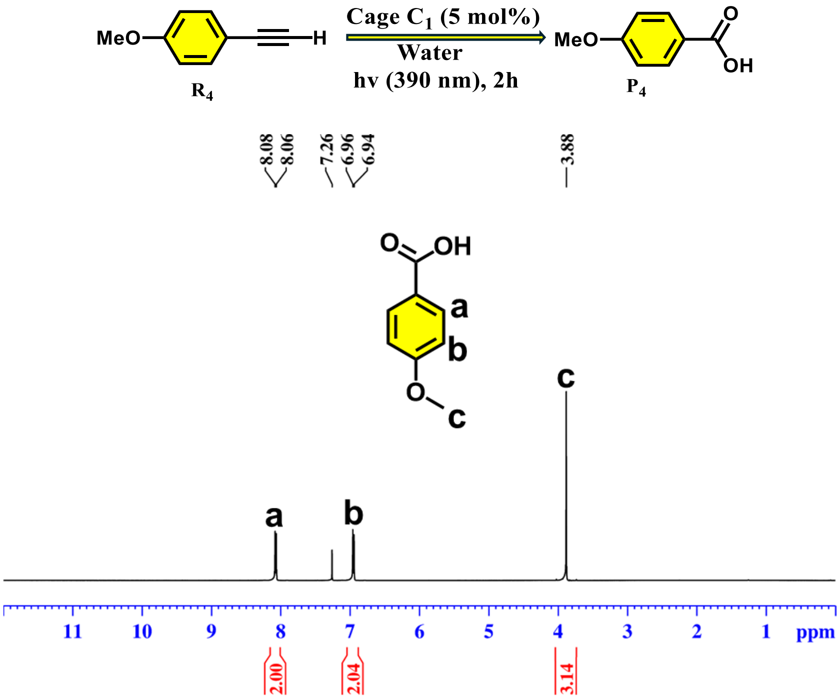

Figure S37. The ^{1}H NMR spectrum of the irradiation of **R**$_4$ ($CDCl_3$, 298 K).

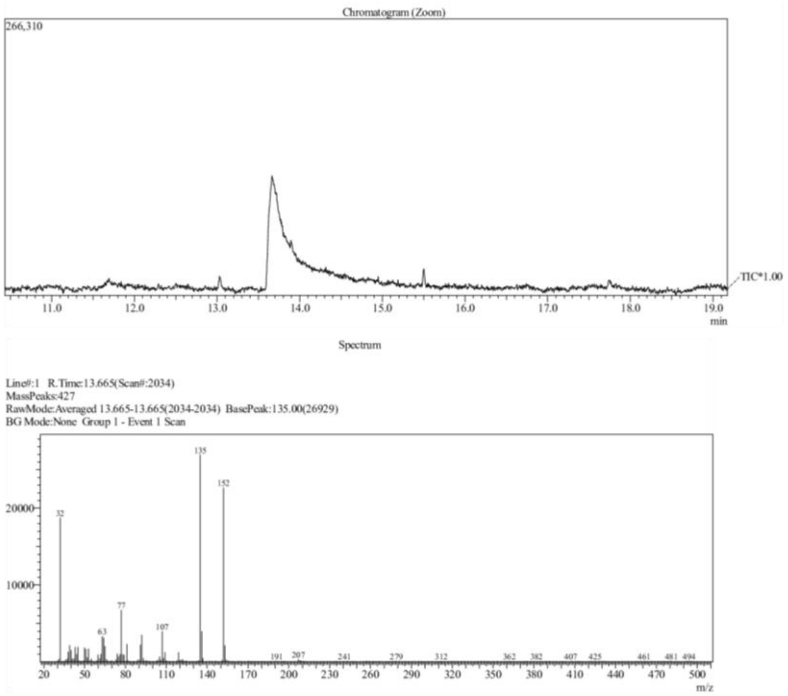

Figure S38. GCMS data in presence of 1-ethynyl-4-methoxybenzene (**R4**) to 4-methoxybenzoic acid (**P4**).

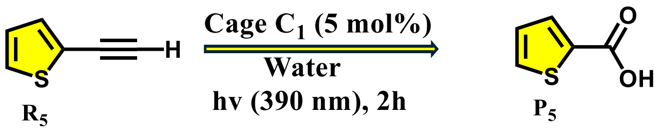

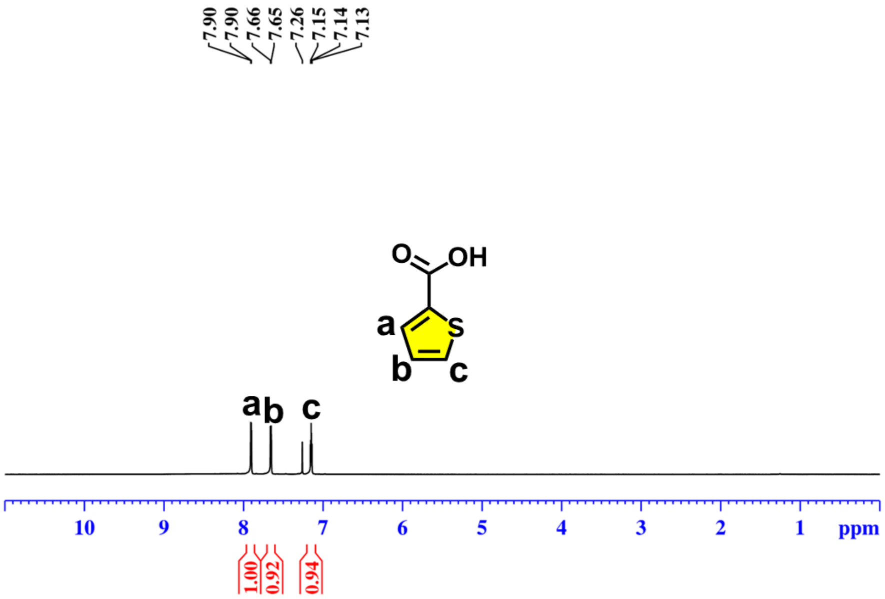

Figure S39. The ^{1}H NMR spectrum of the irradiation of **R5** ($CDCl_3$, 298 K).

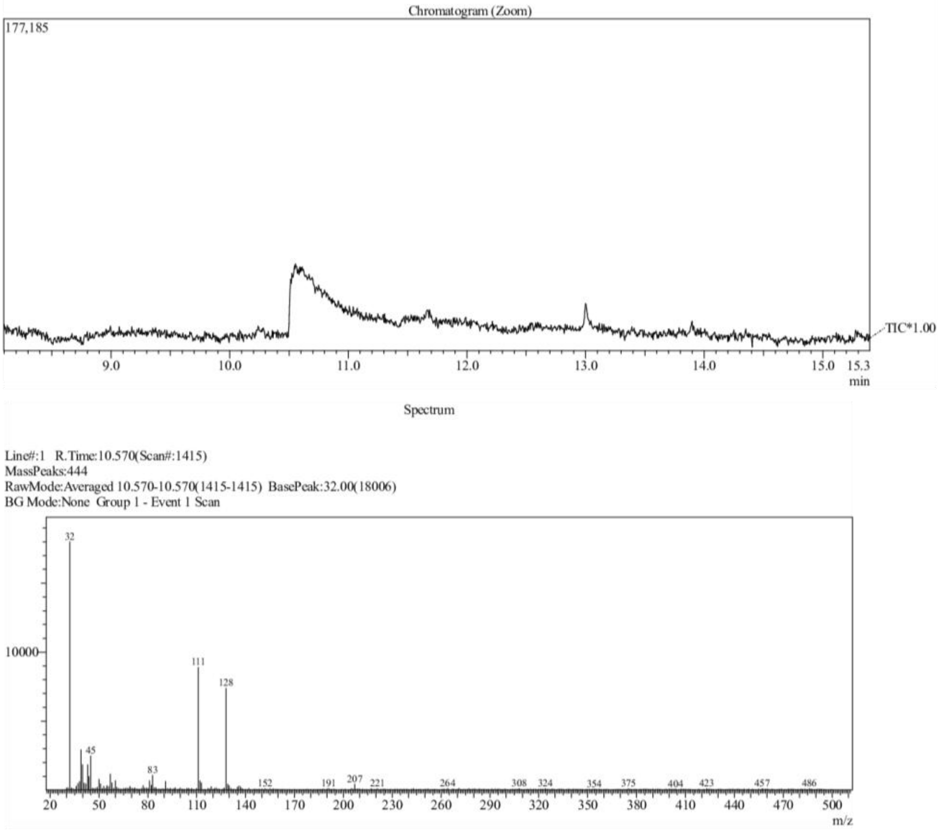

Figure S40. GCMS data in presence of 2-ethynylthiophene (**R_5**) to thiophene-2-carboxylic acid (**P_5**).

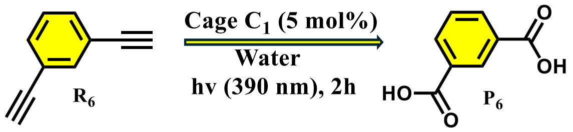

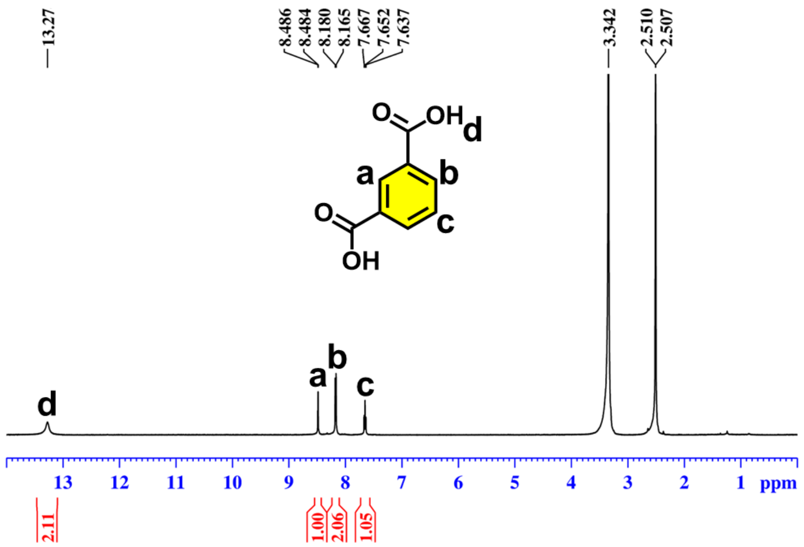

Figure S41a. The ^{1}H NMR spectrum of the irradiation of **R_6** ($CDCl_3$, 298 K).

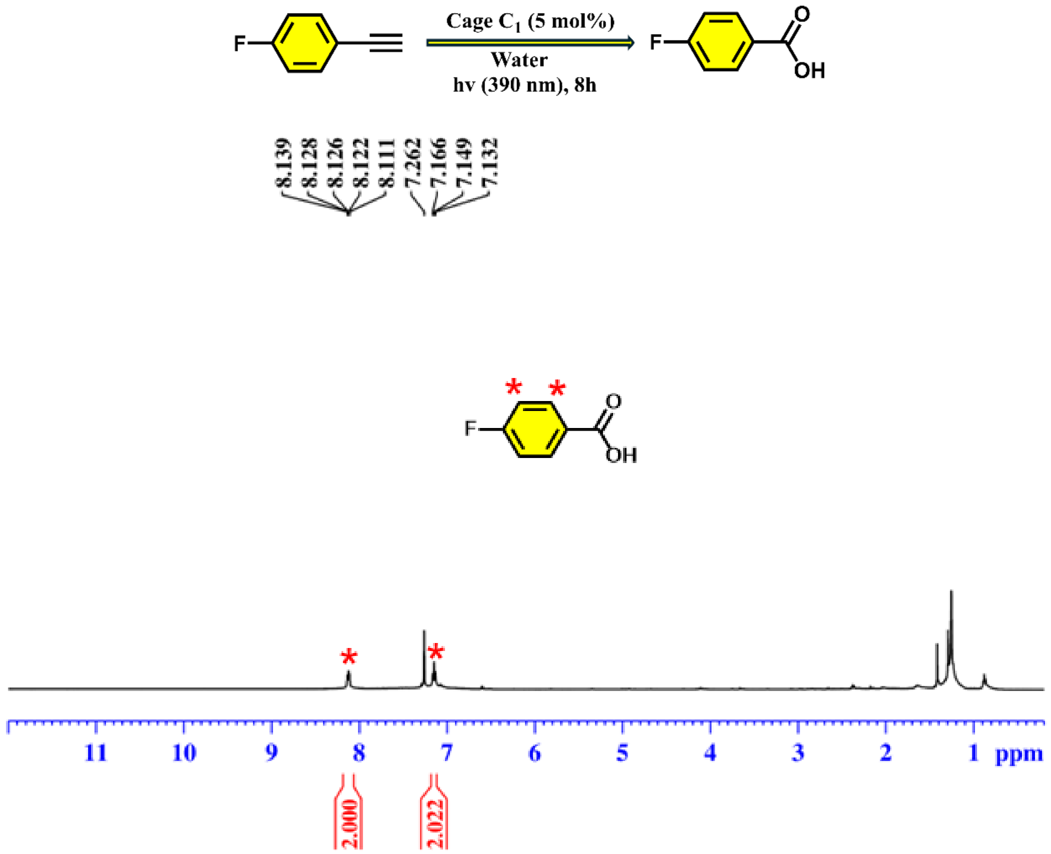

Figure S41b. The ^{1}H NMR spectrum of the irradiation of 1-ethynyl-4-fluorobenzene ($CDCl_3$, 298 K).

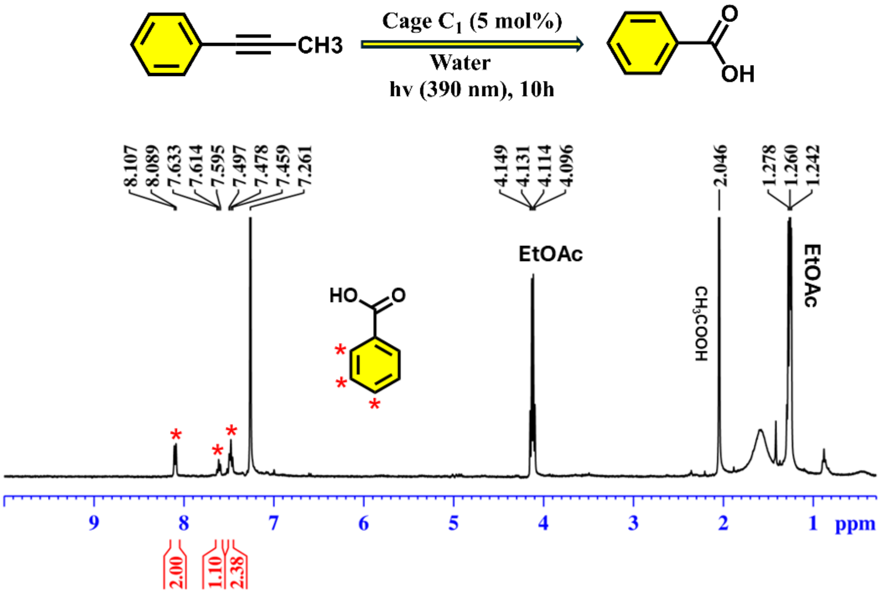

Figure S41c. The ^{1}H NMR spectrum of the irradiation of prop-1-yn-1-ylbenzene ($CDCl_3$, 298 K).

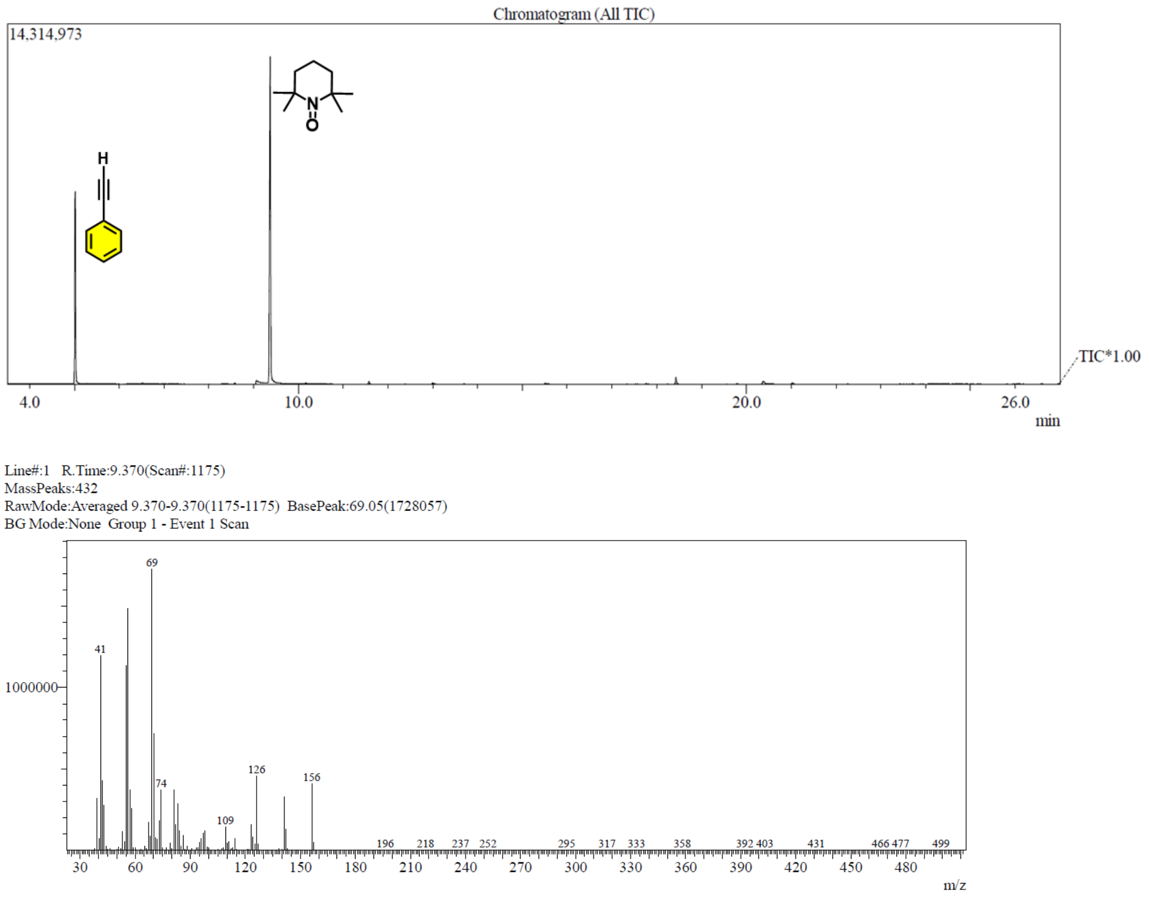

Figure S42. GCMS data of the irradiation of ethynylbenzene in presence of TEMPO.

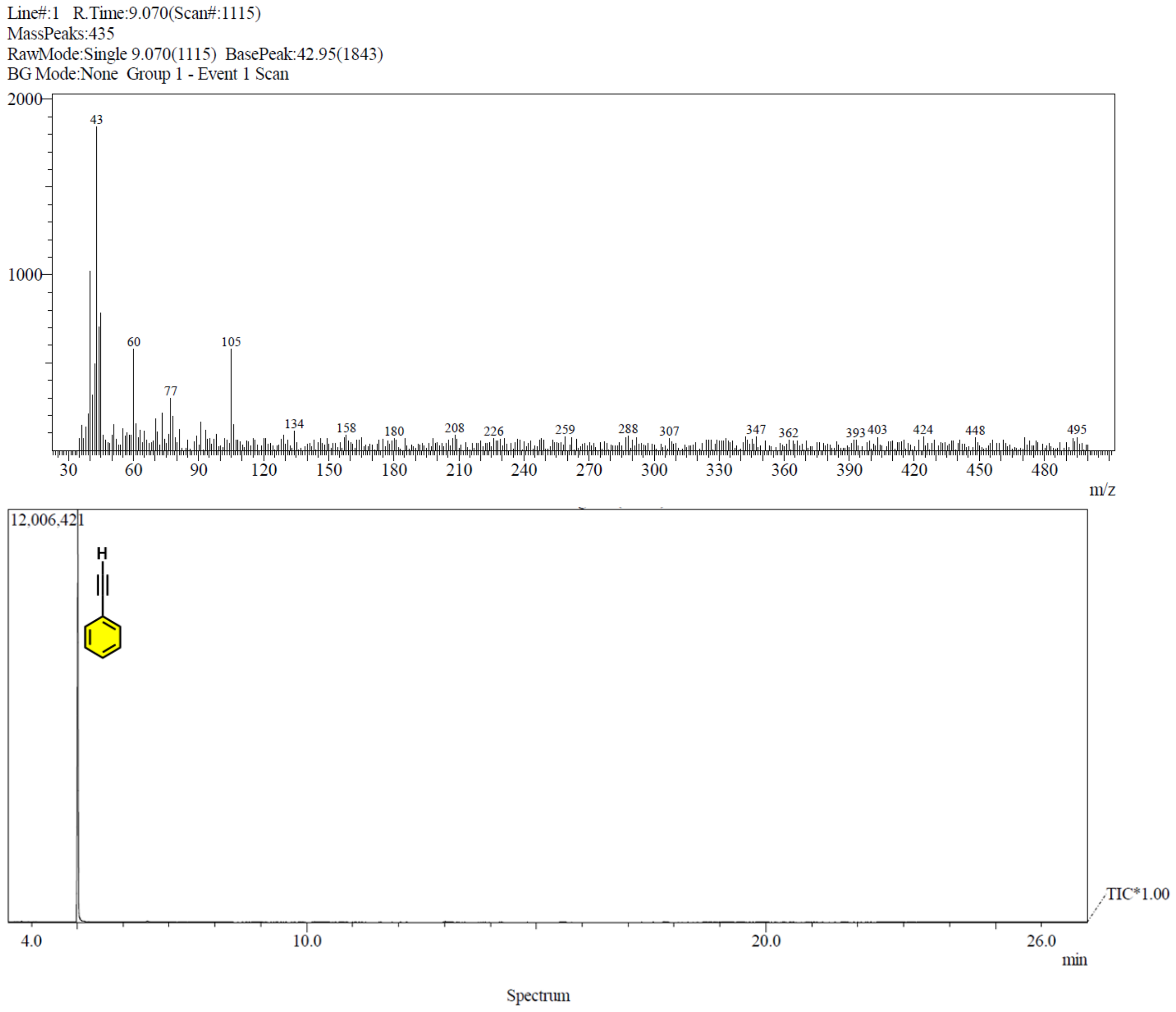

Figure S43. GCMS data of the irradiation of ethynylbenzene in presence of **C_3** cage.

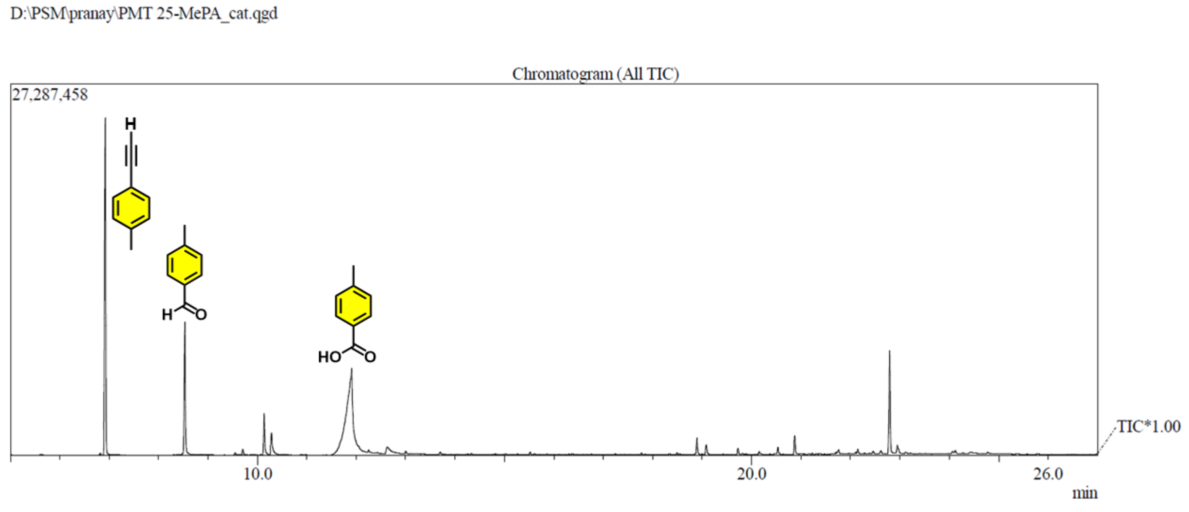

Figure S44. GCMS data of the irradiation of 1-ethynyl-4-methylbenzene to 4-methylbenzoic acid [1h uv light (390 nm) irradiation].

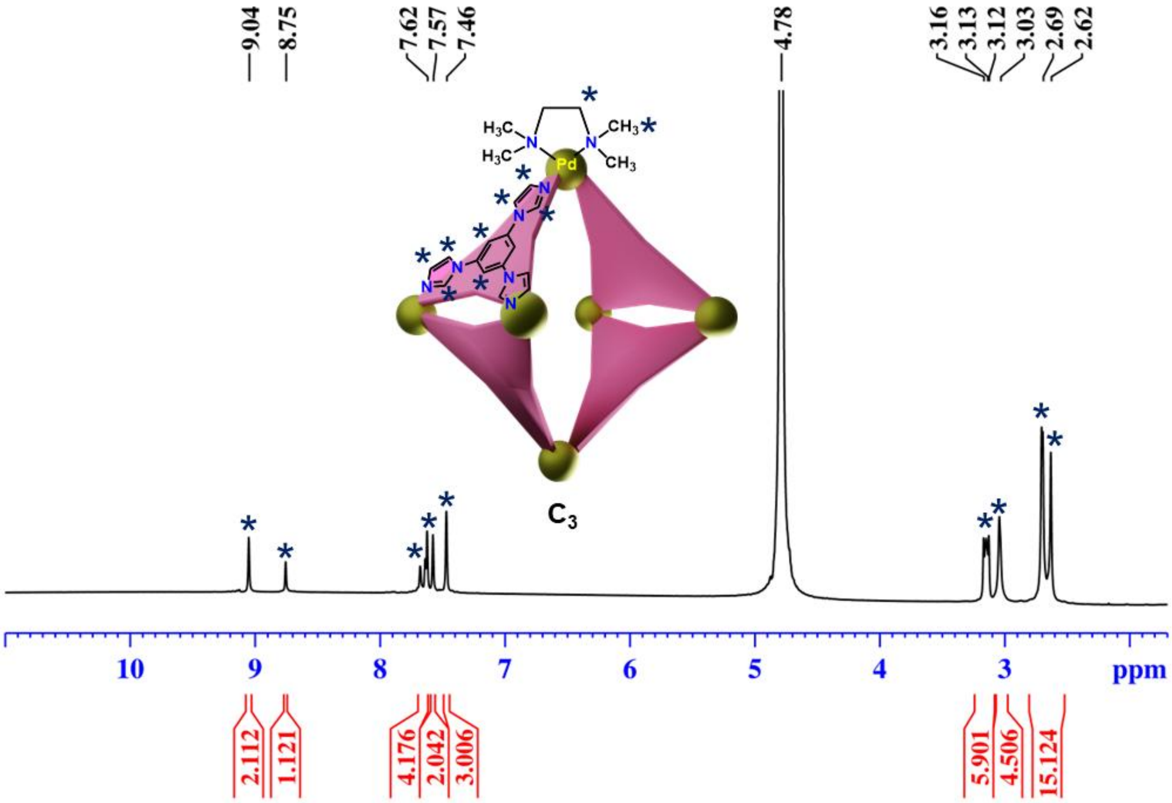

Figure S45. ^{1}H NMR spectra of cages **C3** (D_2O, 298 K).

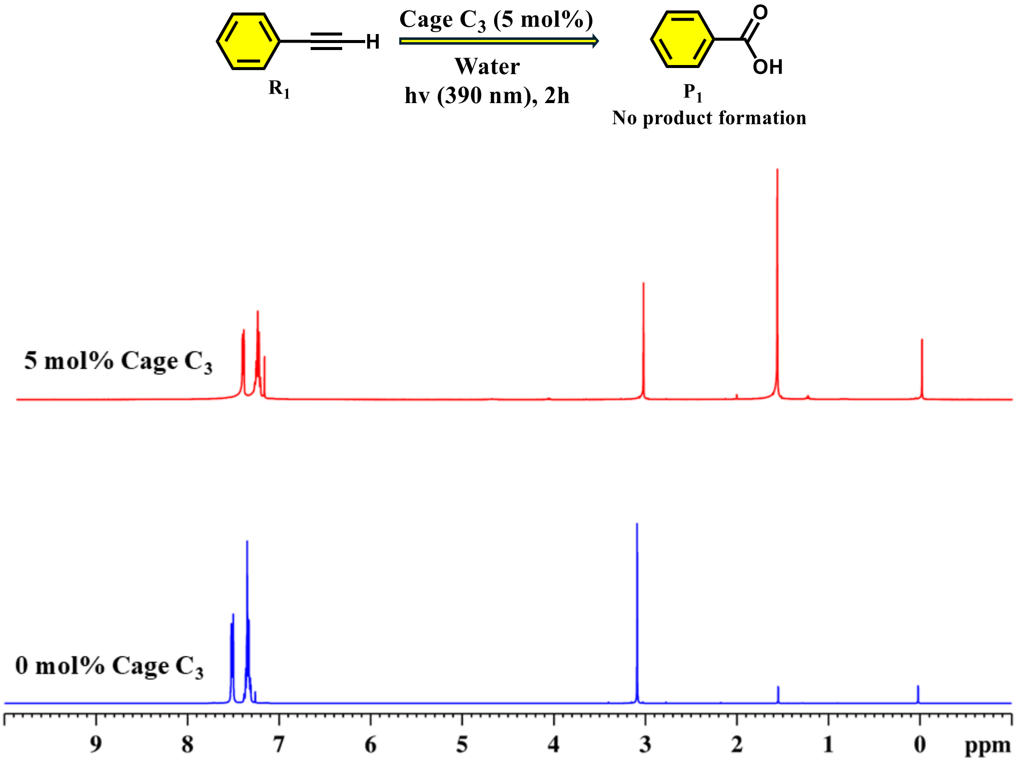

Figure S46. The ^{1}H NMR spectra of the irradiation of **R1** in presence of **C3** cage ($CDCl_3$, 298 K).

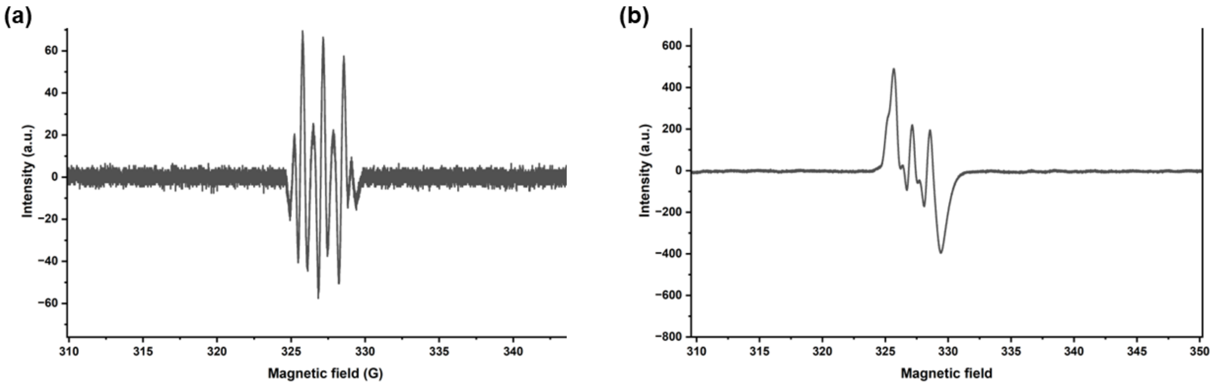

Figure S47. EPR spectra of $\mathbf{C_1}$ with light irradiation for the detection of (a) $O_2^{\bullet-}$ and (b) $^{\bullet}OH$.

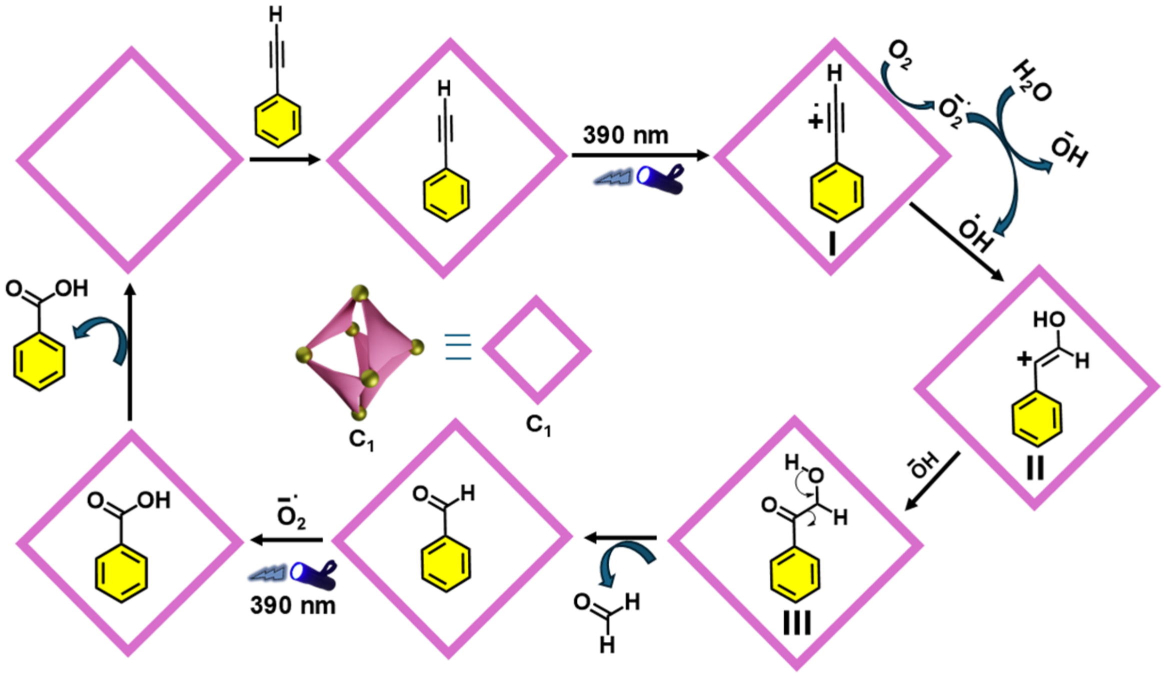

Figure S48. Plausible pathway for the oxidation of ethynylbenzene to benzoic acid catalyzed by cage $\mathbf{C_1}$ in aqueous medium.

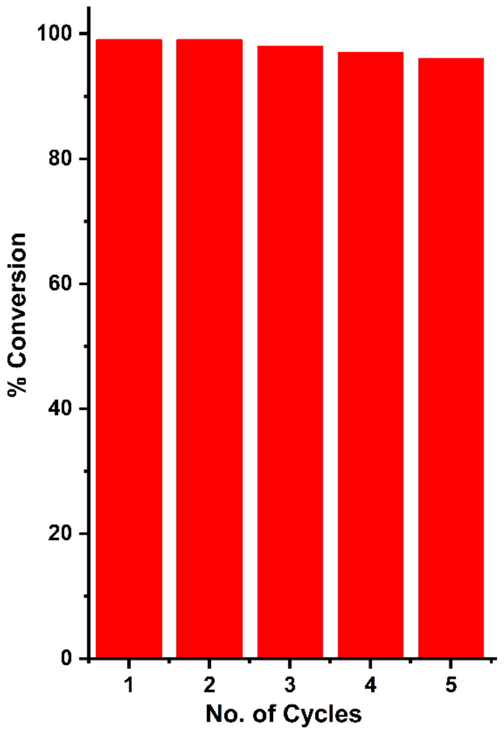

Figure S49. Recyclability of cage **C_1** as photocatalyst for the reaction of ethynylbenzene **R_1**.

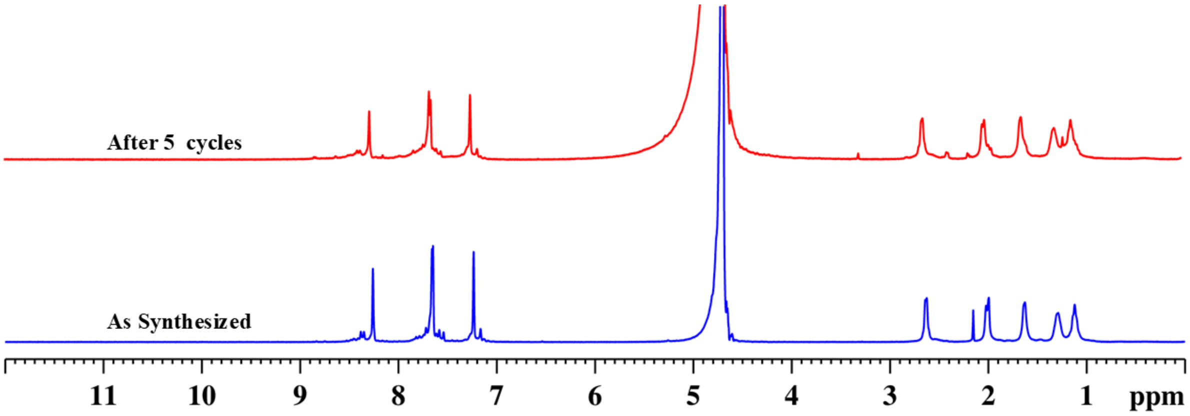

Figure S50. ^{1}H NMR spectra of **C_1** as synthesized (below) and after 5 cycles (above) of use for the reaction of ethynylbenzene **R_1**.

7. Relative Stabilities of the Octahedral (C_1) and Double-Square (C_2) Architectures

All theoretical calculations were performed using Gaussian 09 package. Geometry optimizations and single-point energy evaluations (B3LYP/def2-SVP, PCM) show that the octahedral cage **C_1** is **33.41 kcal·mol^{-1}** more stable than the double-square analogue **C_2** when built using the **1R,2R-dch** acceptor. This large energy difference explains why the octahedral topology forms with the rigid 1R,2R-dch–Pd(II) corners, whereas the flexible TMEDA–Pd(II) corner yields the double-square structure.

Species	Method	Total energy (a.u.)	Total energy (kcal/mol)
Octahedral (**C_1**)	(B3LYP/def2-SVP, PCM)	-6471.014193	-4060622.881
Double-Square (**C_2**)	(B3LYP/def2-SVP, PCM)	-6470.960948	-4060589.469

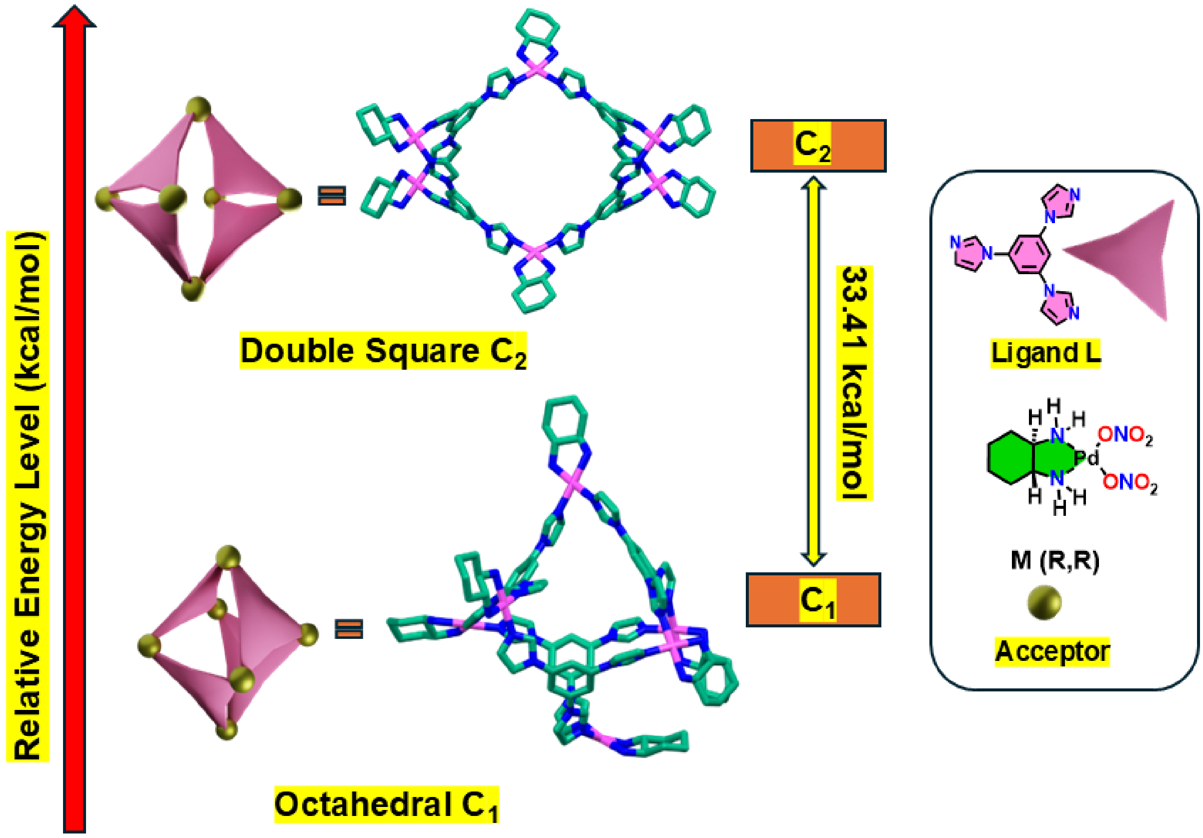

Figure S51a. DFT optimized structures and the relative stabilization energies of double-square (**C_2**), and octahedral (**C_1**) architectures. **C_1** is relatively more stable compared to **C_2**.

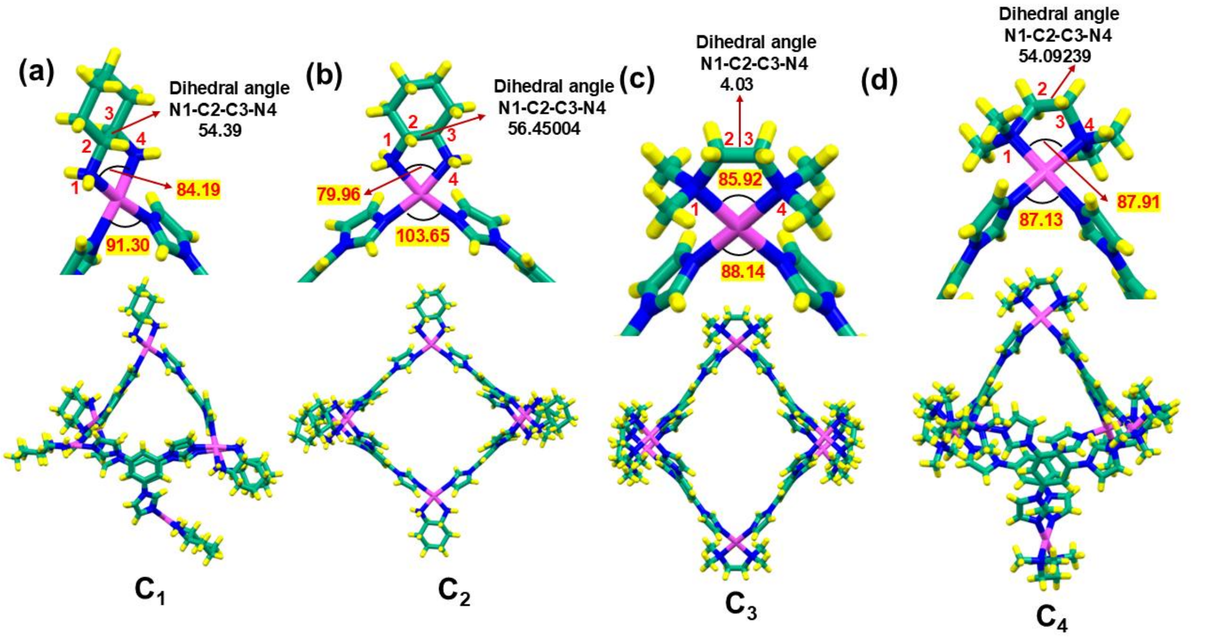

Figure S51b. (a) Crystal structure of Octahedral (**C_1**) (b) DFT optimized structure Double Square (**C_2**), (c) Crystal structure of Double Square (**C_3**) and (d) Crystal structure of Octahedral (**C_4**)[14].

Cage	Chelating ligand	Observed N–Pd–N bite angles	Deviation from ideal 90°
Octahedral (**C_1**)	1R,2R-dch<br>Imidazole ligand	84.19<br>91.30	5.81<br>1.3
Double Square (**C_2**)	1R,2R-dch<br>Imidazole ligand	79.96<br>103.65	10.04<br>13.65
Double Square (**C_3**)	TMEDA<br>Imidazole ligand	85.92<br>88.14	4.08<br>1.86
Octahedral (**C_4**)	TMEDA<br>Imidazole ligand	87.91<br>87.13	-2.09<br>-2.87

Square-planar Pd(II) coordination ideally requires cis N–Pd–N angles of 90°. In the octahedral cage **C_1**, constructed from the rigid 1R,2R-dch–Pd(II) acceptor, the observed N–Pd–N angles are **84.19° and 91.30°**, representing only modest deviations (−5.81° and +1.30°) from the ideal value. These small distortions are readily accommodated in the octahedral M_6L_4 topology.

In contrast, the DFT-optimized double-square cage **C_2** built from the same rigid 1R,2R-dch-–Pd(II) acceptor requires a much wider angular range (**79.96° and 103.65°**, corresponding to deviations of −10.04° and +13.65°). Such large distortions from the ideal square-planar geometry are not compatible with the geometrically locked bite angle imposed by the cyclohexanediamine chelate, rationalizing why this topology is not experimentally observed.

By comparison, in the experimentally observed double-square cage **C_3** (Previously reported), assembled using the flexible TMEDA–Pd(II) acceptor, the N–Pd–N angles (**85.92° and 88.14°**) remain close to 90° (deviations of −4.08° and −1.86°), demonstrating that TMEDA can

accommodate the angular distortions required for double-square-based architectures without incurring significant strain.

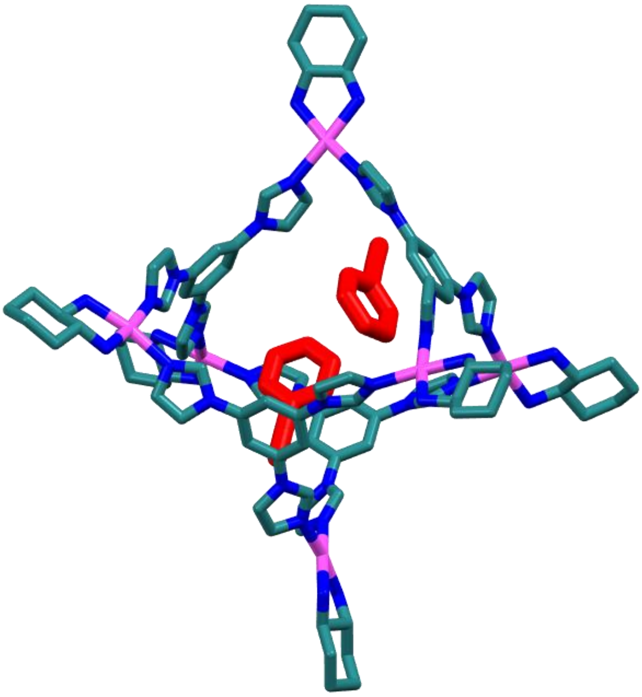

Figure S52. Geometry optimized structure of **$R_1 \subset C_1$** using the semiempirical method (xTB). Hydrogen atoms are not shown for clarity.

Calculation of Binding Energy of R_1 within the cavity of C_1

The single point energy of the cage, guest, and host-guest complexes were calculated using semi-empirical xTB method at gas phase.

Energy of cage = −398.643743724214 Eh

Energy of guest molecule = −40.199195458630 Eh

Energy of complex (cage + guest molecule) = −438.894000350689 Eh

Now binding energy would be :

Energy of complex (cage + guest molecule) – (Energy of cage) – (Energy of guest molecule)

= −438.894000350689 Eh −(−398.643743724214 Eh) −(−40.199195458630 Eh)

= −0.051061167845 Eh

1 Hartree = 27.2114 eV

so,

−0.051061167845×27.211386 ~= -1.38944504425222437 eV

1 Hartree = 627.5095 kcal/mol

so,

−0.051061167845×627.5095 ~= **−32.0 kcal/mol**

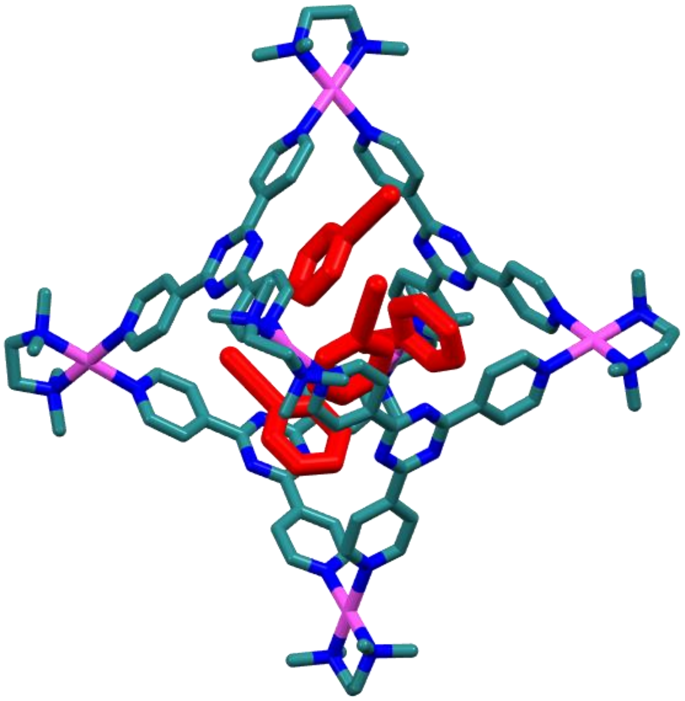

Figure 53. Geometry optimized structures of **TPT cage** + $\mathbf{R_1}$ using the semiempirical method (GFN2-xTB). Hydrogen atoms are not shown for clarity.

Coordinates of $\mathbf{R_1 \subset C_1}$

Pd	5.20985400261479	-5.96921160306843	-2.93437356724170
Pd	5.04972413616517	6.25328927514763	-3.78229858638516
Pd	4.81990143790214	0.75700128447176	7.32222874612987
Pd	-5.28896980947679	-1.15116589609811	-6.56872876948032
Pd	-5.64024465470786	-4.96690865624204	4.00830251554490
Pd	-5.66885993571747	6.08975193429146	1.96564407980378
C	6.07481565594354	-0.76152854084318	5.03900758384866
H	6.39867419042179	-1.38781632699793	5.85351669879181
C	6.28840329274792	-0.94305507036473	3.70692272757538
H	6.86287820685377	-1.69110418557516	3.20199956387274

N	5.67568508924932	0.10086429278771	3.05893857107024
C	5.59973553171035	0.26671244529983	1.65746787126588
C	5.60074246007240	1.54053409481902	1.09380849432037
H	5.74382545705981	2.39683649547293	1.73196831246269
C	5.47486987917161	1.69827313473690	-0.28829786352799
N	5.37971701297409	2.99349767661738	-0.84695667450740
C	5.55581722719738	3.38158994484283	-2.14180516182666
H	5.91168690839988	2.73841090680932	-2.92378313742258
C	4.93223200972726	5.13759941014397	-1.06678602449941
H	4.67674771863935	6.17513634808496	-0.90803290387049
N	5.28882106696500	4.65885237046765	-2.30050376122675
C	4.98681169284109	4.12108495113212	-0.16446983681013
H	4.76652913229032	4.13203242331185	0.88106484445730
C	5.38533966265303	0.56848411796248	-1.10063534157142
H	5.32654173140558	0.68354175909044	-2.16968171252864
C	5.39246304337003	-0.71175020725809	-0.54307105433460
N	5.23209835692403	-1.83820869735877	-1.38550119265537
C	5.62646641547316	-3.12597838631395	-1.18390930343186
H	6.18749275850625	-3.45597189242178	-0.33041635763819
C	4.59652404015285	-1.82145014681880	-2.60389744575124
H	4.15065134283869	-0.94500578370843	-3.02287322325360
C	5.48726751331660	-0.85966604107837	0.83931790626423
H	5.45966315791500	-1.84177848636672	1.28332048311221
C	5.11584332909463	0.87497174159525	4.02839071208436
H	4.55431495450475	1.76761786808239	3.82815693068201
N	5.33130052976266	0.37562337356432	5.22629564660294
C	2.78387035712399	-7.09488213875538	-1.59907046785209
H	3.53076140041180	-7.75541585840800	-1.18986299888398
C	1.45161104976336	-7.05989679452373	-1.32505396631359

H	0.87049036259770	-7.70822126475550	-0.70386568196262
N	0.91425267355533	-6.04639643732841	-2.08133526593019
C	-0.43227582332992	-5.62447065969076	-2.03864014109932
C	-1.04362262892756	-5.02834907382571	-3.13809210095845
H	-0.49266422402534	-4.90453956754411	-4.05619357889646
C	-2.37061768084388	-4.60115428769896	-3.06090392418739
N	-2.94737795331542	-3.98517689115157	-4.19609259740720
C	-2.60279752502416	-4.24652028662496	-5.49904047079215
H	-1.94609169253728	-5.03990626400564	-5.78803491784496
C	-3.30737982422051	-3.37966456169638	-6.27708146165381
H	-3.29083683828953	-3.28296320935531	-7.35025671789848
N	-4.07937691493303	-2.58053242391656	-5.47450625750530
C	-3.85305415316792	-2.97326212112511	-4.23791988213899
H	-4.31403041052920	-2.54778053058282	-3.36754687875389
C	-3.09731974270795	-4.77572861324766	-1.88237684623026
H	-4.13814322702614	-4.49682224934696	-1.83820976188305
C	-2.48791903325701	-5.37692513157680	-0.78198347500403
N	-3.17791910763938	-5.59862177863547	0.43475126207008
C	-3.00702846998916	-6.70481217911960	1.22979506176704
H	-2.41673018096542	-7.54957150057133	0.94172843254829
C	-3.77121423520689	-6.51549033683340	2.34031198056088
H	-3.88478511235929	-7.16792167557408	3.19224957402755
N	-4.40763928513661	-5.30456691245026	2.24707970653779
C	-4.04182171320942	-4.78393102396187	1.09358455023505
H	-4.36620196462701	-3.83344862886083	0.71511233252570
C	-1.15763909188109	-5.78482836229322	-0.85933563899814
H	-0.68683114120441	-6.21945363754098	0.00774495361050
C	1.94444529387463	-5.51267681194839	-2.79126522767730
H	1.83176186369766	-4.70091406243766	-3.48551269968497

N	3.08149216356677	-6.11379509124049	-2.50973162501872
C	2.26986152275602	2.27563870399736	7.39510276805949
H	2.93909572302438	2.96284629605143	7.88847558369981
C	0.95179869796779	2.45051604463176	7.10711610383182
H	0.29972962303598	3.25823183389286	7.36549874106837
N	0.53041565688457	1.31479722819998	6.45830855937594
C	-0.76864933446787	1.12994167431447	5.93209580964588
C	-1.37977699490720	-0.12143703866340	5.89609398068331
H	-0.86366458633401	-0.98221090915140	6.29075451070401
C	-2.67066481118214	-0.25930153990028	5.37814068179297
N	-3.27190308343172	-1.54062929411345	5.37818608608804
C	-3.06567985056698	-2.50994097044782	6.32895781419752
H	-2.48952604739598	-2.35218761957913	7.21667065749389
C	-3.77985177365352	-3.60227119947778	5.94153581551045
H	-3.85015259568897	-4.55763619503631	6.43428758179766
N	-4.42130426865429	-3.32721273891043	4.76196487395386
C	-4.10756696685454	-2.08570207738969	4.45441240269358
H	-4.44906578750367	-1.56557184281814	3.57972831013412
C	-3.35368777883418	0.85571452836929	4.88787139356233
H	-4.36964019948263	0.75865659557295	4.53873783789881
C	-2.74216578424352	2.10850865591315	4.92105392414624
N	-3.39398137899876	3.27914188165520	4.46158677580144
C	-4.17337581381402	3.45593776559217	3.36340317004932
H	-4.44176421192905	2.66377672334496	2.69121110309815
C	-3.26400881919313	4.51448202195453	5.04705399371277
H	-2.74428255913461	4.67739967921156	5.96804609007364
C	-1.45164971308894	2.23808427833435	5.43159564434492
H	-0.97472482153683	3.20531416454448	5.43221541369402
C	1.61404840505786	0.49435073246368	6.38472926681817

H	1.59701197645120	-0.47481938053848	5.92384552392271
N	2.67483652324622	1.04998238334402	6.92979169955074
C	2.72423095911631	5.37037790166665	-5.43711916993120
H	3.48988907107721	5.39331646988601	-6.19580820988870
C	1.42467898468977	4.98873218419971	-5.56359345739669
H	0.88886249684105	4.69657165738076	-6.44171042083071
N	0.84324076561017	5.12594567008556	-4.32561795473533
C	-0.48328889316066	4.76360301859110	-4.00564074965126
C	-1.11401651721113	3.76555018167283	-4.74820812039578
H	-0.58372346927953	3.27297441617368	-5.54690849744935
C	-2.42345817119331	3.38366284276815	-4.46205065867987
N	-3.02041261139058	2.38654749653633	-5.27249912644908
C	-2.80528269732083	2.25202829549487	-6.62178782601820
H	-2.23988901799060	2.95315169712484	-7.19938605810803
C	-3.49538146537639	1.14982557195448	-7.02482821113244
H	-3.55674512291397	0.72505226601155	-8.01478005725378
N	-4.13072885246788	0.59560351451610	-5.94326725353067
C	-3.83564519312875	1.36142877113010	-4.91237754091963
H	-4.17721021515773	1.20005240010211	-3.90784634939188
C	-3.10771798869440	3.98347159209382	-3.40578052537365
H	-4.13574073003997	3.72331361810671	-3.20870653550810
C	-2.47691148043644	4.97257795612748	-2.65013538307495
N	-3.14063645441396	5.60404886335534	-1.57338073644363
C	-2.93762525485719	6.90044288643978	-1.17235833283928
H	-2.33160042149422	7.59759200982052	-1.71200334212883
C	-3.70078944970169	7.09535533887197	-0.06247603794785
H	-3.79005118339452	7.98844966260573	0.53370568245597
N	-4.36842305930883	5.93544815602338	0.23410052722469
C	-4.02111270373495	5.06500641262352	-0.69126454225833

H	-4.36857124610968	4.05101044571640	-0.73782932152903
C	-1.16982258681711	5.36078186684122	-2.95140453225610
H	-0.69788848500385	6.13663026190104	-2.37118225619664
C	1.81787580466398	5.59553961780349	-3.50017971178986
H	1.66613486823936	5.81232352653384	-2.46070653911517
N	2.96024191226174	5.73820138911693	-4.13712287944056
C	7.66533655800273	-7.51351938121937	-3.57333678364571
H	7.64920419505546	-8.04290371676295	-2.61043916964092
C	9.06086551335382	-7.64616798679594	-4.20048192095174
H	9.10219048412601	-7.08082394997054	-5.13385947770626
H	9.82469845694804	-7.25958503122018	-3.52575422773776
C	9.32929225541717	-9.12710986907063	-4.48730799034923
H	9.36987923409054	-9.69108605862960	-3.55485423564483
H	10.31088860787607	-9.20866388861670	-4.95922782015606
C	8.27210919423702	-9.70143235629705	-5.41086222759937
H	8.47471922235029	-10.76063676028694	-5.58529091113480
H	8.29826122331965	-9.20278338981937	-6.38036074358129
C	6.87753534577753	-9.58240886011331	-4.78874467306611
H	6.13396690746510	-9.98844526388218	-5.47451935908950
H	6.84349020358875	-10.15492371414260	-3.85948697127564
C	6.59528819477071	-8.10058156129442	-4.49756627092469
H	6.57983174334832	-7.54200365658221	-5.44304080740048
C	7.39029905078074	7.79175208751699	-4.78434059338773
H	7.46203889669040	7.20894163625873	-5.71296524053646
C	8.70843091845697	8.54709491088919	-4.55811378281349
H	8.66557073442977	9.09029532976429	-3.61172493634967
H	9.54853053910907	7.85345458357291	-4.52319824084333
C	8.90671785599408	9.53882781157686	-5.70876708159633
H	9.83056896589352	10.09151858722523	-5.52394023925929

H	9.03186856972189	9.00440855367107	-6.65106154677759
C	7.74353847469361	10.50814454879329	-5.79666293726375
H	7.89920931000886	11.19173087774569	-6.63438008126280
H	7.67997334343329	11.11352126937826	-4.89182301520230
C	6.42569907532348	9.76338224202450	-6.03158438316780
H	5.60391958322514	10.47791800327030	-6.07816995808082
H	6.47507584809834	9.22709133953566	-6.98155093405252
C	6.21411036451262	8.76689690563614	-4.88181825969707
H	6.11465159906964	9.31881376043466	-3.93736691420351
C	5.82961345880850	0.55790373686408	10.13116863206102
H	5.94933031620873	-0.53312180396844	10.08976653772291
C	5.83277385758018	1.01342393734150	11.59811462132052
H	5.04786889906062	0.50729947158534	12.16011431914991
H	5.66035696619837	2.09051670226611	11.65115786540259
C	7.19839239960323	0.68489535219899	12.20943330292452
H	7.20009711782239	1.03929418429255	13.24281546938947
H	7.35104950390566	-0.39452331566358	12.23679199201963
C	8.31447743652751	1.36203373748150	11.43781029730673
H	9.27777956562667	1.10062383220976	11.88195839176191
H	8.21577850395309	2.44664613680397	11.49326840927417
C	8.32705392469323	0.90312921164324	9.97629600938866
H	9.12819680828744	1.41095475145765	9.43934653664224
H	8.50698739809817	-0.17319201990116	9.93464035805109
C	6.96265232621960	1.22706149529778	9.34893971093663
H	6.81482611150002	2.31550768901723	9.34499776116034
C	-7.61251153858960	-2.13273075366882	-8.18754483581571
H	-8.38681796281880	-1.90802353685517	-7.44214881966777
C	-8.18157087458357	-3.10316139253059	-9.23332056313490
H	-8.53850754140312	-4.01605757946667	-8.75649714519924

H	-7.40458434188318	-3.36781088774953	-9.95359446339905
C	-9.33922347686805	-2.41517548827850	-9.96285948007078
H	-9.71642751370418	-3.10153235578550	-10.72395036483705
H	-10.15817003066138	-2.21720240104438	-9.27043467888126
C	-8.87755550372410	-1.12946731615333	-10.62298998317492
H	-9.72303942839517	-0.64864229915915	-11.11913231335271
H	-8.12966052263579	-1.34013619269599	-11.38804649200485
C	-8.31413646965799	-0.15228078023962	-9.58614499971765
H	-7.97543571305182	0.75541767286608	-10.08576156776141
H	-9.09724731628877	0.11454398697767	-8.87336371240052
C	-7.15096659007176	-0.83230177630685	-8.84968242080285
H	-6.34690509769535	-1.04887941197450	-9.56721250827382
C	-7.57865356716722	-7.07801461783958	4.75226903430051
H	-6.81308403774570	-7.60774284302283	5.33677135643614
C	-8.74158807498778	-8.03689787506361	4.45880001563217
H	-9.48391202112856	-7.53697204794607	3.83308942596001
H	-8.38591118348146	-8.92384408178882	3.93434519650202
C	-9.38546472189723	-8.44861051665717	5.78651030127529
H	-10.22965413322598	-9.10500402071399	5.56590486199395
H	-8.67806772856114	-9.02107374578038	6.38726258341346
C	-9.87264796727412	-7.23338643358022	6.55334985539075
H	-10.30876926490258	-7.55126520097108	7.50262330076652
H	-10.65356696619198	-6.71643686535503	5.99456635023058
C	-8.71556221254868	-6.27681272905420	6.85718684059945
H	-9.09046594821087	-5.40674428119735	7.39623299561064
H	-7.98042180991199	-6.78482949874348	7.48490792695945
C	-8.06451464866138	-5.85397782116013	5.53212043007952
H	-8.79684990079572	-5.30283175145701	4.92747965107730
C	-8.08953025611460	7.85393632238910	1.81583419834116

H	-8.80781794241635	7.05332419585482	1.59474054425743
C	-8.72157084484273	9.21151604718012	1.47402788127803
H	-7.99732247530691	10.01043407452274	1.64666925823123
H	-9.02770015209620	9.24045976820665	0.42825835523309
C	-9.94111644854386	9.42665738785411	2.37525293354136
H	-10.70867371149413	8.68338001943603	2.15675466194948
H	-10.36382574902881	10.40707509598675	2.14554293765262
C	-9.54996585845655	9.37332741477035	3.84017795583593
H	-10.43746389864285	9.51019052500575	4.46138365967348
H	-8.85831220493031	10.18135626834498	4.08057673075691
C	-8.92582363732889	8.01907192609507	4.19192427624737
H	-8.63983515201953	8.01073597578048	5.24378890722847
H	-9.65726718482325	7.22577682752525	4.02394831978406
C	-7.70006670731003	7.79447702182315	3.29474155712223
H	-6.94931720050687	8.56791357166838	3.50991397056859
N	5.27291856225116	-3.89838171335173	-2.18805752718716
N	-4.52300496157944	4.71838627081111	3.22129684123255
C	4.62766400444105	-3.09250647756438	-3.08788004316196
H	4.24822308606783	-3.47295167353781	-4.02366668565116
C	-3.96589911131898	5.39208374584811	4.27865429047771
H	-4.08589663629537	6.45530524024727	4.41862357881520
N	7.29395857725618	-6.09738104043066	-3.32081821845482
H	7.92184228125377	-5.71225189527777	-2.60795142679609
H	7.47418298638655	-5.56679740421129	-4.18442794316665
N	5.28741033196911	-7.90399475622421	-3.82471668907348
H	4.53318061472106	-8.14481757114994	-4.47609705464432
H	5.23470312126822	-8.58584771968903	-3.05520416312991
N	-6.93843540695779	-6.57391432643509	3.51112112243796
H	-6.54470014381279	-7.36853378793426	2.99681967802666

H	-7.68043781428341	-6.18455406351347	2.91305331985039
N	-6.88636632464438	-4.97369647780182	5.74204837921021
H	-7.20543515339407	-4.06664394247870	6.09667904635865
H	-6.32450623668514	-5.39652663489395	6.49396739511047
N	-6.44057015827552	-2.70145264763521	-7.47522437172216
H	-6.75464872892164	-3.46744958506144	-6.87119413924064
H	-5.82954121351239	-3.12635701936073	-8.18657204275571
N	-6.58643728296924	0.02463126319208	-7.77592704906692
H	-6.21378337212937	0.88029962567835	-8.19980933076890
H	-7.36578379802048	0.32058955574099	-7.17182043397714
N	4.98843257018249	7.95076983884486	-5.06882203357591
H	4.16606327881701	8.55877086825586	-4.99756965115300
H	5.00312143998948	7.59492261689962	-6.03474820111393
N	7.08545991943325	6.85061994041882	-3.67720763595113
H	7.78581744449939	6.10194158219766	-3.67444151289074
H	7.20243172487871	7.36140607444352	-2.79086936026625
N	4.56294847611272	0.90858825841058	9.44123440418696
H	3.79944017102836	0.35317777091570	9.84093945470069
H	4.35664573603989	1.89043154788960	9.66818186918970
N	6.86349267076538	0.74355774121422	7.94942073456691
H	7.51765556828768	1.27354569658115	7.36402525441414
H	7.19055836816571	-0.23173851657615	7.94206204886606
N	-7.06990796753196	6.46934552885092	3.52382501480354
H	-6.74489116691373	6.42356255717359	4.49483346583965
H	-7.80638574977420	5.75544161950340	3.43493361249166
N	-6.85740300213449	7.59664912350356	1.02838885590491
H	-7.11172697119638	7.44746515166762	0.04700013680918
H	-6.29697358741531	8.45951482750130	1.05364580655975
C	-0.97855400453545	-1.67983015014762	-2.99098879784020

C	0.34171262513371	-2.13195594696793	-2.98223719887957
C	0.93993977373962	-2.48138939237296	-1.77085576677732
C	0.21823656257575	-2.40412082090492	-0.59556057510085
C	-1.09615927199818	-1.96558658491329	-0.61058726911994
C	-1.68732901728100	-1.59576991189756	-1.80761137618284
H	-1.43694039836870	-1.40597623037653	-3.93119934660569
H	1.96654782821749	-2.81795088869274	-1.76109252009254
H	0.68162820212955	-2.68781569149859	0.33744811152119
H	-1.65549849645868	-1.90802116590756	0.31122114198024
H	-2.70643268951292	-1.23782383982415	-1.81640901545934
C	1.05026128849303	-2.25723305272463	-4.20335105075573
C	1.61906924940259	-2.39011109285732	-5.25369853589492
H	2.11410509829927	-2.49201597517129	-6.17628634932539
C	1.12053235463199	3.49513759649838	-0.93664407776305
C	-0.22296379746303	3.20650201805202	-0.69485963987096
C	-0.87409657303893	2.25827496282912	-1.48446624511229
C	-0.19633532060877	1.63019467070849	-2.51082136362007
C	1.13514998329985	1.92692624480124	-2.75526773476322
C	1.79086203814397	2.85628725213494	-1.96400158065867
H	1.62521382418693	4.22339121293812	-0.31907416468388
H	-1.91067997506615	2.02627728959590	-1.28913226926993
H	-0.70420407906358	0.90374649503102	-3.12543494016793
H	1.65470898006508	1.43730529604070	-3.56447167441551
H	2.82465826446944	3.09628068088810	-2.16266573466547
C	-0.92834858119017	3.90088043466966	0.32014796156019
C	-1.54177814266658	4.51345779332063	1.15169763795059
H	-2.05854342947051	5.07778552987602	1.87238103770570

Coordinates of **$R_1 \subset$TPT cage**

Pd	1.47228107974661	-1.75867757345079	9.13718183948600
N	3.06475630424969	-2.44185017460727	10.27720460365142
N	0.45653760731865	-1.66632096244745	10.94489436624275
C	3.13669654313472	-3.88919443673234	10.02432735582204
H	3.49194528360700	-4.07719127757058	9.00855565885004
H	3.83970705178868	-4.36921587775957	10.71307356483346
H	2.15813757793273	-4.36200048291430	10.16034417332599
C	4.35543554885540	-1.81734461270692	9.96897189853686
H	4.69716080089591	-2.09757665800691	8.97201996568928
H	4.27737595181183	-0.72882098683553	10.03926857431271
H	5.11832455528117	-2.14335335809698	10.68353857288420
C	2.74011415471320	-2.17229969386951	11.70043429410615
H	3.00429278641374	-1.13666042584274	11.93313048997891
H	3.33729448310757	-2.81372198266220	12.35917336376459
C	1.25991575830262	-2.41248066355536	11.94540083840164
H	0.99392608292508	-2.10015554183333	12.96210236003493
H	1.02540227545813	-3.47774909599860	11.86438335748680
C	0.39536092895514	-0.23692815413179	11.28678476619212
H	1.38579952240442	0.22663814204361	11.22481162151148
H	-0.29144584476040	0.28320103760458	10.61526613804057
H	0.02404756265692	-0.09823493633194	12.30753227002625
C	-0.89666742353310	-2.23087545794036	10.91987871686930
H	-0.87306095340626	-3.25979490326660	10.55034511744230
H	-1.32123414656720	-2.24950469059187	11.92905252550956
H	-1.55975275035831	-1.63128088389128	10.29560841063092
Pd	-4.68673634545251	7.98070875809584	2.22003096587005
N	-6.11568036397131	8.48544797925034	3.63280419397484
N	-4.86271630491542	9.95217137728352	1.60281658765464
C	-7.37747789806810	7.88245970398313	3.17828768501551

H	-7.33542340975531	6.79569813947143	3.27923072961627
H	-8.21747281721330	8.23659533915805	3.78485786438559
H	-7.58488165712110	8.14266827895580	2.13512530782802
C	-5.79902840836728	8.02265295094571	4.98797861567494
H	-4.80289693118536	8.36554705684354	5.28319692082149
H	-6.51770203002084	8.42669645214163	5.70855178147803
H	-5.84702942529341	6.93481168120057	5.05025582231270
C	-6.21835073876385	9.96660996441777	3.65020981364421
H	-7.17459921839578	10.28115705153262	4.08425290018322
H	-5.42647286699648	10.36912642299054	4.28848405889450
C	-6.08670345812210	10.50463477465463	2.23554296631232
H	-6.05006916283166	11.60016164538180	2.25277016989561
H	-6.95549316433010	10.22092926050526	1.63449259515956
C	-3.66409453889175	10.65077730836754	2.09032596766375
H	-2.78475481086531	10.33252274114410	1.52662012906626
H	-3.76445225287652	11.73278490525432	1.95555757455733
H	-3.50235475956436	10.45983205150083	3.15644601242789
C	-4.97595079862102	10.10737910689848	0.14887549709756
H	-4.04406111406738	9.83442040782607	-0.34761040049034
H	-5.79348490338157	9.49127041697857	-0.23709706105832
H	-5.19381005686068	11.14915516413735	-0.10862267235369
Pd	8.04181746275802	5.00000253404801	-0.30474101683423
N	8.72854716266360	6.81912862429614	-1.02402806893187
N	9.97972424576485	4.74971474887608	0.38744060621449
C	8.88113340838289	6.64967852010820	-2.47690084887772
H	9.46665043749162	5.75422465335430	-2.71045509186963
H	7.90136155006393	6.57695719318973	-2.95411783519344
H	9.39652350381646	7.51039535910749	-2.91565556024659
C	7.83880712588009	7.95067054090104	-0.74369033476524

H	7.62127623818820	8.00721536087660	0.32683041670900
H	8.31428450025488	8.89245228510281	-1.03690667394348
H	6.90748521685950	7.86444516393166	-1.30442843013810
C	10.04185651639838	7.08139962654206	-0.38381633164970
H	10.61534523984301	7.81110005233868	-0.96736163216371
H	9.87274557958427	7.52015562253452	0.60381883625002
C	10.82276291969246	5.78372149461367	-0.26387896009523
H	11.12311382483292	5.42442992284969	-1.25238166657529
H	11.74065603455975	5.95029238573185	0.31190697690898
C	9.91810691032672	4.96730655596960	1.84059266485426
H	9.38675931610417	4.14419957204850	2.32405985012763
H	10.92456054219948	5.00693929289140	2.27002355422446
H	9.41602796913040	5.91135105114248	2.07652793872272
C	10.55658902704808	3.43156584587030	0.10343679760515
H	10.05051793357072	2.65227844936282	0.67434177069299
H	10.48949286946469	3.20989742466234	-0.96569956402135
H	11.61533553690275	3.40915546355606	0.38186822183194
Pd	-7.95582231241615	-4.86707816021638	0.25423177733925
N	-9.57894657680014	-4.97434713639760	1.53895759246197
N	-8.96373527006329	-6.30871260434636	-0.84475617697380
C	-9.21901473043268	-5.05083793177625	2.95860733103302
H	-8.52629647297970	-5.87973831568960	3.13062812222146
H	-8.76724987932318	-4.11798808908024	3.29798484280155
H	-10.10964863720343	-5.22825863814994	3.57033820897117
C	-10.37514711475810	-3.76339659117107	1.28818810151843
H	-9.85169773849071	-2.88216133784571	1.66569689774566
H	-10.57443212157210	-3.63722771406920	0.21875205287813
C	-10.34866319690263	-6.19229604428844	1.18080428899773
H	-9.88872216837028	-7.05730809253278	1.66732209637746

H	-11.37718229038900	-6.12097149140556	1.55349719747611
C	-10.35393126372641	-6.37011628119411	-0.32804225401014
H	-10.82007068129495	-7.32693577157971	-0.59087614887787
H	-10.94438512504023	-5.58264757984036	-0.80521115220782
C	-9.01250754944483	-6.04272887165393	-2.28613530611458
H	-8.02054992382829	-6.12053663899252	-2.73242637218916
H	-9.65757836210186	-6.77178193216056	-2.78755846661648
H	-9.42559615556903	-5.04783044166736	-2.47583228920289
C	-8.25770613413403	-7.57390579454232	-0.59248991147871
H	-7.28015600389989	-7.56525851911255	-1.07957236943734
H	-8.12788634422031	-7.74591439262453	0.48123949869447
H	-8.82016166965037	-8.41972976601924	-1.00139634832720
Pd	4.81289307534666	-7.81818758538306	-2.40558669312407
N	6.58221510714423	-8.74922822314576	-1.85439797146652
N	4.61746341529104	-9.38863492624212	-3.74704835071581
C	7.72732242518622	-7.84136281195542	-1.72939955414364
H	7.85725255650420	-7.26442943752503	-2.64954962171581
H	7.59780347724480	-7.16247082774408	-0.88585443334035
H	8.64813019798390	-8.40871570507581	-1.55865249614961
C	6.31662257950389	-9.40061455729352	-0.56278481328759
H	5.40393986682169	-10.00424913150992	-0.60604760478221
H	7.14350975267148	-10.06244378597136	-0.28495753605322
H	6.21851057589423	-8.65111235737071	0.22526698925607
C	6.89248423272963	-9.75894649057643	-2.89751596262941
H	7.39305723329600	-9.26183349467933	-3.73350411857782
H	7.58606650963630	-10.51349330383067	-2.50828942974605
C	5.60935963952023	-10.42501864764292	-3.36473096277954
H	5.81851659992357	-11.08856340818827	-4.21195144352092
H	5.18646676670620	-11.04349125602965	-2.56787422798464

C	3.28556304837076	-10.00241839401428	-3.76733667030507
H	2.54332207137689	-9.31268414299877	-4.17057738648248
H	3.28540046770479	-10.89532633555960	-4.40112361237296
H	2.99375772559014	-10.30978134933260	-2.75901373554985
C	4.93247671238620	-8.83182775757562	-5.07155217655464
H	4.13502253744630	-8.16134161320209	-5.39930323729731
H	5.88200224472420	-8.28637505567966	-5.05390428344317
H	5.01800496945263	-9.62921833682231	-5.81707865787500
Pd	-1.39335461130282	1.85489313305670	-9.22114512372055
N	-1.29659854713218	3.48995575854533	-10.49085871588480
N	-1.94595313018087	0.80130803947387	-10.91773188492805
C	-2.49294126821261	4.29486873910370	-10.20092576983887
H	-3.39837269274105	3.67948968275362	-10.22845134026253
H	-2.40383065878668	4.76456875142583	-9.21872873450578
H	-2.60983610162734	5.09562659868204	-10.93854203825823
C	-0.09127328832925	4.30940080915626	-10.32764120988738
H	0.80526098777307	3.68959156061112	-10.42077823506679
H	-0.04484218442500	5.08057555933700	-11.10360216155567
H	-0.09057415625939	4.81525305748562	-9.36143018886527
C	-1.33358583981182	2.97552634660564	-11.88289368687838
H	-0.32339438411794	2.67570120305477	-12.17638787886173
H	-1.65086133197832	3.76343534739666	-12.57598293992827
C	-2.28562188540130	1.79445419472598	-11.96751737026334
H	-3.31875376931724	2.12329430118477	-11.82295899837973
H	-2.22853746766678	1.33751570400608	-12.96238939859865
C	-0.76661754313148	0.01400515298331	-11.30803712767595
H	-0.59560004571763	-0.79171511871504	-10.59071379111433
H	-0.91580136219924	-0.44715864156939	-12.28989935323705
H	0.12678300856507	0.64469616246804	-11.36675957978412

C	-3.09733376210886	-0.08591901230348	-10.72344283272216
H	-3.94429583478780	0.47386018611774	-10.31626850769477
H	-3.41232857885436	-0.51852818478034	-11.67874394124632
H	-2.84693509470755	-0.91081104733957	-10.05541961339349
N	5.04445298685209	-6.17561600969580	-1.03455673236016
N	2.56604835149436	-1.79805883585221	7.26900534325092
N	7.32994295686429	3.09640355034774	0.43504594249928
N	4.47987195461317	-2.81970365825655	2.69663613507553
N	4.97296079959344	-0.54790761048701	3.02000713750741
N	5.62174157983110	-1.61795478520915	1.03552377385197
N	-6.89392842542507	-3.37540618190551	1.41586983532458
N	-4.50976222974297	5.91295422603851	2.85644734985783
N	-0.19912701630431	-1.10096314033733	7.96517840313029
N	-4.76683492162812	0.87474030543230	3.14196922461110
N	-3.19406462875887	1.43640694460361	4.79137683916845
N	-3.81361528006162	-0.80391103425863	4.47624112294371
N	-1.51655116654368	0.16240242183361	-7.89566077043287
N	-6.29047042166015	-4.74281510372197	-1.09281788399640
N	2.96720684353173	-6.86436084086315	-2.97731197060132
N	-2.74945242477269	-3.15576171011468	-4.30358558268514
N	-1.66789243851017	-4.86457465558748	-3.11290436681014
N	-0.49038529256384	-3.62886644426675	-4.72352012377453
N	-0.79116424663828	2.94130109001673	-7.45935533968220
N	-3.20646339948685	7.45955298526943	0.75785420222095
N	6.02854751282148	5.27166727275461	-1.00855796880696
N	-0.62204976584846	5.27458605936926	-2.98320228444260
N	1.04304602825553	5.89599013668982	-1.45094921941640
N	1.62379570535204	4.74320882136159	-3.40990254325756
C	4.54692622308302	-6.23204918742094	0.20337720902683

H	4.11919754021189	-7.17991293482901	0.51632776339549
C	4.55896361542102	-5.15156329114799	1.07272009670807
H	4.15224665788045	-5.24736218434558	2.06556249968408
C	5.10064123894909	-3.93703613550369	0.64948112072636
C	5.64890313171304	-3.89654690173739	-0.63455955166340
H	6.10769436015787	-2.99175017718847	-0.99686354470508
C	5.60106773328956	-5.02743290190223	-1.43314894781230
H	6.03404699055058	-5.01497522041886	-2.42930709100478
C	3.22384868453365	-0.71318224468364	6.84663091534287
H	3.25883005660807	0.13240122509812	7.52759053890327
C	3.85233438765020	-0.64466768025409	5.61283076523027
H	4.38111130121773	0.24750464576613	5.32044387002707
C	3.81102604305435	-1.74715130927554	4.75516563310291
C	3.13822752446062	-2.88214589987502	5.21181898123664
H	3.09171568638803	-3.76590244853437	4.59749917032895
C	2.53667103160798	-2.86239869098506	6.46201161265661
H	2.01180820380510	-3.73776405707207	6.83178680686788
C	7.40649296170613	1.97333416013912	-0.28585039912457
H	7.94082075594773	2.03397675260258	-1.22934207239615
C	6.85234142233648	0.77056713422245	0.12815648371219
H	6.94914557958817	-0.11310678153838	-0.48136495467013
C	6.17788777440354	0.70719471579340	1.35011730905574
C	6.11650986514599	1.88201963368917	2.10302909732383
H	5.62686763648050	1.88205428816160	3.06255718628495
C	6.70062812505653	3.04025882470339	1.61286445221691
H	6.66982070445855	3.95850923427862	2.19337889168638
C	5.07437774662480	-2.73518575637168	1.50884460061446
C	4.45768732317094	-1.70686320978301	3.42484962001160
C	5.56325417415021	-0.55133546751741	1.82776484651797

C	-7.05358029837950	-2.07527357260015	1.15093048739671
H	-7.78965556844502	-1.81912569940029	0.39322596102644
C	-6.34255615582344	-1.07791163314384	1.80194514362965
H	-6.51916869684550	-0.04094111958707	1.56736342916290
C	-5.40951762844910	-1.42434535277698	2.78169793660328
C	-5.25409205585109	-2.78486360032008	3.05709325869479
H	-4.56078240275418	-3.10443747993095	3.81797920767793
C	-6.01031542751051	-3.71491558076226	2.35935429892186
H	-5.91264899350598	-4.77435511527204	2.57694343754512
C	-3.62314490820486	5.51251965889834	3.77242227131136
H	-3.04768640421996	6.29030094951920	4.26511669853200
C	-3.43797174502900	4.18091410926673	4.11408080182294
H	-2.72123282935648	3.90575790265176	4.87016816741090
C	-4.20350134759952	3.19534741645865	3.48698858051448
C	-5.13512704463250	3.62385307400198	2.53839794340396
H	-5.76822906231578	2.90748130247114	2.04114943555783
C	-5.25299787602181	4.97690352441564	2.25777427881951
H	-5.98214438225913	5.32407630590226	1.53004140152363
C	-1.06253005258473	-1.97371201537157	7.43973036078037
H	-0.96587438961827	-3.00808185753652	7.75455876734127
C	-2.04664268170253	-1.60852562461985	6.53415321376962
H	-2.72092469754750	-2.34837265304397	6.13664741621445
C	-2.15260202771011	-0.27463320396141	6.13693451132745
C	-1.28230971206194	0.64021314412344	6.73385778566421
H	-1.35140731822732	1.68772504035990	6.49198057273240
C	-0.32869920772080	0.18694627348883	7.63091377660855
H	0.35896541774190	0.88502284234871	8.10107001807813
C	-4.62763120863583	-0.39796350945176	3.50651883750348
C	-4.04981474928488	1.76312148818917	3.82700756643602

C	-3.11231312004504	0.14319966325713	5.09451771204535
C	-2.63549045682828	-0.11406806821020	-7.21992257058367
H	-3.51193650274096	0.47887448546887	-7.46423126120857
C	-2.70986239517847	-1.11275541081652	-6.26163576018801
H	-3.63606481128929	-1.30795775728807	-5.74740796017154
C	-1.57775900602888	-1.87837401333531	-5.97700550692478
C	-0.41918465615566	-1.59821979108157	-6.70395551258763
H	0.47381499878050	-2.17878741124613	-6.54200765782855
C	-0.43298727543338	-0.57760276520585	-7.64182670063630
H	0.45953712780866	-0.35058786228611	-8.21943236217523
C	-5.19184582120459	-5.47029864816631	-0.86998478281497
H	-5.24698769333887	-6.20577038413641	-0.07138229934412
C	-4.02697807777886	-5.32143994448275	-1.60615663833146
H	-3.16432289927685	-5.93165246856986	-1.39571501309639
C	-3.97797922980633	-4.37085918333879	-2.62742304062876
C	-5.14214544453611	-3.64259203709640	-2.87869943233951
H	-5.16598532249173	-2.92267326225029	-3.67974109919814
C	-6.26407128174521	-3.85862493318847	-2.09406103274597
H	-7.18016717616933	-3.30623924112001	-2.28186683954522
C	2.97101026560969	-5.89423429202103	-3.89753072995974
H	3.91072330669137	-5.71314652626114	-4.41285098047304
C	1.84716329221678	-5.14376367261066	-4.20750304654928
H	1.89402216444490	-4.37678202529190	-4.96296748553482
C	0.64696748045447	-5.39252432353905	-3.53622524421037
C	0.64612564431557	-6.42493255930656	-2.59645271168002
H	-0.26031286762953	-6.67508091840735	-2.07153795281779
C	1.81658584874815	-7.12810131515969	-2.35280097371788
H	1.82991193870817	-7.94098828546880	-1.63418698458902
C	-1.60813098706409	-2.94520414433746	-4.95427651708410

C	-2.73842338800919	-4.12629316616666	-3.39387496928962
C	-0.56716185503764	-4.59337200254547	-3.80940878994936
C	-1.71071058499985	3.45115600146135	-6.63449826435661
H	-2.74600285671964	3.38825187214335	-6.95872408317314
C	-1.39150016990586	4.04836238377673	-5.42422656299986
H	-2.16540092998824	4.45894203334316	-4.79720052202249
C	-0.05457693715725	4.12986332941869	-5.02844345829957
C	0.90109037668107	3.60772323547011	-5.90397550212074
H	1.94870063499073	3.66677006459379	-5.65738070751398
C	0.49221536168768	3.02972606262729	-7.09667052125390
H	1.22515856412390	2.63264947237236	-7.79302000680790
C	-1.90663815609333	7.56732036887734	1.04888286590233
H	-1.65437511169376	8.06128972886543	1.98376252156283
C	-0.90610843415675	7.08670234791887	0.21879121359466
H	0.13075352194827	7.20547423312551	0.48617506551022
C	-1.25223186622273	6.44759867442913	-0.97335933182936
C	-2.61028571476659	6.37532275610512	-1.28839791022579
H	-2.92675382723939	5.92512402889931	-2.21459551530062
C	-3.54376306076455	6.89139817106158	-0.40330925031594
H	-4.60334386013543	6.85458023962376	-0.63916933004445
C	5.66400210269921	4.77587126457169	-2.19492516140745
H	6.45411392915124	4.35445553834150	-2.81162680017744
C	4.35407302324449	4.78983022552279	-2.64763491943704
H	4.10689766195972	4.38607437205959	-3.61539549573453
C	3.35509328930219	5.33824108512159	-1.84113181531813
C	3.74973536466939	5.88258608583275	-0.61786774482015
H	3.02140585049032	6.34753297367628	0.02534966392460
C	5.08350933730070	5.82758677314740	-0.24524387259998
H	5.40800815646865	6.25614580069008	0.69826772667169

C	0.33571257829645	4.75172885055644	-3.74388225864829
C	-0.22450650775795	5.85246065607607	-1.85222023282728
C	1.93759723421942	5.33304440229444	-2.25912904199970
H	-11.33931608614612	-3.81892342049514	1.80418208606490
C	-0.82745658620278	0.71619837641666	2.62758530716912
C	-1.56272698569166	-0.36216727302776	2.16273951407527
C	-1.39827743623328	-1.61091729074024	2.72971918402856
C	-0.48082653919815	-1.79416724997097	3.76402089241650
C	0.26711104283382	-0.70834505401140	4.21809131314033
C	0.08836338876450	0.53972138878820	3.65279574810019
H	-0.96761452196290	1.69241374405944	2.19107962895018
H	-2.27090054672389	-0.22570601289529	1.35753220209817
H	-1.97345759966437	-2.45359470815155	2.37916106400327
H	0.97920863781762	-0.84849629632410	5.01821712960529
H	0.66414325809270	1.37976546911207	4.01114113369505
C	-0.31650094110171	-3.08034223879678	4.33696176965885
C	-0.18264583840542	-4.18466634714856	4.79059309884791
H	-0.08601504535771	-5.15300884217093	5.18765217037602
C	-3.04298934083355	-2.47481072941713	-0.29927233335590
C	-1.70987535897463	-2.72865782321692	-0.58337887948880
C	-1.03844012521330	-1.95297503171336	-1.50937675914007
C	-1.70566898451178	-0.92131236279069	-2.16971944009184
C	-3.04906463613763	-0.67447934602988	-1.88590845221627
C	-3.70858297282035	-1.44686043244257	-0.94891097964409
H	-3.56633111178905	-3.08047760299084	0.42707806591065
H	-1.18972167255771	-3.53216365523555	-0.08392816699752
H	0.00224126555638	-2.14168285532549	-1.72948530900381
H	-3.56570857261192	0.11953404481398	-2.40367791990504
H	-4.74704309618326	-1.24917912772617	-0.72638844723460

C	-1.02413203201806	-0.13372498650263	-3.13023536134403
C	-0.45680403501968	0.53438427219486	-3.95125559539910
H	0.05780183861126	1.11608866019164	-4.65700772237952
C	0.01368757333777	2.58137378343570	-0.73347643938533
C	1.05055083480415	3.04998644393399	0.07356498667723
C	0.76280412202881	3.87708414629233	1.15973631206065
C	-0.54728023244452	4.21805649911113	1.43822633843986
C	-1.57621881347430	3.75159254789415	0.63311592692936
C	-1.29205068967868	2.93887112118348	-0.45338183982210
H	0.23482689015670	1.93819375862552	-1.57327636181948
H	1.56744236240010	4.24011226246620	1.78061300654031
H	-0.76835397953423	4.84615470445469	2.28824918184683
H	-2.60009130828858	4.01876623099337	0.85342991220078
H	-2.08982781200197	2.57635992291666	-1.08367390464583
C	2.73064534837541	-2.12035507613275	-1.18361544987341
C	2.36281692921210	-3.43004776330658	-0.93824175663298
C	1.81092319623101	-3.78111709450478	0.29320037245756
C	1.64624388574331	-2.80427169270188	1.27524655843420
C	2.00512358852773	-1.49550586562269	1.01652955314174
C	2.54626940041506	-1.14911538119248	-0.21159128407935
H	3.15786362492871	-1.85417476194952	-2.13879946838541
H	2.50384515837414	-4.18723501484300	-1.69548536139880
H	1.23589268745858	-3.08618013658340	2.23231701984542
H	1.86451032676193	-0.73907515063420	1.77575224648794
H	2.81960714219504	-0.12297488876841	-0.40810191240503
C	2.38919958543302	2.68878804829021	-0.21551611620036
C	3.52410576833970	2.38803804861087	-0.46769004446999
H	4.50925802243191	2.11461800344183	-0.70401377559844
C	1.41336911557252	-5.11471916432859	0.55988181368440

C	1.05569484967508	-6.23378448965372	0.81031823839289
H	0.74419465942756	-7.20871826893230	1.04911187962524

References

1. W. Kabsch, *Acta Crystallogr., Sect. D: Biol. Crystallogr.*, 2010, **66**, 125–132.
2. W. Kabsch, *Acta Crystallogr., Sect. D: Biol. Crystallogr.*, 2010, **66**, 133–144.
3. G. M. Sheldrick, *Acta Crystallogr., Sect. A: Found. Adv.*, 2015, **71**, 3–8.
4. G. M. Sheldrick, *Acta Crystallogr., Sect. C: Struct. Chem.*, 2015, **71**, 3–8.
5. C. B. Hübschle, G. M. Sheldrick and B. Dittrich, *J. Appl. Crystallogr.*, 2011, **44**, 1281–1284.
6. L. J. Farrugia, *J. Appl. Crystallogr.*, 2012, **45**, 849–858.
7. A. L. Spek, *Acta Crystallogr., Sect. C: Struct. Chem.*, 2015, **71**, 9–18.
8. H. D. Flack, *Acta Crystallogr., Sect. A: Found. Crystallogr.*, 1983, **39**, 876–881.
9. A. L. Spek, *Acta Crystallogr., Sect. D: Biol. Crystallogr.*, 2009, **65**, 148–155.
10. R. Banerjee, P. Bhandari, N. Hickey and P. S. Mukherjee, *J. Am. Chem. Soc.*, 2025, **147**, 23049–23059.
11. R. Jia, J. Wang, Y. Jiang, B. Ni and T. Niu, *Org. Biomol. Chem.*, 2022, **20**, 8305–8312.
12. A. Nakamura, H. Kanou, J. Tanaka, A. Imamiya, T. Maegawa and Y. Miki, *Org. Biomol. Chem.*, **2018**, *16*, 541–544.
13. S. D. Friis, T. L. Andersen and T. Skrydstrup, *Org. Lett.*, **2013**, *15*, 1378–1381.
14. K. Hema, A. B. Grommet, M. J. Białek, J. Wang, L. Schneider, C. Drechsler, O. Yanshyna, Y. Diskin-Posner, G. H. Clever and R. Klajn, *J. Am. Chem. Soc.*, **2023**, *145*, 24755–24764.
